# Highly near-IR emissive ytterbium(iii) complexes with unprecedented quantum yields†
†Electronic supplementary information (ESI) available. CCDC 1501198–1501200. For ESI and crystallographic data in CIF or other electronic format see DOI: 10.1039/c6sc05021b



**DOI:** 10.1039/c6sc05021b

**Published:** 2017-01-13

**Authors:** Ji-Yun Hu, Yingying Ning, Yin-Shan Meng, Jing Zhang, Zhuo-Yan Wu, Song Gao, Jun-Long Zhang

**Affiliations:** a Beijing National Laboratory for Molecular Sciences , State Key Laboratory of Rare Earth Materials Chemistry and Applications , College of Chemistry and Molecular Engineering , Peking University , Beijing , 100871 , P. R. China . Email: gaosong@pku.edu.cn ; Email: zhangjunlong@pku.edu.cn; b College of Materials Science and Opto-Electronic Technology , University of Chinese Academy of Sciences , Beijing , 100049 , P. R. China

## Abstract

The design of highly near-infrared (NIR) emissive lanthanide (Ln) complexes is challenging, owing to the lack of molecular systems with a high sensitization efficiency and the difficulty of achieving a large intrinsic quantum yield. Previous studies have reported success in optimizing individual factors and achieving high overall quantum yields, with the best yield being 12% for Yb(iii). Herein we report a series of highly NIR emissive Yb complexes, in which the Yb is sandwiched between an octafluorinated porphyrinate antenna ligand and a deuterated Kläui ligand, which allowed optimization of two factors in the same system, and one of the complexes had an unprecedented quantum yield of 63% (estimated uncertainty 15%) in CD_2_Cl_2_ with a long lifetime (*τ*_obs_) of 714 μs. Systematic analysis of the structure–photophysical properties relationship suggested that porphyrinates are effective antenna ligands with a sensitization efficiency up to *ca.* 100% and that replacement of the high-energy C–H oscillators in porphyrinate and Kläui ligands significantly improves the intrinsic quantum yield up to 75% (*τ*_obs_/*τ*_rad_), both of which contribute to enhancing the NIR emission intensity of Yb(iii) up to 25-fold. Besides the high luminescence efficiency, these Yb complexes have other attractive features such as excitation in the visible range and large extinction coefficients which make these Yb(iii) complexes outstanding optical materials in the NIR region.

## Abstract 2

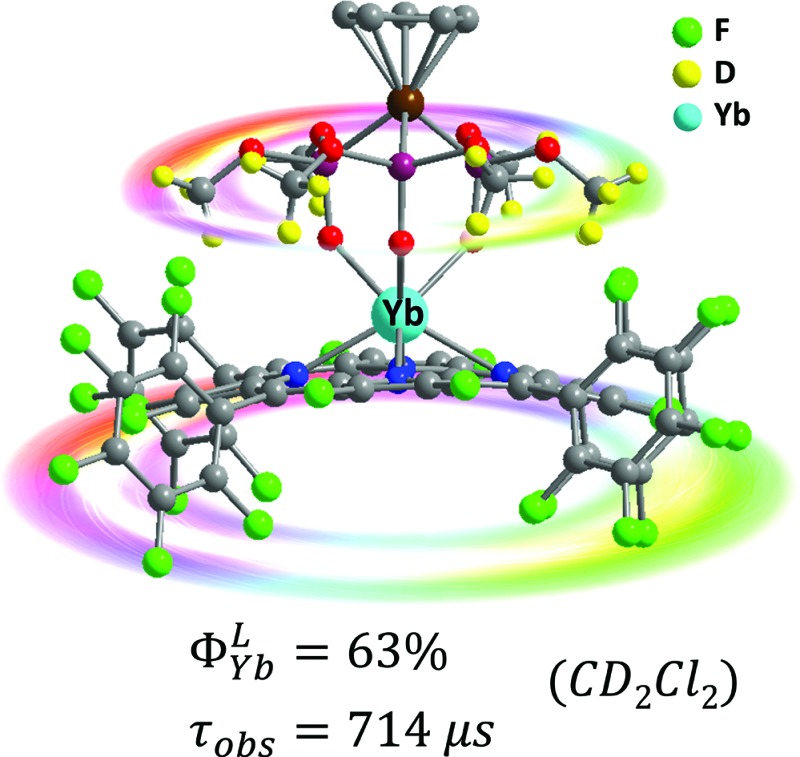
We report a series of highly NIR emissive Yb complexes, in which the Yb is sandwiched between an octafluorinated porphyrinate antenna ligand and a deuterated Kläui ligand, and one of the complexes has an unprecedented quantum yield of 63%.

## Introduction

Highly NIR emissive trivalent lanthanide compounds such as Er, Nd and Yb complexes have been sought after by generations of chemists because of their many potential applications in telecommunications,^1^ light-emitting devices^2^ and biological imaging.^2b,3^ However the forbidden electric dipole f–f transitions make this task difficult.^4^ One way to break this bottleneck is to take advantage of the light-harvesting ability of organic fluorophores to sensitize the lanthanide emission *via* energy transfer, but it has been shown to be quite challenging to implement this strategy.^5^ For example, the highest overall quantum yield reported for ytterbium complexes is 12%, achieved with a deuterated ytterbium cryptate in perdeuterated methanol by Seitz *et al.* ^6^ Thus it is still of importance to seek an appropriate sensitization system for further improving the NIR emission significantly.1
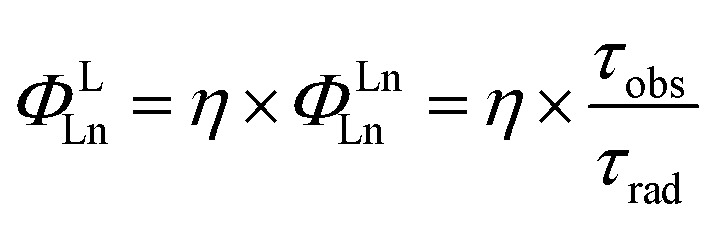



According to eqn (1), which defines the overall quantum yield *Φ*LLn, an ideal antenna molecular system should meet both the requirements of a high sensitization efficiency (*η*) and large intrinsic quantum yield (*Φ*LnLn). Toward this goal, most reports had focused on increasing either the sensitization efficiency or the intrinsic quantum yield through molecular design. For the sensitization efficiency, porphyrinates (Por) had been shown to be “ideal” candidates among the diverse antenna ligands because of (1) the intense absorption in the visible region; (2) tunable triplet states above the excited state levels of NIR emissive Yb ions with an energy gap in the 2000–3000 cm^–1^ range; and (3) a good chelating ability.^7^ Despite a high sensitization efficiency (close to 100%) being achieved, the overall luminescence quantum yields of Yb porphyrinates are still low (<5%),^8^ most likely due to detrimental C–H oscillators in the vicinity of the Yb ion lowering the intrinsic quantum yields.^9^

For the intrinsic quantum yield, as early as the 1960s, Kropp and Windsor^10^ and Horrocks *et al.* ^11^ observed that solvent O–H bonds quench Ln emissions, and later Haas, Stein and Würzberg interpreted this quenching effect in the context of energy gap law.^12^ Thus, using heavier atoms such as D and F to replace the H atom of X–H (X = C, N, O) oscillators in the antenna ligand became an alternative approach to enhance the quantum yields through extending the luminescence lifetime *τ*_obs_. These early findings led to the design of many successful ligands for NIR emissive Ln complexes, such as bis(perfluorooctylsulfonyl)amide,^13^ bis(pentafluorophenyl)phosphinic acid,^14^ perfluorinated imidodiphosphinate (*τ*_obs_ up to millisecond for Yb),^15^ perdeuterated cryptands^6,16^ and so on. In addition, shortening the radiative lifetime (*τ*_rad_) by changing the coordination sphere could also increase *Φ*LnLn.^6,17^ However, these reported complexes have a low absorption in the visible region or a high-lying energy-donor state, which are disadvantageous for efficient energy transfer from the ligands to the NIR emissive Ln center. Therefore, on the basis of the tremendous progress in the optimization of *η* and *Φ*LnLn [eqn (1)], integrating the best of these components by using a good light harvesting antenna such as a porphyrin and depleting the X–H oscillators is consequently a promising approach for achieving highly NIR emissive lanthanide complexes.

In this work, we report that a high sensitization efficiency and a large intrinsic quantum yield can be simultaneously obtained using a molecular system with a perfluorinated porphyrin, 2,3,7,8,12,13,17,18-octafluoro-5,10,15,20-tetrakis(pentafluorophenyl)porphyrin (**1**), as the sensitizer and a partially deuterated Kläui's tripodal ligand **[D_18_]-L_OMe_**, [(cyclopentadienyl)tris(di(methyl-d_3_)phosphito)cobaltate]^18^ as the capping ligand. Using this approach, we are able to dramatically increase the overall quantum yield of the corresponding Yb(iii) complex **[D_18_]-1-Yb** up to 63% (estimated uncertainty 15%) and extend the lifetime to 714 μs in CD_2_Cl_2_. Given the large extinction coefficient of the porphyrinate (*ε* ∼ 320 000 M^–1^ cm^–1^) in the visible to red region, **[D_18_]-1-Yb** represents one of the brightest NIR-emissive Ln(iii) complexes (brightness: *ε* × *Φ*LLn ∼ 190 000 M^–1^ cm^–1^) ever reported upon excitation in the visible range (>400 nm). Systematic analysis of the structure–photophysical properties relationship suggests the importance of both a porphyrinate antenna ligand and a C–H oscillator free coordination environment for designing highly NIR emissive Yb complexes. Furthermore, the subtle effects that *meso*-phenyl groups of the β-octafluorinated porphyrins have on the NIR emission properties of Yb(iii) complexes are anticipated to enable synthetic flexibility and practicality for the further design of functional materials that emit in the NIR region.

## Results and discussion

### Synthesis and crystal structures

**Fig. 1:**
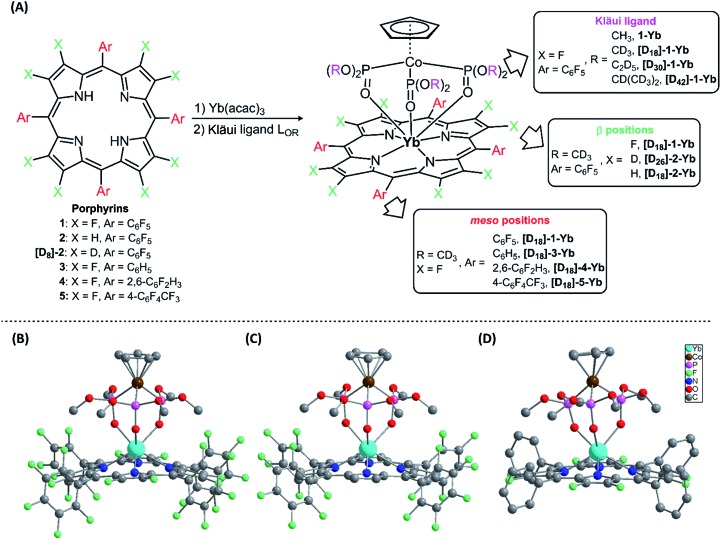
(A) Synthesis of the Ln(iii) complexes; (B)–(D) perspective drawings of **1-Yb**, **2-Yb** and **[D_18_]-3-Yb**, respectively, with hydrogen atoms omitted for clarity.

Seven-coordinate Ln(iii) complexes, in which the Ln ion is sandwiched between porphyrin and Kläui ligands, with the general formula [(Por)Ln(L_OR_)] had been known since 2001 and studied beyond their luminescence properties.^8a,19^ In this work, we chose 2,3,7,8,12,13,17,18-octafluoro-5,10,15,20-tetrakis(pentafluorophenyl)porphyrin (**1**) as an antenna ligand to investigate the effect of C–H oscillators on porphyrinates, using 5,10,15,20-tetrakis(pentafluorophenyl)porphyrin (**2**) and 2,3,7,8,12,13,17,18-octaduetero-5,10,15,20-tetrakis(pentafluorophenyl)porphyrin (**3**) as controls. To assess the effects of the *meso*-pentafluorophenyl group in **1**, we replaced it with 2,6-difluorophenyl (**4**) and 4-trifluoromethylphenyl (**5**) groups. As for the Kläui tripodal ligand, except for the original one, (cyclopentadienyl)tris(dimethylphosphito)cobaltate (**L_OMe_**), three alkoxide chain deuterated analogues **[D_18_]-L_OMe_**, **[D_30_]-L_OEt_** and **[D_42_]-L_OiPr_** were used. As shown in Fig. 1A, ten Yb(iii) complexes were synthesized according to previously reported procedures.^19a,20^ These structures were unambiguously confirmed using NMR, HR-ESI-MS, elemental analysis, UV-Vis absorption and IR spectra (details are given in the ESI†). The NMR studies and UV-Vis absorption spectra show the good stability of these complexes as there is only one species in solution.

Three representative ytterbium complexes **1-Yb**, **2-Yb** and **[D_18_]-3-Yb** were also crystallographically characterized (Fig. 1B–D). In these complexes, the Yb ion is seven-coordinate, and surrounded by four N atoms from the porphyrinate dianion and three O atoms from the Kläui ligand. The three mean planes (C5 of the cyclopentadienyl ring, N4 of the porphyrin, and O3 of the phosphito groups) are almost parallel to each other. The bond lengths of Yb–N (2.35–2.38 Å) and Yb–O (2.20–2.27 Å) and the distances between the Yb and the N4 mean plane (1.16–1.19 Å) in the three compounds are very similar, with a difference of < 0.1 Å (ESI Table S2†), consistent with similar previously reported ytterbium porphyrinate complexes.^8a,19a,21^

### Photophysical properties

**Fig. 2:**
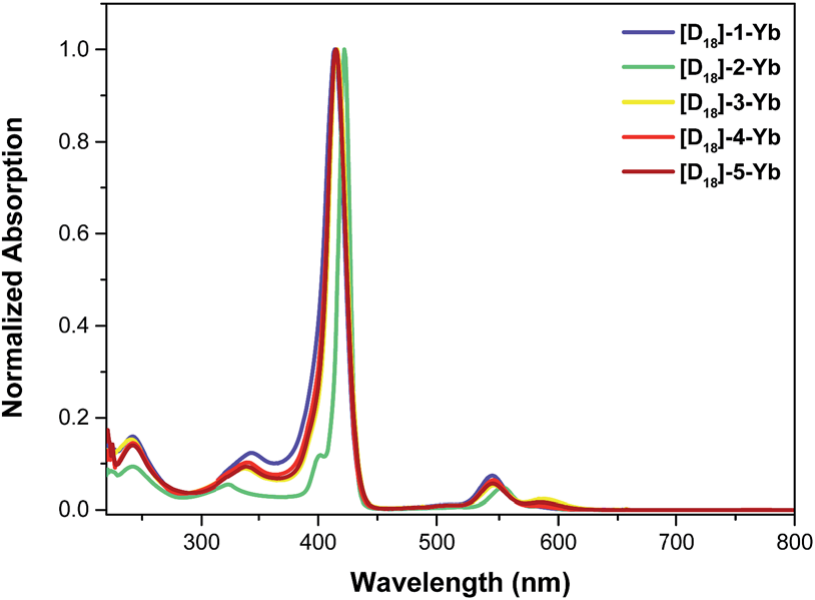
UV-vis absorption spectra of the Yb complexes with different porphyrin ligands in CH_2_Cl_2_.

The photophysical properties of the Yb complexes were investigated using UV-vis absorption and photoluminescence spectroscopy. All the complexes displayed an intense Soret band at 380–450 nm and moderately intense Q bands at 520–600 nm in dichloromethane, which are the characteristic absorptions of Ln(iii) porphyrinates (Fig. 2 and ESI, Fig. S56–S57†).^22^ Introducing eight fluorine atoms into the β-positions of the porphyrin resulted in hypsochromic shifts of *ca.* 8 nm for both the Soret and Q bands (**[D_18_]-1-Yb** *vs.* **[D_18_]-2-Yb**). **[D_18_]-1-Yb**, **[D_18_]-3-Yb**, **[D_18_]-4-Yb**, and **[D_18_]-5-Yb** display nearly identical Soret bands at ∼415 nm and Q bands at ∼545 nm, indicating that the *meso*-aryl groups have a subtle effect on the electronic structures. Increasing the steric hindrance at the Kläui ligands leads to slight red shifts (up to *ca.* 4 nm) of both the Soret and Q bands of the ytterbium complexes **[D_18_]-1-Yb**, **[D_30_]-1-Yb** and **[D_42_]-1-Yb** (ESI Fig. S56†). As expected, deuteration of the Kläui ligand or at the β-positions of the porphyrin does not affect the electronic structures of the Yb complexes, which display identical absorption spectra (ESI Fig. S57†).

**Fig. 3:**
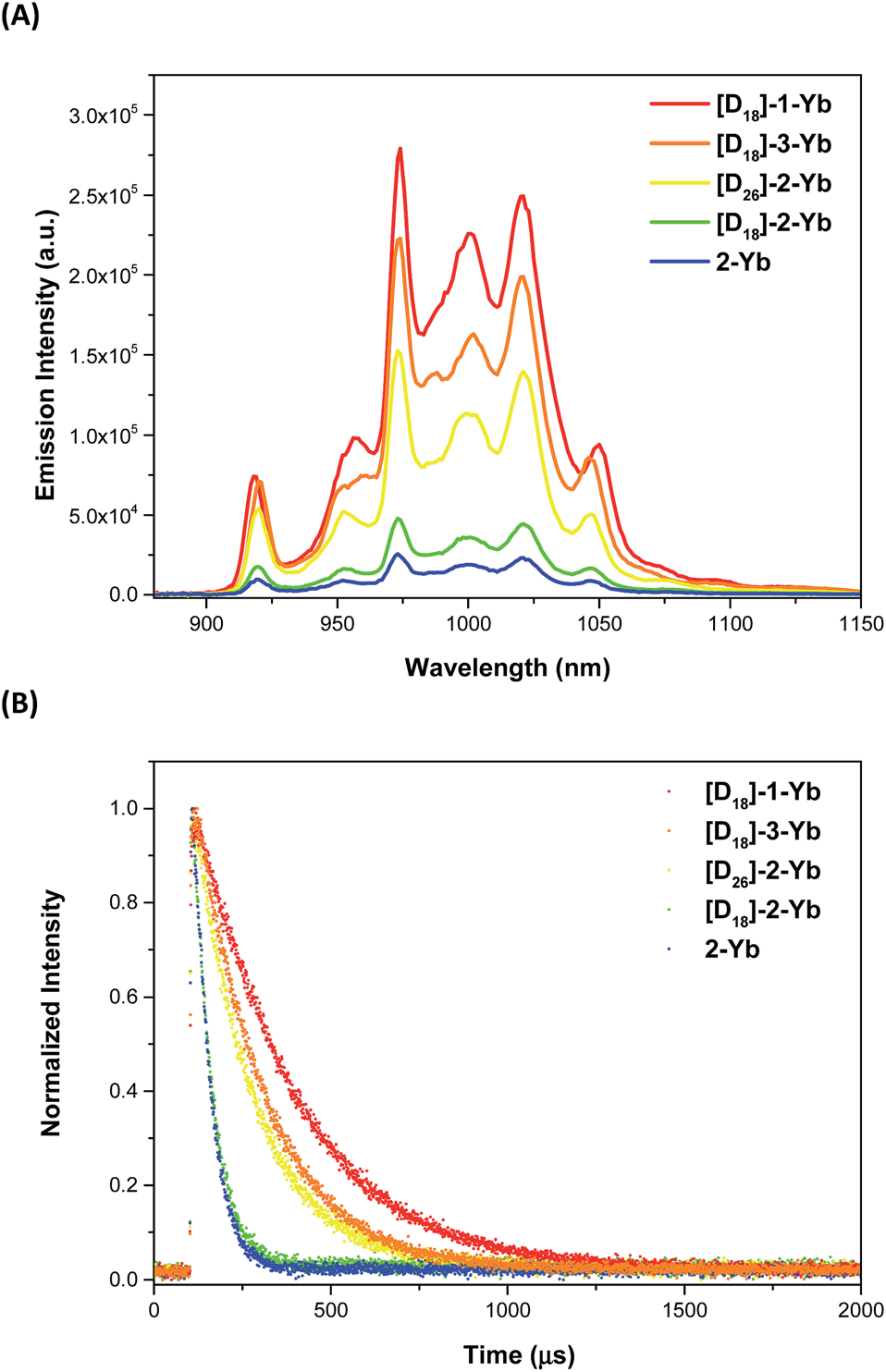
(A) Emission spectra of the ytterbium complexes with various degrees of C–H bond replacement under the same conditions (*λ*_ex_ = 425 nm, absorbance = 0.10); (B) decay of the NIR luminescence of the ytterbium complexes.

Excitation within the Soret band region at 425 nm yields a typical NIR emission centred at 974 nm for all the Yb(iii) complexes, which is assigned to the Yb(^2^F_5/2_ → ^2^F_7/2_) transition. The excitation spectra are consistent with the corresponding absorption spectra, suggesting energy transfer from the porphyrinate ligands to the Yb(iii) centers (ESI, Fig. S58–67†). Emission in the visible region (520–780 nm) was also observed, which was much weaker compared to the free bases, again pointing to the energy absorbed by the porphyrinate chromophore being efficiently transferred to the metal centers.^8a,d,22a,23^ Interestingly, under the same photoluminescence conditions, the emission intensity was found to decrease in the order **[D_18_]-1-Yb** > **[D_18_]-3-Yb** > **[D_26_]-2-Yb** > **[D_18_]-2-Yb** > **2-Yb**, indicating that β-fluorination of the porphyrin and deuteration of the Kläui ligand improve the NIR emission of Yb significantly (Fig. 3A). Accordingly, the lifetimes of these Yb complexes follow the same order as the luminescence intensity (Fig. 3B).

In order to quantify these results, the NIR emission quantum yields (*Φ*LYb) and lifetimes (*τ*_obs_) of the Yb(iii) complexes were measured and the data are summarized in Table 1. It is worth noting that the estimated uncertainties in *Φ*LYb are 15%.^24^ The *Φ*LYb values for the NIR emission were obtained using a comparative method with **Yb(TPP)(L_OEt_)** (*Φ* = 2.4% in CH_2_Cl_2_, H_2_TPP = 5,10,15,20-tetraphenylporphyrin; L_OEt_ = [(cyclopentadienyl)tris(diethylphosphito)cobaltate])^8*a*^ as a reference, using a FLS920 spectrometer (Edinburgh Instruments) equipped with a PMT R5509-73 detector (300–1700 nm, Hamamastu) for NIR emission. Among all the Yb complexes, **[D_18_]-1-Yb** displays overall quantum yields of 25% in CH_2_Cl_2_ and 63% in CD_2_Cl_2_ (ESI, Fig. S87†), which are much higher than the quantum yield of the Yb(iii) cryptate reported by Seitz *et al.* in 2015 (12% in CD_3_OD).^6^ These values were further confirmed using a real photon counting method, using an integrating sphere and the same instrument, which gave *Φ*LYb values of 26% in CH_2_Cl_2_ and 69% in CD_2_Cl_2_ respectively (ESI, Fig. S88–S89†). Since the sensitivity of the PMT detector is lower within the optical window of 900–1100 nm than a CCD detector,^25^ we also measured the quantum yields of **[D_18_]-1-Yb** using a Fluorolog-3 spectrofluorimeter equipped with a CCD detector (Horiba Scientific, see Experimental section for details).^25^ In CH_2_Cl_2_, the quantum yield of **[D_18_]-1-Yb** was 23%, close to that obtained using the PMT 5509-73 detector. The quantum yield in CD_2_Cl_2_ was 49%, which is lower than that obtained using the PMT detector, probably due to the CCD detector having a cutoff at 1100 nm, whereas **[D_18_]-1-Yb** still has a strong emission beyond this region (Fig. 3A). Nevertheless, after consideration of the quantum yields obtained using different methods, the overall quantum yields of **[D_18_]-1-Yb** in CH_2_Cl_2_ and CD_2_Cl_2_ are still much higher than those of the previously reported Yb complexes.^26^ The lifetimes of **[D_18_]-1-Yb** are as long as 285 μs in CH_2_Cl_2_ and 714 μs in CD_2_Cl_2_, and much longer than those of most reported ytterbium complexes (typically below 100 μs).^8c,15b,27^ Taking the high extinction coefficient of the porphyrinate ligand (*ε*_414 nm_ ≈ 320 000 M^–1^ cm^–1^) into account, **[D_18_]-1-Yb** represents one of the brightest NIR emissive Ln complexes (brightness: *ε* × *Φ*LYb ∼ 190 000 M^–1^ cm^–1^) ever reported upon excitation in the visible range (>400 nm).

**Table 1:** Luminescence lifetimes (*τ*_obs_), NIR quantum yields (*Φ*LYb) and triplet energies *E*(T_1_) of the Yb(iii) complexes described in this study^*a*^

Complex	*τ*_obs_ ^*a*^/μs		*Φ* L Yb ^*b*^/%		*E*(T_1_)^*e*^/cm^–1^
	CH_2_Cl_2_	CD_2_Cl_2_	CH_2_Cl_2_	CD_2_Cl_2_	
**1-Yb**	49(1)	54(1)	5.1(0.2)	5.7(0.2)	*ca.* 13 500
**[D_18_]-1-Yb**	285(9)	714(20)	25(1), 26^*c*^, 23^*d*^	63(3), 69^*c*^, 49^*d*^	
**[D_30_]-1-Yb**	273(4)	—^*f*^	24(2)	—^*f*^	
**[D_42_]-1-Yb**	281(5)	—^*f*^	25(1)	—^*f*^	
**2-Yb**	29(1)	33(2)	2.4(0.1)	2.5(0.1)	*ca.* 13 700
**[D_18_]-2-Yb**	54(1)	65(2)	3.9(0.2)	5.1(0.2)	
**[D_26_]-2-Yb**	180(2)	401(5)	15(1)	29(1)	
**[D_18_]-3-Yb**	197(6)	449(21)	20(2)	42(2)	*ca.* 12 100
**[D_18_]-4-Yb**	249(3)	592(5)	23(1)	58(3)	*ca.* 13 100
**[D_18_]-5-Yb**	233(3)	500(5)	20(1)	51(2)	*ca.* 12 250

^*a*^Standard error values are given in parentheses; they refer to the reproducibility of the measurements. The estimated uncertainties in the quantum yield are 15%. Experimental relative errors: *τ*_obs_, ±5%; *Φ*LYb, ±10%.

^*b*^Determined using a comparative method and referenced to **Yb(TPP)(L_OEt_)** (*Φ* = 2.4% in CH_2_Cl_2_) unless noted.

^*c*^Determined in an integrating sphere on a FLS920 instrument using a PMT R5509-73 detector (300–1700 nm).

^*d*^Determined in an integrating sphere on a Fluorolog-3 instrument with a CCD detector (1024 × 256 pixel, 200–1100 nm), referenced to **Yb(TPP)(L_OEt_)**.

^*e*^Estimated from the phosphorescence of a corresponding Gd(iii) complex (ESI Fig. S68).^31^

^*f*^Not determined.

The higher quantum yield of **[D_18_]-1-Yb** compared to the other Yb complexes unambiguously demonstrates that C–H oscillators at the β-pyrrolic positions (Yb–H distance 5.29–5.39 Å) and *meso*-phenyl positions of the porphyrin (Yb–H distance 5.00–6.21 Å) as well as in the phosphito group of the Kläui ligand (Yb–H distance 4.01–5.64 Å) effectively quench the NIR emission of Yb. Yet C–H oscillators at the *meso*-phenyl positions of the porphyrin only had a minor impact on the NIR emission, despite the similar Yb–H distances (5.00–8.66 Å). The Yb complexes with β-fluorinated porphyrin and deuterated Kläui ligands (**[D_18_]-3-Yb**, **[D_18_]-3-Yb**, **[D_18_]-4-Yb**, and **[D_18_]-5-Yb**) exhibit very high quantum yields (>20% in CH_2_Cl_2_ and >40% in CD_2_Cl_2_). The C–H oscillators at the β-positions of the porphyrin are detrimental to the NIR luminescence of Yb(iii), as evidenced by the large drop in quantum yield on going from **[D_18_]-1-Yb** to **[D_18_]-2-Yb** and from **1-Yb** or **[D_26_]-2-Yb** to **2-Yb**. Moreover, β-fluorination is more effective than β-deuteration for increasing the NIR emission, as shown by the much higher quantum yield of **[D_18_]-1-Yb** compared to **[D_26_]-2-Yb**. This is probably due to the lower vibration energy of C–F (∼1200 cm^–1^) compared to that of C-D oscillators (∼2300 cm^–1^), consequently resulting in a smaller vibrational quenching rate.^12a,28^ The C–H oscillators of the phosphito groups of the Kläui ligand also decrease the Yb(iii) NIR emission dramatically, as evidenced from comparing the data for **[D_18_]-1-Yb** *vs.* **1-Yb** as well as **[D_18_]-2-Yb** *vs.* **2-Yb**. On the other hand, almost identical quantum yields were obtained for **[D_18_]-1-Yb** (25%), **[D_30_]-1-Yb** (24%) and **[D_42_]-1-Yb** (25%) in CH_2_Cl_2_, indicating that steric congestion at the Kläui ligand does not help to improve the quantum yield for Yb.^29^ Outer-sphere C–H oscillators, such as those from solvent molecules, deactivating the excited state of NIR emissive lanthanides is a common phenomenon.^30^ A significant solvent isotopic effect was observed for **[D_18_]-1-Yb** and **[D_26_]-2-Yb** (>2 fold increase of *Φ*LLn on going from CH_2_Cl_2_ to CD_2_Cl_2_), compared to a much smaller effect observed for **1-Yb**, **2-Yb** and **[D_18_]-2-Yb** (+4 to +31%). We conclude that, in the absence of high-energy C–H oscillators in the inner coordination sphere, the emissive state of Yb(iii) is deactivated mainly through solvent C–H vibrational relaxation, as substantiated by the significant isotopic solvent effect observed.

**Fig. 4:**
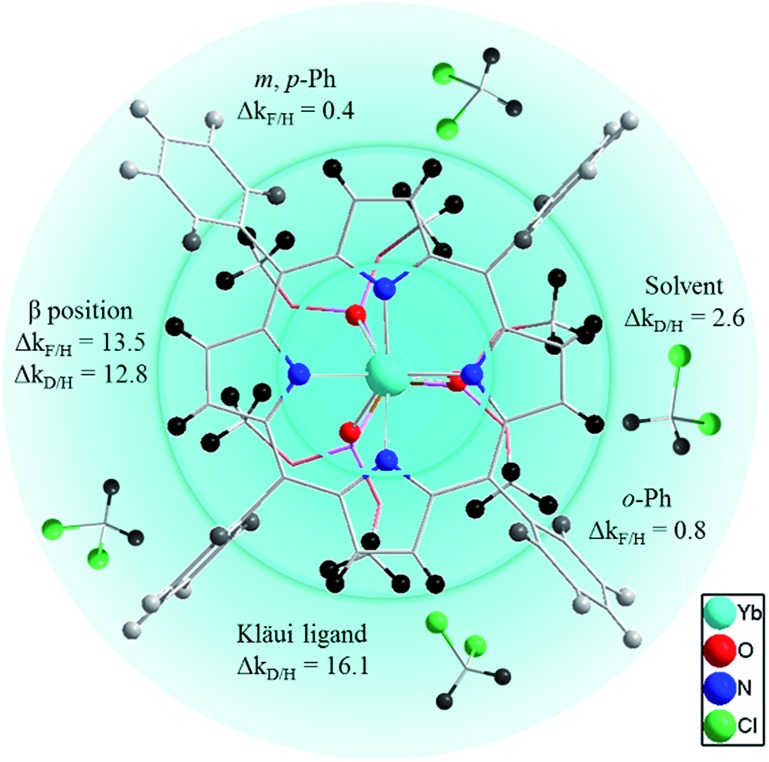
C–H oscillator quenching sphere of the [Yb(Por)(LOR)] complexes (top view from the porphyrin plane side) showing the quenching rate differences Δ*k* (in ms^–1^) of C-(H/D) or C-(H/F) at different sites. Hydrogen atoms are colored from black to grey indicating a decreased quenching rate difference.

To better elucidate the deactivation ability of C–H oscillators, we have elaborated a quenching sphere model for use with the **[(Por)Yb(L_OR_)]** complexes studied (Fig. 4). The estimated quenching rate differences Δ*k* (in ms^–1^) for C-(H/D) or C-(H/F) at different sites, calculated from the lifetimes of the corresponding complexes, are also shown (see ESI for details, Table S5†). According to the Δ*k* values, the coordination sphere is divided into three layers, within which the gradually fading blue color indicates weakening of the quenching effects of the C–H oscillators in the sphere (not strictly distance-dependent). The coordinating N and O atoms are in the primary sphere. The C–H oscillators at β-pyrrolic positions of the porphyrinate (Δ*k*_F/H_ = 13.5 ms^–1^ and Δ*k*_D/H_ = 12.8 ms^–1^) and the phosphito groups of the Kläui ligands (Δ*k* = 16.1 ms^–1^) with a large Δ*k* value fit into the second sphere. They account for the major vibrational quenching contribution in the Yb complexes. The C–H oscillators of the *meso*-phenyl groups play a role in the third sphere, with much smaller Δ*k* values (0.8 ms^–1^ for *ortho*-positions and 0.4 ms^–1^ for *meta*- and *para*-positions respectively). The higher quenching rate for *ortho*-C–H bonds than that of *meta*- or *para*-C–H bonds is probably due to the shorter distances to the Yb center for the former (5.00–6.21 Å) compared to those of the latter (7.33–8.86 Å). The C–H bonds of solvent molecules interact with the excited state of Yb formally in the third sphere with Δ*k*_D/H_ (Δ*k*_D/H_ = 2.6 ms^–1^) comparable to that of the *meso*-phenyl ones.

### Sensitization efficiency and intrinsic quantum yield

Having established the influence of C–H oscillators on the emission properties of the ytterbium porphyrinate complexes, we started to investigate the key factors governing improvement of the luminescence efficiency for the present molecular system. According to eqn (1), the overall quantum yield *Φ*LYb is determined by two components: the sensitization efficiency (*η*) and intrinsic quantum yield *Φ*YbYb. The latter is calculated from the ratio of non-radiative deactivation process (*τ*_obs_) to the radiative lifetime (*τ*_rad_).^26^ *τ*_rad_ of Yb(iii) can be estimated from f–f transition absorption spectra based on a modified Einstein equation:^32^
2
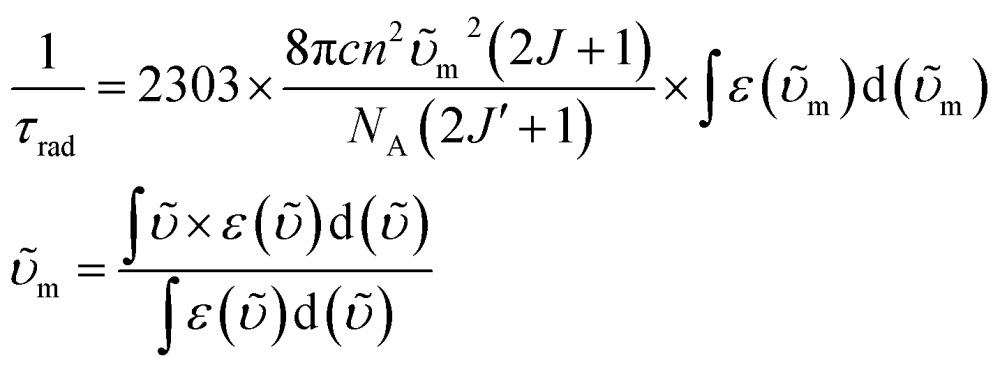
where *c* is the speed of light in cm s^–1^, *n* is the refractive index (*n*(CD_2_Cl_2_) = 1.442), *N*_A_ is Avogadro's number, *J* and *J*′ are the quantum numbers for the ground and excited states, respectively, 
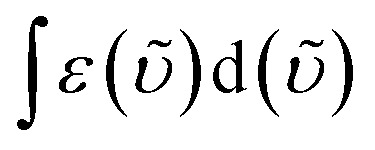
 is the integrated spectrum of the ^2^F_7/2_ → ^2^F_5/2_ transition, and *υ̃*_m_ is the barycenter of the transition.

Given that these Yb(iii) complexes share identical N_4_O_3_ coordination environments, including similar Yb–N and Yb–O bond lengths, the radiative lifetimes of these complexes are not expected to vary greatly.^6,17a^ Thus we chose **[D_18_]-3-Yb** as an example to measure *τ*_rad_ in CD_2_Cl_2_ because of its good solubility up to 10^–2^ M (ESI Fig. S92†). The derived radiative lifetime of *τ*_rad_ = 0.95 ms (∼15% error), which is within the typical range of 0.5–1.3 ms for ytterbium complexes in solution,^9d,27c,32–33^ was used for all the Yb(iii) complexes to calculate the sensitization efficiency and intrinsic quantum yield, and the results are tabulated in Table 2.

**Table 2:** Intrinsic quantum yield and sensitization efficiency of the Yb complexes^*a*^

Compound	*Φ* Yb Yb /%	*η*/%
**1-Yb**	5.7(0.7)	100(13)
**[D_18_]-1-Yb**	75(9.6)	84(11)
**2-Yb**	3.5(0.5)	71(10)
**[D_18_]-2-Yb**	6.9(0.9)	74(10)
**[D_26_]-2-Yb**	42(5.3)	69(9)
**[D_18_]-3-Yb**	47(6.3)	90(13)
**[D_18_]-4-Yb**	62(7.7)	93(13)
**[D_18_]-5-Yb**	52(6.5)	97(13)

^*a*^Solvent: CD_2_Cl_2_. *τ*_rad_ = 0.95 ms (see text). The standard errors are given in parentheses. Experimental relative errors: *Φ*YbYb, ±15%; *η*, ± 15%.

**Fig. 5:**
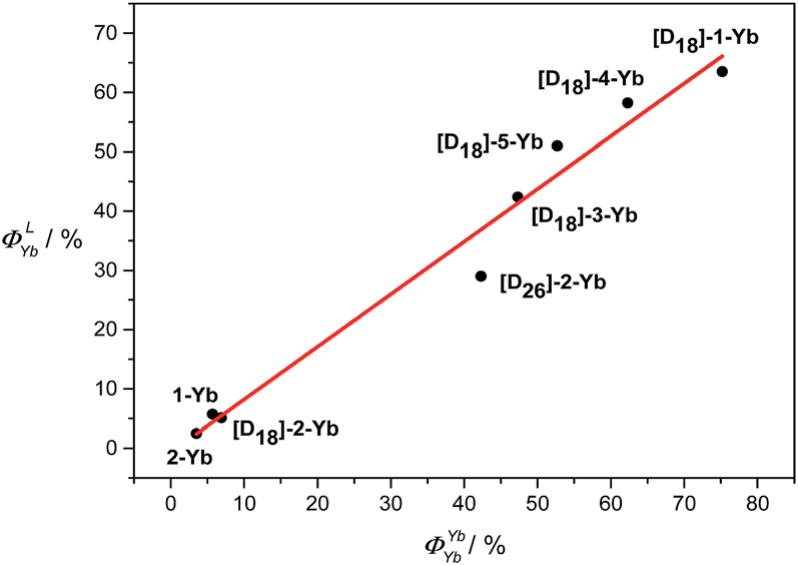
Approximate linear relationship of *Φ*LYb*vs. Φ*YbYb in CD_2_Cl_2_.

All the Yb(iii) complexes have high *η* values (>70%) in CD_2_Cl_2_, suggesting that β-fluorination or deuteration of the ligands has a subtle effect on the energy transfer process from the lowest triplet state (T_1_) of the ligands to the Yb(iii) excited state. The T_1_ energy levels of these compounds were determined from low-temperature emission spectra of the corresponding Gd(iii) complexes and are in the range 12 100–13 700 cm^–1^, which is considered to be optimum for efficient Yb sensitization.^4*b*^ Besides, the short distances between the Yb and the porphyrin N4 mean plane determined from the crystal structure are also favourable for efficient energy transfer. Thus, the sensitization efficiency is not the main factor responsible for the pronounced differences in the NIR emissions found in this work between these Yb complexes. In contrast, the intrinsic quantum yields for the Yb(iii) complexes are significantly different from each other (3.5–75% in CD_2_Cl_2_, estimated error 15%), as a result of disparity in *τ*_obs_, which is highly related to the degree of C–H oscillator substitution. Among them, **[D_18_]-1-Yb** has the highest intrinsic quantum yield ever reported, *ca.* 75% in CD_2_Cl_2_, owing to its extremely long lifetime. Moreover, plotting *Φ*LYb*vs. Φ*YbYb shows an approximately linear relationship (Fig. 5), suggesting a decisive role of *Φ*YbYb in determining the *Φ*LYb of the Yb(iii) porphyrinates. Therefore, minimization of the non-radiative processes *via* fluorination and deuteration is the main origin of the increased quantum yields, which reach a maximum for the nearly C–H bond free compound **[D_18_]-1-Yb**.

## Conclusion

In summary, we report here a molecular system for achieving highly luminescent Yb(iii) complexes with a new benchmark quantum yield of 63% (estimated uncertainty 15%). Systematic analysis of the photophysical properties and the structures of the complexes revealed that a C–H bond depleted coordination sphere is critical for obtaining a high NIR emission efficiency, as a result of minimized non-radiative processes. The β-pyrrolic C–H bonds of the porphyrin and the phosphito C–H bonds of the Kläui ligand greatly influence the Yb(iii) luminescence, whereas those of the *meso*-phenyl group substituents on the porphyrin only have a slight effect. Fluorination of the porphyrin ligand was shown to have a much more beneficial effect than deuteration. In addition to the high quantum yield, other attractive features of these compounds such as excitation in the visible range, large extinction coefficients and synthetic flexibility make them easily adaptable for the design of potential light converting systems.

## Experimental section

### General materials and methods

UV-vis spectra were recorded using an Agilent 8453 UV-vis spectrometer equipped with an Agilent 89090A thermostat (±0.1 °C) at 25 °C. Near-IR absorption spectra were recorded using a Shimadzu UV-3600 Plus UV-Vis-NIR Spectrophotometer. Mass spectra were recorded using a Bruker APEX IV FT-ICR mass spectrometer (ESI-MS). Elemental analyses (C, H, N) were performed using an Elementar Analysensysteme GmbH vario EL Elemental Analyzer. NMR spectra were recorded using a Varian Mercury Plus 300 MHz spectrophotometer or Bruker ARX400 400 MHz spectrophotometer. IR spectra were recorded using a Bruker VECTOR22 FTIR spectrometer and KBr pellets. For the optical measurements in liquid solution, spectroscopic grade CD_2_Cl_2_ was purchased from Cambridge Isotope Laboratories, Inc. and used as received. Anhydrous CH_2_Cl_2_ was distilled from calcium hydride and 1,2,4-trichlorobenzene (TCB) was purchased from J&K Scientific. The β-octafluorinated porphyrin ligands^34^ and deuterated Kläui's ligand^18*b*^ (D atom > 99%) were synthesized according to literature methods.

### Synthesis of lanthanide porphyrinates

The syntheses were carried out according to modified literature methods.^19a,20a^ Generally, a porphyrin (0.03 mmol) and Ln(acac)_3_·*n*H_2_O (0.15 mmol) were refluxed in 8 mL of TCB for 2 h under N_2_. During the reaction process, the luminescence of the porphyrin free base gradually vanished. After cooling to room temperature, the reaction mixture was eluted with petroleum ether, CH_2_Cl_2_, and CH_2_Cl_2_/MeOH = 5/1 sequentially to provide TCB, the unreacted porphyrin free base and the lanthanide porphyrin complexes, in order, using flash silica gel chromatography. The lanthanide complex and 1.2 equiv. of the Kläui ligand L_OR_ (or the partially deuterated one) were stirred in 10 mL of CHCl_3_/MeOH (1/1) at 60 °C for 2 h. Then the product, with the general formula **[Ln(Por)(L_OR_)]**, was isolated using silica gel chromatography and recrystallized from CH_2_Cl_2_/*n*-hexane.

### Photophysical properties measurement

The emission spectrum and lifetime were recorded using an Edinburgh Analytical Instrument FLS920 lifetime and steady state spectrometer (450 W Xe lamp/microsecond flash lamp, PMT R928 for the visible emission spectrum, PMT R5509-73 with a C9940-02 Hamamatsu cooler for the NIR emission spectrum and luminescence lifetime). All the emission spectra in the NIR region were corrected using a calibration curve for the detector response (Fig. S55†). The NIR quantum yields of all the complexes were measured using a comparative method with **Yb(TPP)(L_OEt_)** as the reference after excitation at *λ*_ex_ = 425 nm (2.4%, CH_2_Cl_2_ solution). Sample quantum yields were evaluated using the following equation:
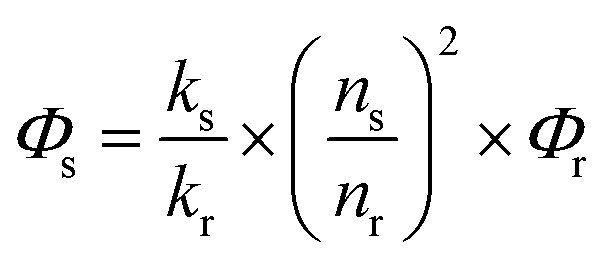
where the subscripts r and s denote the reference and sample respectively, *Φ* is the quantum yield, *k* is the slope from the plot of integrated emission intensity *vs.* absorbance, and *n* is the refractive index of the solvent. The estimated error for the quantum yield measurements is 15%.

The quantum yield of **[D_18_]-1-Yb** was also determined using integrating spheres and two instruments. The first was an integrating sphere (150 mm, PTFE inner surface) fitted within the Edinburgh Analytical Instrument FLS920 with a PMT R5509-73 detector for NIR emission and a PMT R928 for visible emission. The second was a Quanta-φ integrating sphere (150 mm, PTFE inner sphere, Horiba Scientific) along with a Horiba-Jobin-Yvon Fluorolog-3 spectrofluorimeter equipped with a CCD detector (1024 × 256 pixel, 200–1100 nm, Horiba Scientific) referenced to **Yb(TPP)(L_OEt_)**. The quantum yields determined with the FLS920 were evaluated according to the following equation:
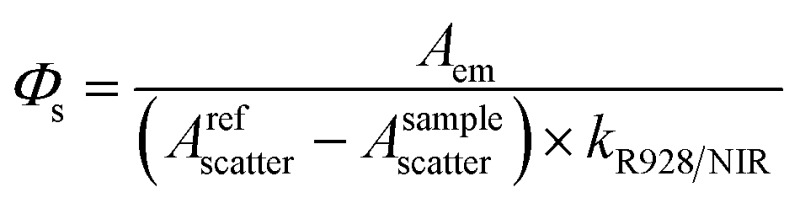
where *A*_em_ is the integrated area of the sample's emission (corrected); *A*refscatter and *A*samplescatter are the integrated areas under the Rayleigh scattering peaks of the reference sample and the sample under study; and *k*_R928/NIR_ is the ratio of the sensitivities of the two detectors. The value of *k*_R928/NIR_ was determined straight after the measurement.
