# Supplementary material for: Highly near-IR emissive ytterbium(iii) complexes with unprecedented quantum yields
Source: Chem Sci. 2017 Jan 13;8(4):2702–9. doi: 10.1039/c6sc05021b (PMC5480304; doi:10.1039/c6sc05021b)
Supplement: Supplementary file 1 [file SC-008-C6SC05021B-s001.pdf]

Electronic Supplementary Material (ESI) for Chemical Science.
This journal is © The Royal Society of Chemistry 2017

Supplementary Information

Highly Near-IR-Emissive Ytterbium(III) Complexes with Unprecedented Quantum Yields

Ji-Yun Hu[1], Yingying Ning[1], Yin-Shan Meng[1], Jing Zhang[2], Zhuo-Yan Wu[1], Song Gao[1]* and Jun-Long Zhang[1]*

[1]Beijing National Laboratory for Molecular Sciences, State Key Laboratory of Rare Earth Materials Chemistry and Applications, College of Chemistry and Molecular Engineering, Peking University, Beijing 100871, P. R. China

[2]Beijing National Laboratory for Molecular Sciences, Institute of Chemistry, Chinese Academy of Sciences, Beijing 100190, P. R. China

Correspondence should be addressed to J.-L. Zhang (Email: zhangjunlong@pku.edu.cn) and S. Gao (email: gaosong@pku.edu.cn).

Table of contents

1. General.....6

2. Synthesis.....6

2.1 Porphyrins and Kläui's ligand.....6

2.2 Porphyrinate lanthanide complexes.....6

2.3 Summary of Crystallographic Data.....11

2.4 NMR spectra.....15

2.5 HR-ESI-MS spectra.....25

2.6 IR spectra......33

3. Photophysical properties.....36

3.1 General.....36

3.2 Absorption, excitation and steady-state emission spectra.....38

3.3 Luminescence decay profiles.....43

3.4 NIR quantum yield measurement.....49

3.4.1 Results via FLS 920 – Comparative Method.....49

3.4.2 Results *via* FLS 920 – Integrating Sphere.....50

3.4.3 Results *via* Fluorolog-3 spectrofluorimeter.....52

3.5 Calculation of the quenching rate difference Δk.....53

3.6 Radiative luminescence lifetime τ_{rad} measurement.....54

4. References.....55

1. General

UV/Vis spectra were recorded on an Agilent 8453 UV/Vis spectrometer equipped with an Agilent 89090A thermostat (0.18C). Near-IR absorption spectra were recorded on a SHIMADZU UV3600PLUS spectrometer. Mass spectra were recorded on Bruker APEX IV FT-ICR mass spectrometer (ESI-MS) Elemental analyses (C, H, N) were recorded on Elementar Analysensysteme GmbH vario EL Elemental Analyzer. NMR spectra were recorded on a Varian Mercury Plus 300 MHz spectrophotometer or Bruker ARX400 400MHz spectrophotometer. IR spectra were recorded on a Bruker VECTOR22 FTIR spectrometer as KBr pellets. Intensity data for crystals were collected on a Bruker Smart Apex II CCD diffractometer with graphite-monochromated Mo Kα radiation (0.71073 Å) at 180 K or 277 K. The structures were solved by direct methods and refined with the full-matrix least-squares technique based on F^2 using the SHELXL program. All non-hydrogen atoms were refined anisotropically. Hydrogen atoms were placed at the calculation positions. Emission spectra and lifetime measurements were recorded on an Edinburgh Analytical Instruments FLS920 lifetime and steady-state spectrometer (450 W Xe lamp/microsecond flash lamp, PMT R928 for visible emission spectrum; Hamamatsu R5509-73 PMT with C9940-02 cooler for NIR emission spectrum,).

CH_2Cl_2 was distilled from calcium hydride. CD_2Cl_2 (99.8% D) was purchased from Cambridge Isotope Laboratories, Inc. and used as received. Anhydrous 1,2,4-trichlorobenzene (TCB) was purchased from J&K Scientific and used as received.

2. Synthesis

2.1 Porphyrins and Kläui's ligand

β-octafluorinated porphyrins[1] 2,3,7,8,12,13,17,18-Octafluoro-5,10,15,20-tetrakis(pentafluorophenyl)porphyrin (F28TPP), 2,3,7,8,12,13,17,18-Octafluoro-5,10,15,20-tetrakis(phenyl)porphyrin (F8TPP), 2,3,7,8,12,13,17,18-Octafluoro-5,10,15,20-tetrakis(*p*-chlorophenyl)porphyrin, 2,3,7,8,12,13,17,18-Octafluoro-5,10,15,20-tetrakis(4-trifluoromethylphenyl)porphyrin, 2,3,7,8,12,13,17,18-Octafluoro-5,10,15,20-tetrakis(2,6-difluorophenyl)porphyrin and deuterated Kläui's ligand[2] (D atom > 99%) were synthesized according to literature methods.

2.2 Porphyrinate lanthanide complexes

Porphyrinate lanthanide complexes were prepared via literature reports.[3] Porphyrin (0.03 mmol) and $Ln(acac)_3 \cdot nH_2O$ (0.15 mmol) in 8 mL 1,2,4-trichlorobenzene (TCB) were refluxed for 2 h under N_2. During the reaction process, the luminescence of porphyrin free base gradually vanished. After cooling to room temperature, the reaction mixture was eluted with petroleum ether, CH_2Cl_2, CH_2Cl_2/MeOH (5/1) sequentially to give TCB, unreacted porphyrin free base and lanthanide porphyrin complexes in order by flash silica gel chromatography. The lanthanide complex and two equivalent Kläui ligand L_{OR} were reacted at 60 °C in 10 mL $CHCl_3$/MeOH (1/1) for 1.5 h. Then the product with the general formula (F_nTPP)Ln(LOR) was isolated by silica gel chromatography and

recrystallized from CH_2Cl_2 / n-hexane.

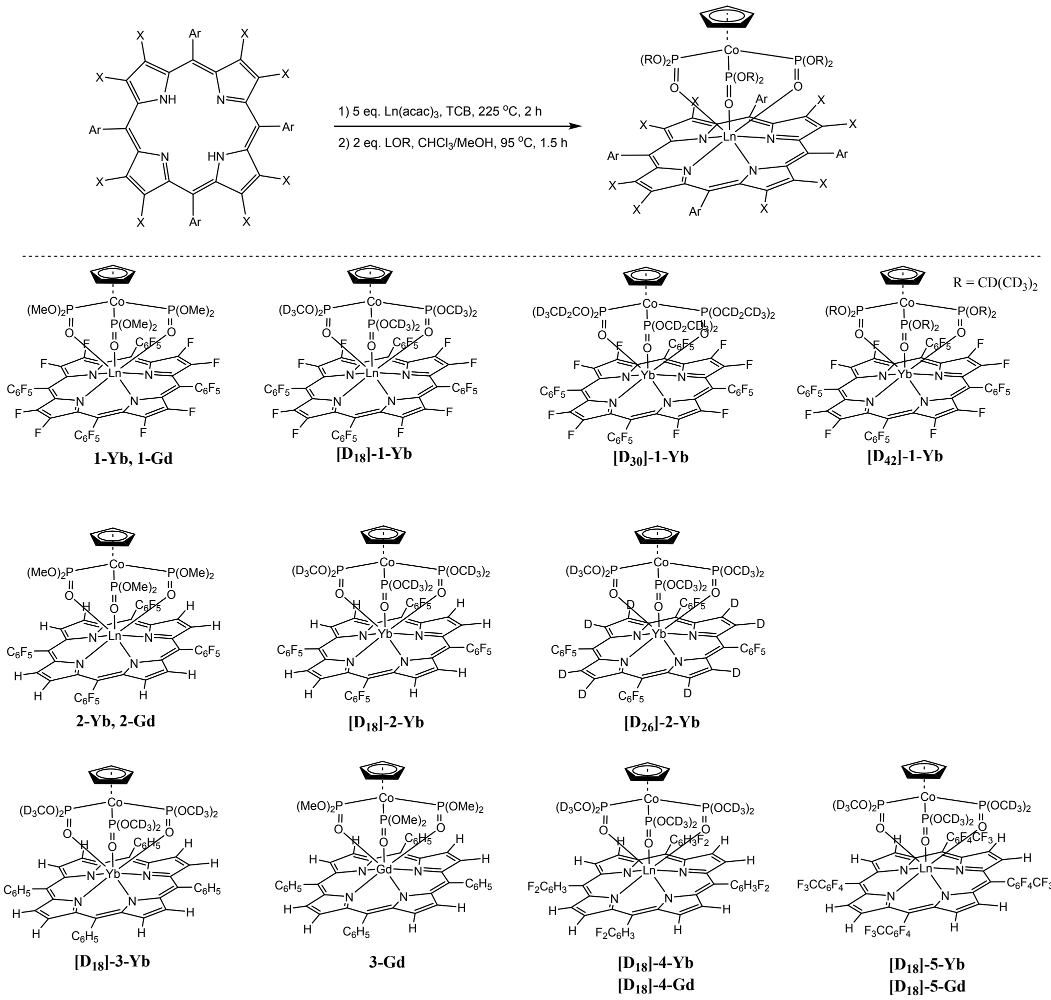

Figure S1. Synthesis of porphyrinate lanthanide complexes.

Complex **1-Yb**. Yield 85% (over two steps, based on the amount of porphyrin free base). Crystal for X-ray analysis was obtained by slow evaporation hexane into a $CHCl_3$ solution.

^{1}H NMR (300 MHz, $CDCl_3$) δ 6.47 (s, 18H), -5.51 (s, 5H). ^{19}F NMR (282 MHz, $CDCl_3$) δ -131.04 (d, *J* = 20.8 Hz, 4F), -136.92 (s, 8F), -139.72 (dd, *J* = 23.7, 5.5 Hz, 4F), -150.33 (t, *J* = 20.7 Hz, 4F), -160.22 (t, *J* = 18.2 Hz, 4F), -161.49 (t, *J* = 19.3 Hz, 4F). ESI-HR-MS: Calcd for $C_{55}H_{24}CoF_{28}N_4O_9P_3Yb$, [M+H$^+$]: 1741.90296. Found: 1741.90253. Anal. Calcd. for $C_{55}H_{23}CoF_{28}N_4O_9P_3Yb$: C, 37.95; H, 1.33; N, 3.22. Found: C, 38.16; H, 1.42; N, 3.24. IR (KBr, cm^{-1}): 2950, 2361, 1673, 1526, 1500, 1466, 1431, 1335, 1176, 1124, 1108, 1047, 1014, 991, 960, 846, 801, 786, 738, 676, 633, 591. UV/Vis (CH_2Cl_2, 25 °C): λ_{max} (log ε, M^{-1} cm^{-1}): 414(5.60), 544(4.25).

Complex **[D$_{18}$]-1-Yb**. Yield 67% (over two steps, based on the amount of porphyrin free base).

^{1}H NMR (400 MHz, $CDCl_3$) δ -5.60 (s, 5H). ^{19}F NMR (377 MHz, $CDCl_3$) δ -130.52 (dd, *J* = 23.4, 7.7 Hz, 4F), -136.32 (s, 8F), -139.21 (dd, *J* = 24.0, 7.6 Hz, 4F), -149.80 (t, *J* = 20.4 Hz, 4F), -159.68 (t, *J* = 18.4 Hz, 4F), -160.97 (t, *J* = 20.2 Hz, 4F). ESI-HR-MS: Calcd for $C_{55}H_6D_{18}CoF_{28}N_4O_9P_3Yb$, [M+H$^+$]: 1760.01594. Found: 1760.01884. IR (KBr, cm^{-1}): 3056, 2243, 2074, 1659, 1600, 1463, 1444, 1360,

1329, 1172, 1146, 1115, 1071, 1042, 1023, 1007, 920, 846, 779, 747, 700, 663, 608, 576. UV/Vis (CH_2Cl_2, 25 °C): λ_{max} (log ε, M^{-1} cm^{-1}): 414(5.51), 544(4.23).

Complex **[D_{30}]-1-Yb**. Yield 70% (over two steps, based on the amount of porphyrin free base).
^{1}H NMR (400 MHz, $CDCl_3$) δ -5.72 (s, 5H). ^{19}F NMR (377 MHz, $CDCl_3$) δ -131.13 (d, J = 18.5 Hz, 4F), -137.09 (s, 8F), -139.42 (dd, J = 24.2, 7.5 Hz, 4F), -149.92 (t, J = 20.4 Hz, 4F), -160.21 (t, J = 18.7 Hz, 4F), -161.00 (t, J = 19.4 Hz, 4F).ESI-HR-MS: Calcd for $C_{61}H_6D_{30}CoF_{28}N_4O_9P_3Yb$, [M+H$^+$]: 1856.18516. Found: 1856.18881. IR (KBr, cm^{-1}): 2295, 2854, 2231, 1671, 1526, 1499, 1467, 1430, 1362, 1335, 1262, 1175, 1127, 1111, 1091, 1050, 1010, 991, 959, 929, 842, 801, 729, 696, 676, 619, 578, 492, 420. UV/Vis (CH_2Cl_2, 25 °C): λ_{max} (log ε, M^{-1} cm^{-1}): 416(5.39), 546(4.13).

Complex **[D_{42}]-1-Yb**. Yield 40% (over two steps, based on the amount of porphyrin free base). The poor solubility of the compound prevents molar extinction coefficient determination. ^{1}H NMR (400 MHz, $CDCl_3$) δ -6.12 (s, 5H). ^{19}F NMR (377 MHz, $CDCl_3$) δ -129.52 (m, 4F), -137.49 (s, 8F), -140.30 – -140.67 (m, 4F), -150.04 (t, J = 20.4 Hz, 4F), -160.28 (m, 4F), -160.95 – -161.42 (m, 4F). ESI-HR-MS: Calcd for $C_{77}H_6D_{42}CoF_{28}N_4O_9P_3Yb$, [M+H$^+$]: 1952.35438. Found: 1952.35762. IR (KBr, cm^{-1}): 2233, 1675, 1640, 1527, 1499, 1466, 1430, 1334, 1262, 1230, 1175, 1119, 1105, 1069, 1047, 992, 975, 960, 935, 916, 836, 801, 750, 676, 635, 608, 577, 526, 473. UV/Vis (CH_2Cl_2, 25 °C): λ_{max} (log ε, M^{-1} cm^{-1}): 420, 551.

Complex **1-Gd**. Yield 57% (over two steps, based on the amount of porphyrin free base). Crystal for X-ray analysis was obtained by slow evaporation hexane into a $CHCl_3$ solution.
ESI-HR-MS: Calcd for $C_{55}H_{24}CoF_{28}N_4O_9P_3Gd$, [M+H$^+$]: 1725.88820. Found: 1725.89035. Anal. Calcd. for $C_{55}H_{23}CoF_{28}N_4O_9P_3Gd$: C, 38.30; H, 1.34; N, 3.25. Found: C, 38.71; H, 1.54; N, 3.25. IR (KBr, cm^{-1}): 2950, 1668, 1525, 1501, 1459, 1430, 1333, 1175, 1140, 1121, 1106, 1047, 1013, 991, 958, 847, 801, 785, 736, 675, 632, 590. UV/Vis (CH_2Cl_2, 25 °C): λ_{max} (log ε, M^{-1} cm^{-1}): 414(5.45), 544(4.22).

Complex **2-Yb**. Yield 74% (over two steps, based on the amount of porphyrin free base).
^{1}H NMR (400 MHz, $CDCl_3$) δ 14.96 (s, 8H), 6.34 (s, 18H), -4.98 (s, 5H). ^{19}F NMR (377 MHz, $CDCl_3$) δ -128.39 (d, *J* = 20.8 Hz, 4F), -136.42 (dd, *J* = 24.9, 8.0 Hz, 4F), -151.61 (t, *J* = 20.4 Hz, 4F), -159.42 – -160.44 (m, 4F), -161.43 (t, *J* = 20.6 Hz, 4F). ESI-HR-MS: calcd for $C_{55}H_{32}CoF_{20}N_4O_9P_3Yb$, [M+H$^+$]: 1597.97834; Found: 1597.97870. Anal. Calcd. for $C_{55}H_{31}CoF_{20}YbN_4O_9P_3$ +CH_3OH: C, 41.29; H, 2.17; N, 3.44; found: C, 42.43; H, 2.39; N, 3.44. IR (KBr, cm^{-1}): 2947, 2841, 1727, 1650, 1528, 1519, 1487, 1442, 1377, 1331, 1286, 1158, 1131, 1075, 1047, 1012, 988, 935, 836, 801, 759, 733, 710, 620, 588, 529, 412. UV/Vis (CH_2Cl_2, 25 °C): λ_{max} (log ε, M^{-1} cm^{-1}): 422(5.77), 553(4.44).

Complex **[D_{18}]-2-Yb.** Yield 72% (over two steps, based on the amount of porphyrin free base).
^{1}H NMR (400 MHz, $CDCl_3$) δ 14.96 (s, 8H), -4.99 (s, 5H).^{19}F NMR (377 MHz, $CDCl_3$) δ -128.44 (d, *J* = 18.2 Hz, 4F), -136.41 (dd, *J* = 24.7, 8.0 Hz, 4F), -151.61 (t, *J* = 20.5 Hz, 4F), -159.75 – -160.46 (m, 4F), -161.18 – -161.84 (m, 4F). ESI-HR-MS: calcd for $C_{55}H_{14}D_{18}CoF_{20}N_4O_9P_3Yb$, [M+H$^+$]: 1616.09132; Found: 1616.09175. IR (KBr, cm^{-1}): 2923, 2852, 2246, 2076, 1728, 1651, 1530, 1520,

1486, 1443, 1377, 1331, 1267, 1137, 1120, 1074, 1047, 1025, 987, 956, 843, 835, 801, 760, 745, 703, 581. UV/Vis (CH_2Cl_2, 25 °C): λ_{max} (log ε, M^{-1} cm^{-1}): 422(5.77), 553(4.44).

Complex **[D_{26}]-2-Yb.** Yield 70% (over two steps, based on the amount of porphyrin free base). The isotopic purity of 2,3,7,8,12,13,17,18-octadeuterium-5,10,15,20-tetrakis(pentafluorophenyl)porphyrin was estimated to be > 90% based on ^1H NMR spectroscopy analysis.

^1H NMR (400 MHz, $CDCl_3$) δ -4.90 (s, 5H). ^{19}F NMR (377 MHz, Chloroform-*d*) δ -128.51 (d, *J* = 24.3 Hz, 4F), -136.40 (d, *J* = 23.6 Hz, 4F), -151.64 (t, *J* = 20.4 Hz, 4F), -160.18 (t, *J* = 18.7 Hz, 4F), -161.44 (t, *J* = 22.0 Hz, 4F). ESI-HR-MS: calcd for $C_{55}H_6D_{26}CoF_{20}N_4O_9P_3Yb$, [$M+H^+$]: 1624.14153; Found: 1624.13842. IR (KBr, cm^{-1}): 2361, 2077, 1649, 1523, 1520, 1496, 1433, 1330, 1143, 1075, 1041, 1025, 988, 944, 866, 839, 747, 703, 581. UV/Vis (CH_2Cl_2, 25 °C): λ_{max} (log ε, M^{-1} cm^{-1}): 422(5.57), 553(4.28), 588 (3.52).

Complex 2-Gd. Yield 50% (over two steps, based on the amount of porphyrin free base). ESI-HR-MS: calcd for $C_{55}H_{32}CoF_{20}GdN_4O_9P_3$, [$M+H^+$]: 1581.96358. Found: 1581.96435; Anal. Calcd. for $C_{55}H_{31}CoF_{20}GdN_4O_9P_3$+$CH_3OH$: C, 41.70; H, 2.19; N, 3.47. Found: C, 42.55; H, 2.31; N, 3.54. IR (KBr, cm^{-1}): 2946, 1650, 1525, 1498, 1330, 1125, 1075, 1047, 1013, 988, 935, 836, 800, 759, 731, 588. UV/Vis (CH_2Cl_2, 25 °C): λ_{max} (log ε, M^{-1} cm^{-1}):422(5.80), 553(4.45).

Complex **[D_{18}]-3-Yb**. Yield 50% (over two steps, based on the amount of porphyrin free base). Crystal for X-ray analysis was obtained by slow evaporation of a CH_2Cl_2/MeOH (1/1) solution.

^1H NMR (300 MHz, $CDCl_3$) δ 16.99 (s, 4H), 10.66 (s, 4H), 9.56 (s, 4H), 8.99 (s, 8H), -5.22 (s, 5H). ^{19}F NMR (282 MHz, Chloroform-*d*) δ -132.93 (s, 8F). ESI-HR-MS: Calcd for $C_{55}H_{26}D_{18}CoF_8N_4O_9P_3Yb$, [$M+H^+$]: 1400.20438. Found: 1400.20522. IR (KBr, cm^{-1}): 2926, 2856, 2248, 2079, 1673, 1526, 1500, 1467, 1431, 1362, 1335, 1176, 1133, 1113, 1068, 1048, 1026, 991, 960, 921, 845, 801, 750, 730, 708, 676, 633, 609, 582, 528. UV/Vis (CH_2Cl_2, 25 °C): λ_{max} (log ε, M^{-1} cm^{-1}): 416(5.58), 546(4.20), 587 (3.89).

Complex **3-Gd**. Yield 20% (over two steps, based on the amount of porphyrin free base). Crystal for X-ray analysis was obtained by slow evaporation of a CH_2Cl_2/MeOH (1/1) solution.

ESI-HR-MS: Calcd for $C_{55}H_{43}CoF_8GdN_4O_9P_3$, [$M+H^+$]: 1366.07664. Found: 1366.07605. Anal. Calcd. for $C_{55}H_{43}CoF_8GdN_4O_9P_3$: C, 48.39; H, 3.18; N, 4.10. Found: C, 48.20; H, 3.29; N, 3.83. IR (KBr, cm^{-1}): 2944, 2360, 2342, 1654, 1601, 1459, 1444, 1356, 1327, 1172, 1147, 1131, 1111, 1038, 1006, 847, 778, 731, 701, 682, 661, 586. UV/Vis (CH_2Cl_2, 25 °C): λ_{max} (log ε, M^{-1} cm^{-1}): 417(5.54), 547(4.13), 588(3.85)

Complex **[D_{18}]-4-Yb.** Yield 93% (over two steps, based on the amount of porphyrin free base).

^1H NMR (400 MHz, $CDCl_3$) δ 10.51 (t, *J* = 7.6 Hz, 4H), 9.45 (s, 4H), 8.15 (t, *J* = 7.9 Hz, 4H), -5.42 (s, 5H). ^{19}F NMR (377 MHz, $CDCl_3$) δ -101.65 (s, 4F), -110.73 (s, 4F), -137.61 (s, 8F). ESI-HR-MS: calcd for $C_{55}H_{18}D_{18}CoF_{16}N_4O_{11}P_3Yb$, [$M+H^+$]: 1544.12900; Found: 1544.12715. IR (KBr, cm^{-1}): 2078, 1669, 1626, 1591, 1465, 1360, 1328, 1276, 1237, 1172, 1145, 1116, 1069, 1027, 1003, 922, 845, 792, 748, 705, 663, 606, 580, 510. UV/Vis (CH_2Cl_2, 25 °C): λ_{max} (log ε, M^{-1} cm^{-1}):

415(5.50), 546(4.25).

Complex **[D$_{18}$]-4-Gd.** Yield 97% (over two steps, based on the amount of porphyrin free base). ESI-HR-MS: calcd for $C_{55}H_{18}D_{18}CoF_{16}N_4O_{11}P_3Gd$, [M+H$^+$]: 1528. 11425; Found: 1528.11541. IR (KBr, cm^{-1}): 2292, 2077, 1665, 1625, 1591, 1505, 1465, 1385, 1354, 1324, 1276, 1237, 1171, 1144, 1115, 1065, 1045, 1025, 1002, 921, 846, 792, 746, 704, 660, 605, 579, 510, 477. UV/Vis (CH_2Cl_2, 25 °C): λ_{max} (log ε, M^{-1} cm^{-1}): 415(5.53), 545 (4.22).

Complex **[D$_{18}$]-5-Yb** Yield 85% (over two steps, based on the amount of porphyrin free base) ^{1}H NMR (400 MHz, $CDCl_3$) δ 17.32 (s, 4H), 10.99 (s, 4H), 9.07 (s, 4H), 8.75 (s, 4H), -5.19 (s, 5H). ^{19}F NMR (377 MHz, $CDCl_3$) δ -60.84 (s, 12F), -131.44 (s, 8F). ESI-HR-MS: calcd for $C_{59}H_{22}D_{18}CoF_{20}N_4O_9P_3Yb$, [M+H$^+$]: 1672.15392; Found: 1672.15009. IR (KBr, cm^{-1}): 2076, 1665, 1616, 1463, 1408, 1324, 1173, 1147, 1069, 1046, 1022, 843, 747, 704, 665, 580. UV/Vis (CH_2Cl_2, 25 °C): λ_{max} (log ε, M^{-1} cm^{-1}): 415(5.47), 544 (4.20), 585 (3.64).

Complex **[D$_{18}$]-5-Gd** Yield 82% (over two steps, based on the amount of porphyrin free base) ESI-HR-MS: calcd for $C_{59}H_{22}D_{18}CoF_{20}N_4O_9P_3Gd$, [M+H$^+$]: 1656.13916; Found: 1656.13545. IR (KBr, cm^{-1}): 2926, 2075, 1660, 1616, 1459, 1408, 1324, 1172, 1136, 1113, 1069, 1045, 1022, 1001, 844, 746, 703, 664, 579. UV/Vis (CH_2Cl_2, 25 °C): λ_{max} (log ε, M^{-1} cm^{-1}): 415(5.49), 545 (4.22), 585 (3.72).

2.3 Summary of Crystallographic Data

Table S1. Crystal data and structure refinement

Complex	**1-Yb**	**2-Yb**	**[D$_{18}$]-3-Yb**
molecular formula	$C_{55}H_{23}CoF_{28}N_4O_9P_3Yb$	$C_{55}H_{31}CoF_{20}N_4O_9P_3Yb$	$C_{55}H_{25}D_{18}CoF_8N_4O_9P_3Yb$
formula wt. (g mol^{-1})	1740.65	1596.71	1398.92
temperature (K)	180(2)	276.8(1)	180(2)
radiation (λ, Å)	0.71073	0.71073	0.71073
crystal system	monoclinic	orthorhombic	triclinic
space group	*C*2/*c*	*Cmcm*	$P\bar{1}$
a (Å)	24.588(5)	15.8901(4)	12.2948(3)
b (Å)	10.147(2)	19.2041(5)	14.0793(3)
c (Å)	24.582(5)	22.9477(6)	16.6204(4)
α (°)	90	90	72.8693(19)
β (°)	92.11(3)	90	73.873(2)
γ (°)	90	90	77.720(2)
Volume (Å^3)	6129(2)	7002.6(3)	2614.22(11)
Z	4	4	2
ρ_{calcd} (g cm^{-3})	1.886	1.739	1.777
μ (mm^{-1})	2.010	1.935	2.278
F(000)	3388	3596	1374
crystal size (mm^3)	0.20×0.18×0.11	0.21×0.17×0.15	0.25×0.14×0.08
Theta range	2.75, 27.48	3.817, 29.010	2.722, 27.452
reflections collected	50103	14681	39776
independent reflections	7019	3282	11924
Completeness	0.999	0.997	99.85
goodness-of-fit on F^2	1.052	1.111	1.047
final R indices [R > 2σ (I)]	0.0286, 0.0681	0.0501, 0.1492	0.0762, 0.1875
R indices (all data)	0.0335, 0.0719	0.0524, 0.1512	0.0885, 0.1999
largest diff. peak and hole (e Å^{-3})	1.207, -0.793	2.922, -1.773	12.480, -3.451

Table S2. Selected bond length for ytterbium complexes.

Complex	**1-Yb**	**2-Yb**	**[D$_{18}$]-3-Yb**
Yb-O / Å	2.205(4)	2.236(8)	2.220(7)
	2.236(4)	2.236(8)	2.232(6)
	2.224(4)	2.270(11)	2.245(7)
Yb-N / Å	2.380(2)	2.363(6)	2.369(7)
	2.376(2)	2.364(7)	2.359(6)
	2.380(2)	2.368(7)	2.375(6)
	2.376(2)	2.368(7)	2.378(6)
Yb-N4 plane / Å	1.180(1)	1.184(1)	1.169(1)

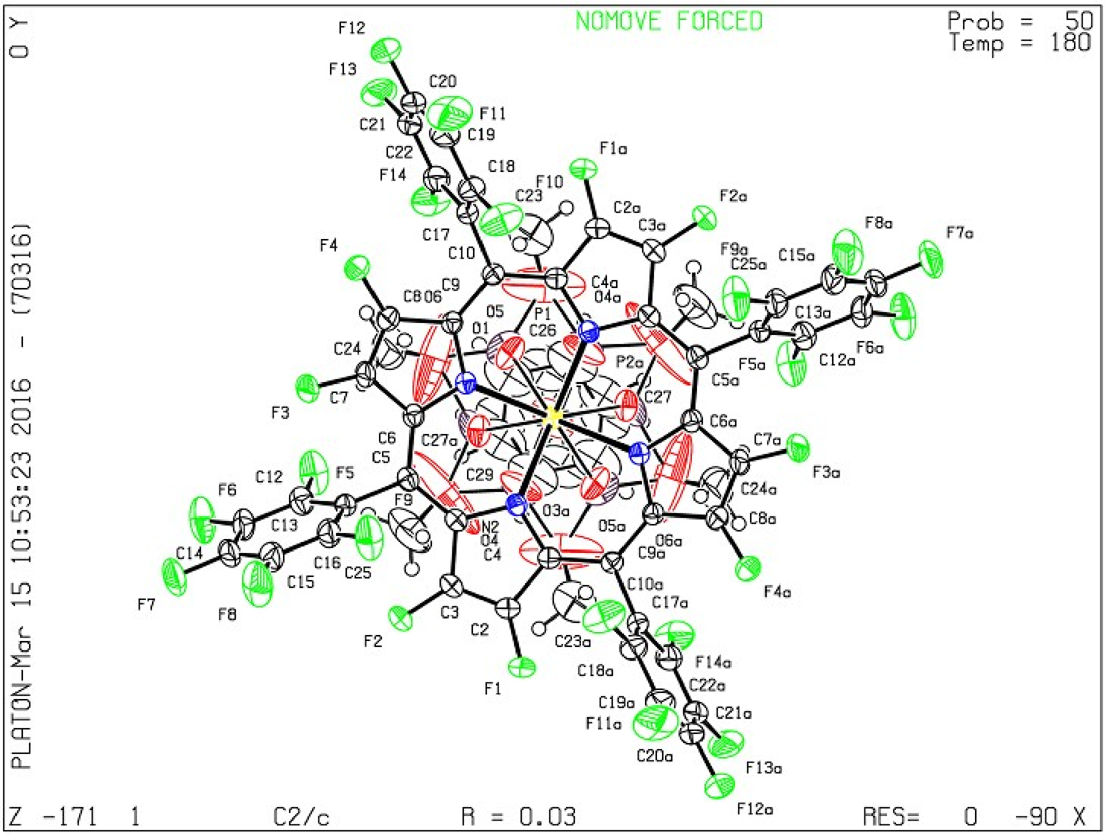

Figure SS2. Perspective drawing of **1-Yb** (CCDC 1501198) with 50% ellipsoids.

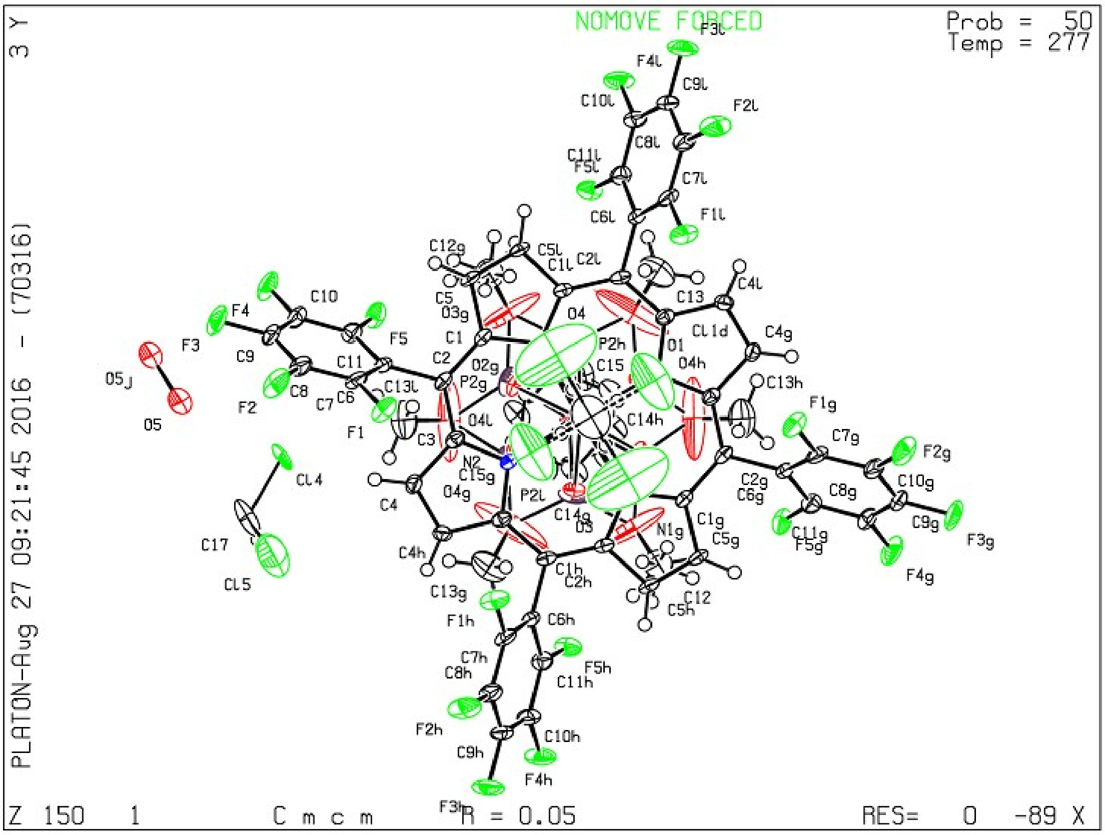

Figure S3. Perspective drawing of **2-Yb** (CCDC 1501199) with 50% ellipsoids.

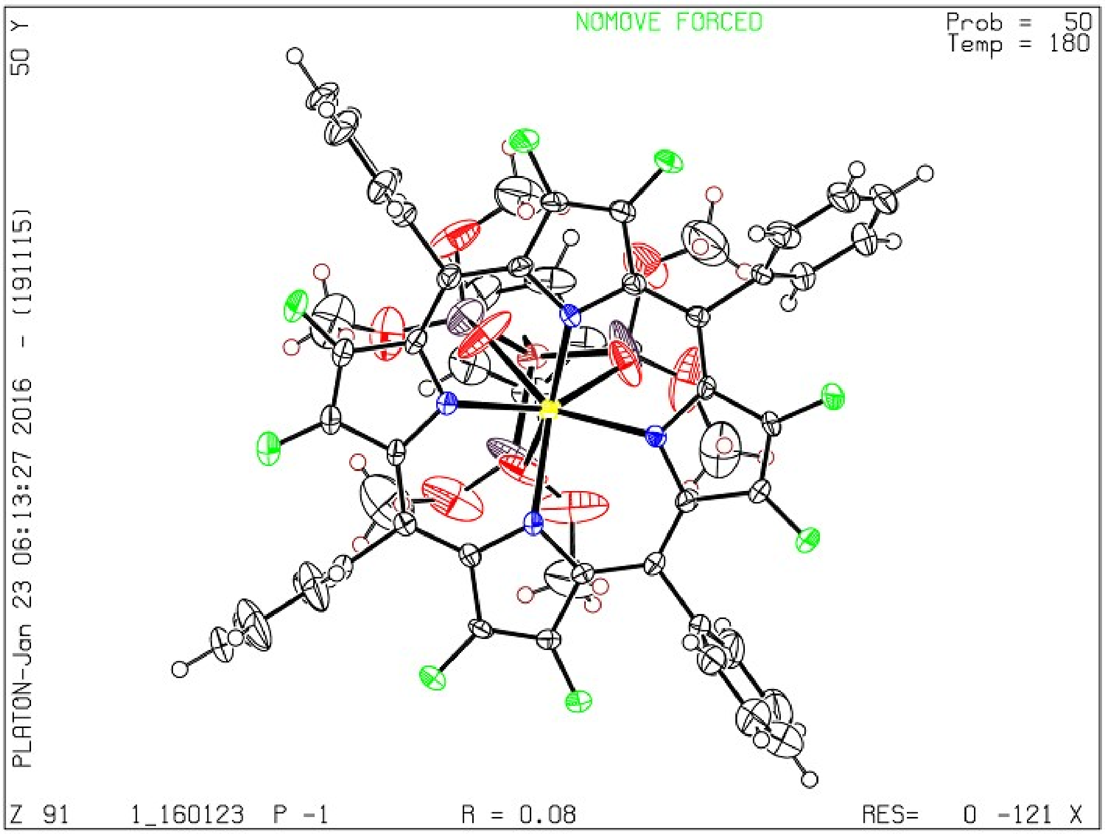

Figure S4. Perspective drawing of **[D$_{18}$]-3-Yb** (CCDC 1501200) with 50% ellipsoids.

2.4 NMR spectra

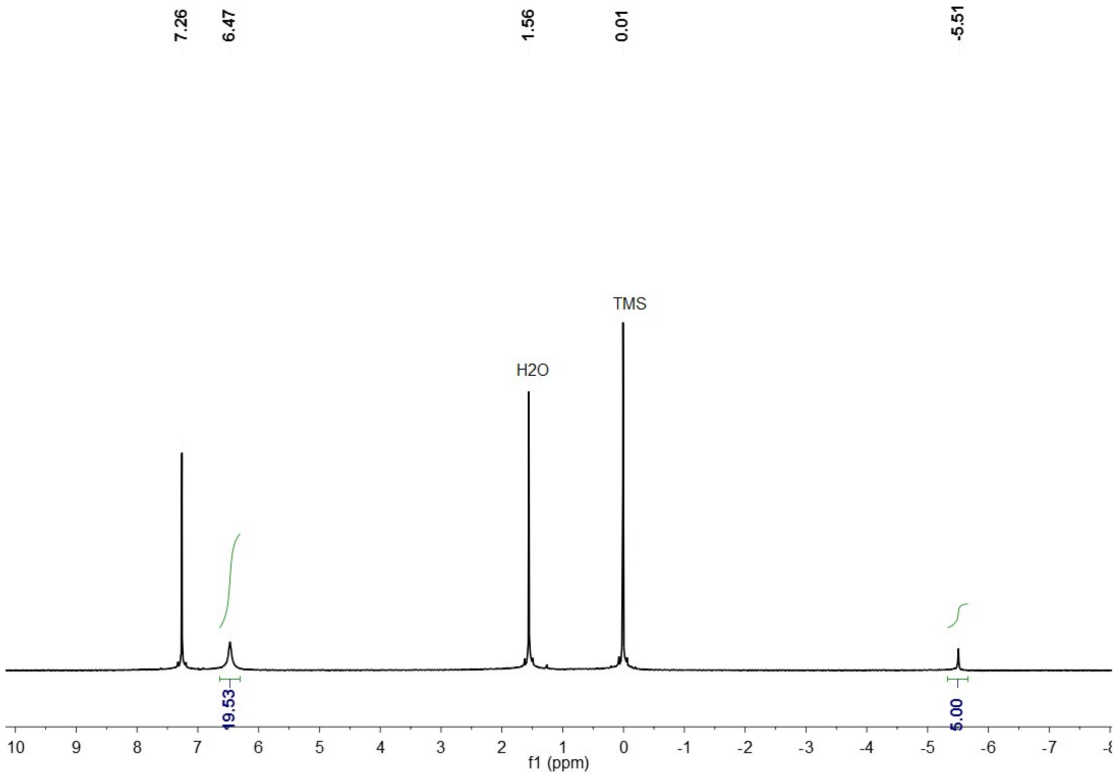

Figure S5. ^{1}H NMR ($CDCl_3$) of **1-Yb**

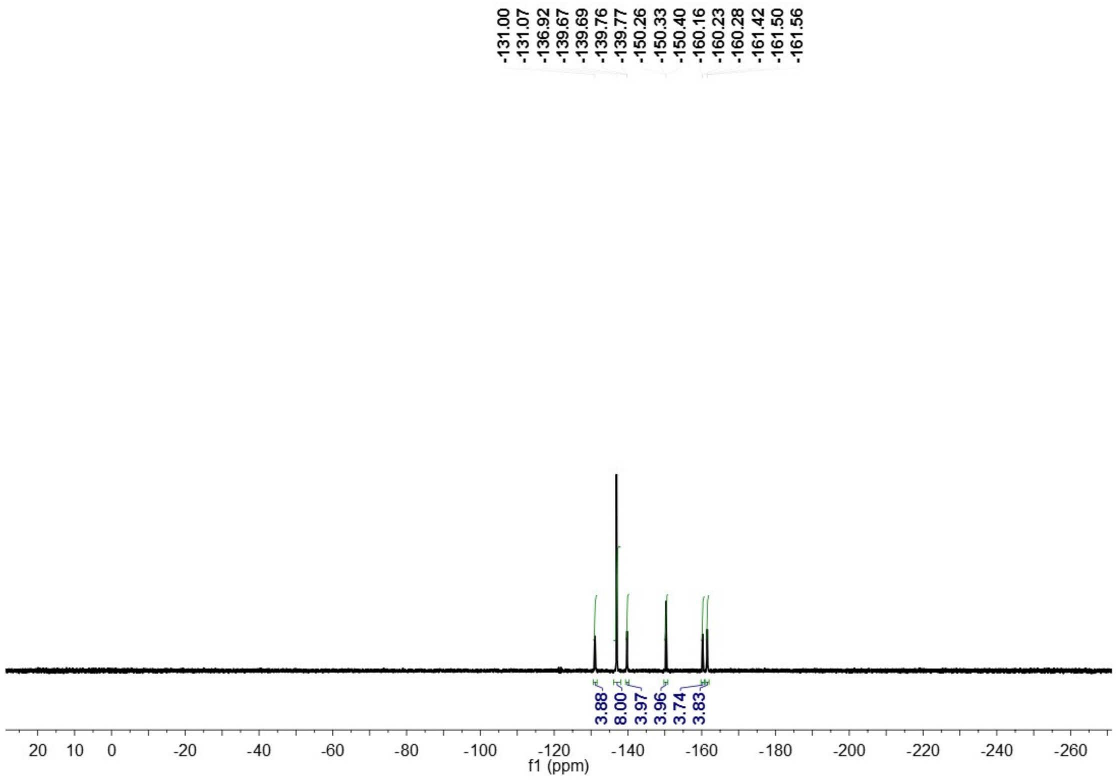

Figure S6. ^{19}F NMR ($CDCl_3$) of **1-Yb**

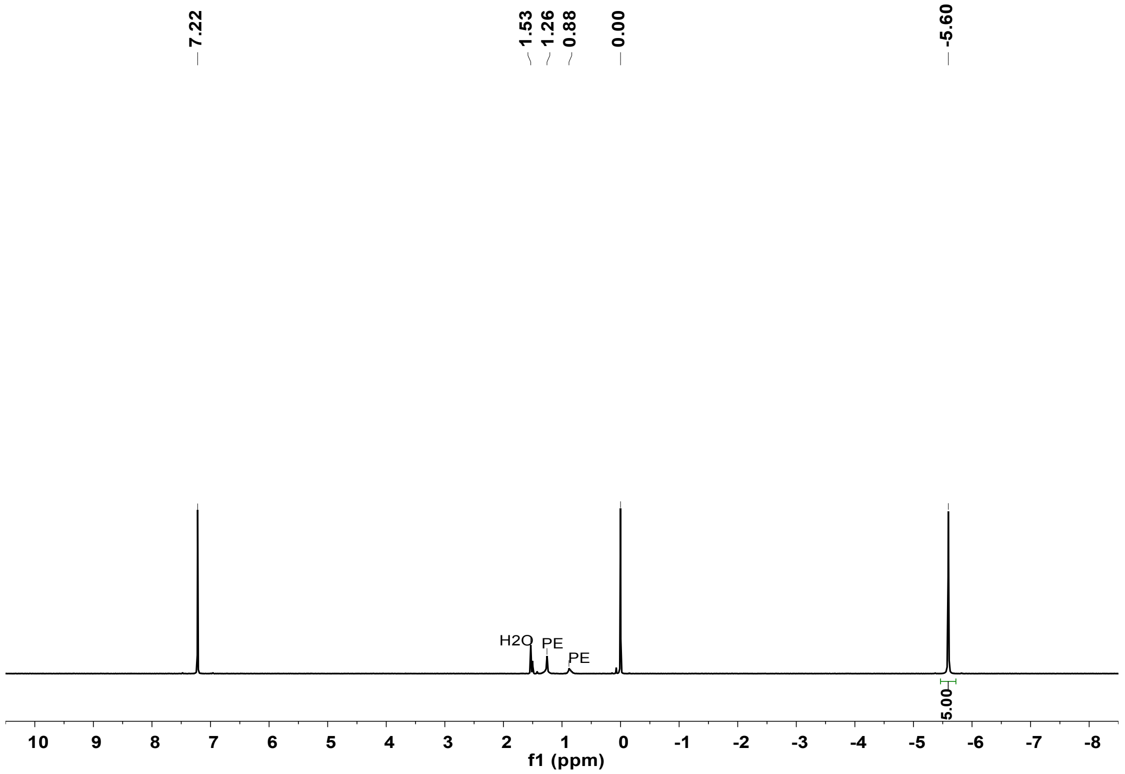

Figure S7. ^{1}H NMR ($CDCl_3$) of **[D$_{18}$]-1-Yb**

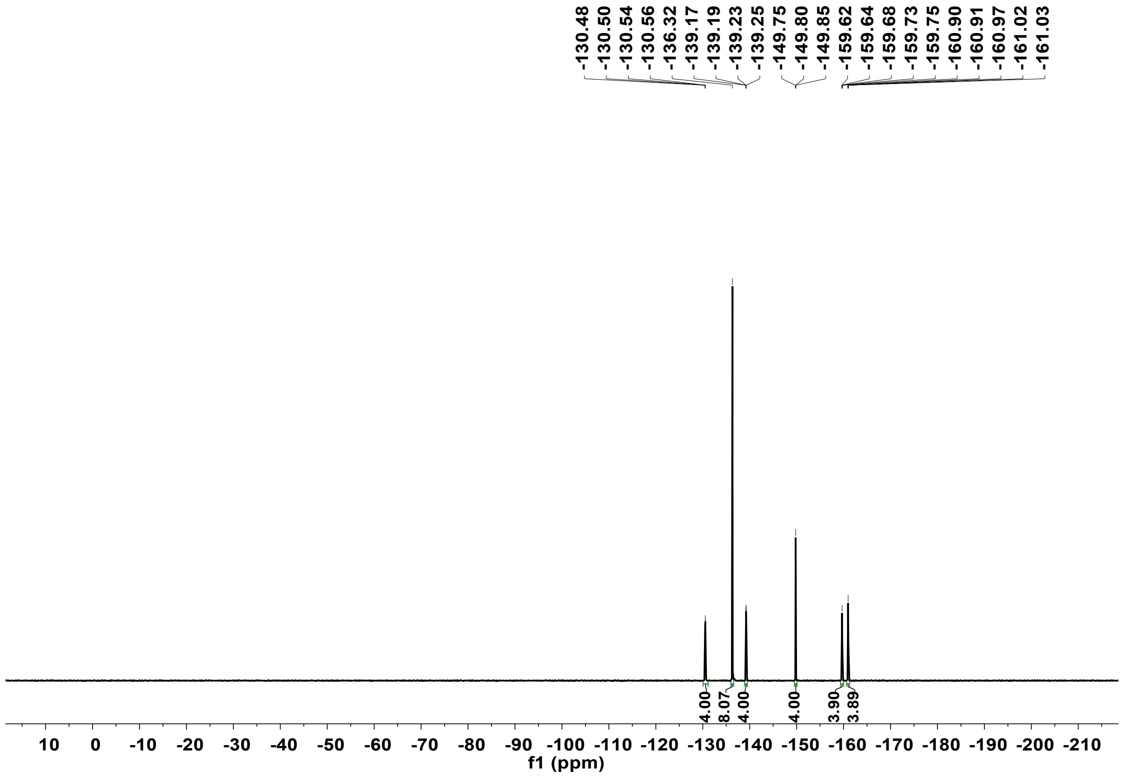

Figure S8. ^{19}F NMR ($CDCl_3$) of **[D$_{18}$]-1-Yb**

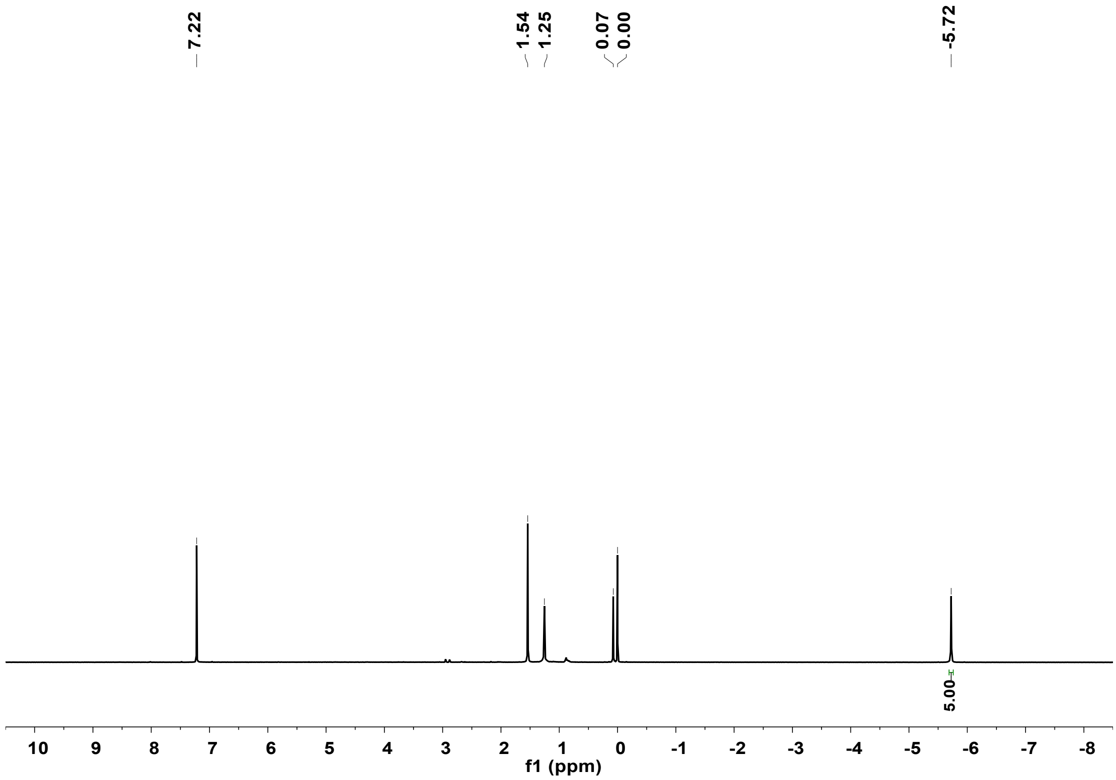

Figure S9. ^{1}H NMR ($CDCl_3$) of **[D$_{30}$]-1-Yb**

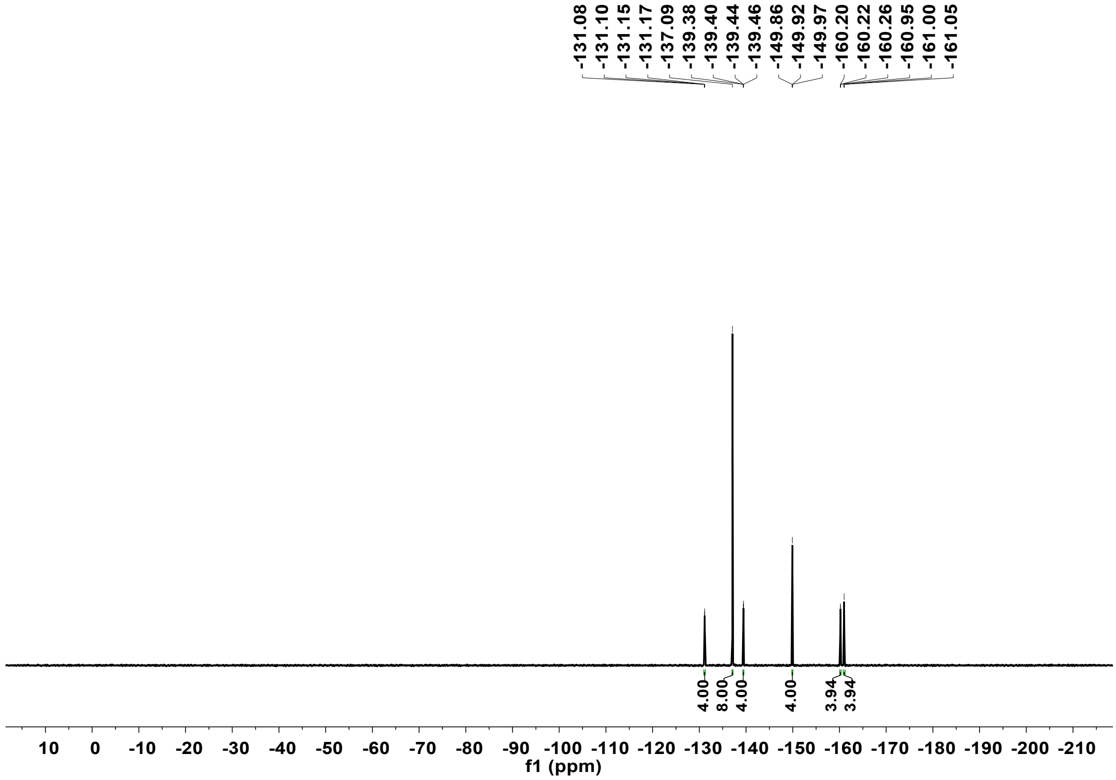

Figure S10. ^{19}F NMR ($CDCl_3$) of **[D$_{30}$]-1-Yb**

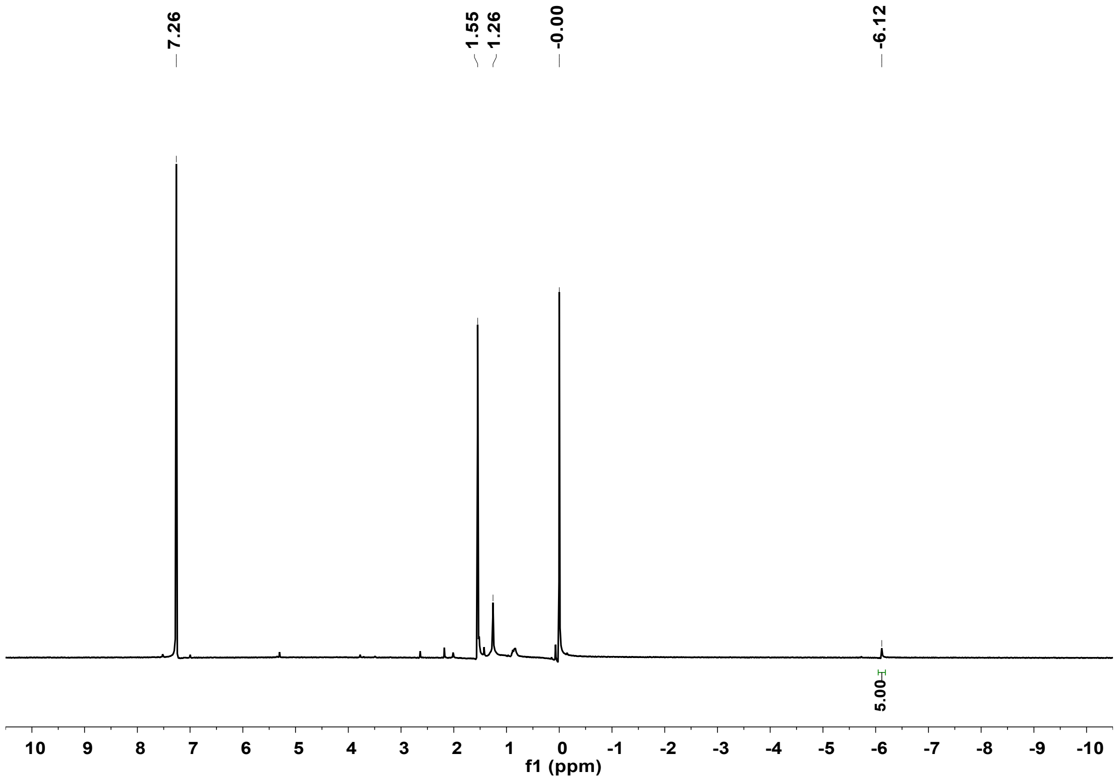

Figure S11. ^{1}H NMR ($CDCl_3$) of **[D$_{42}$]-1-Yb**

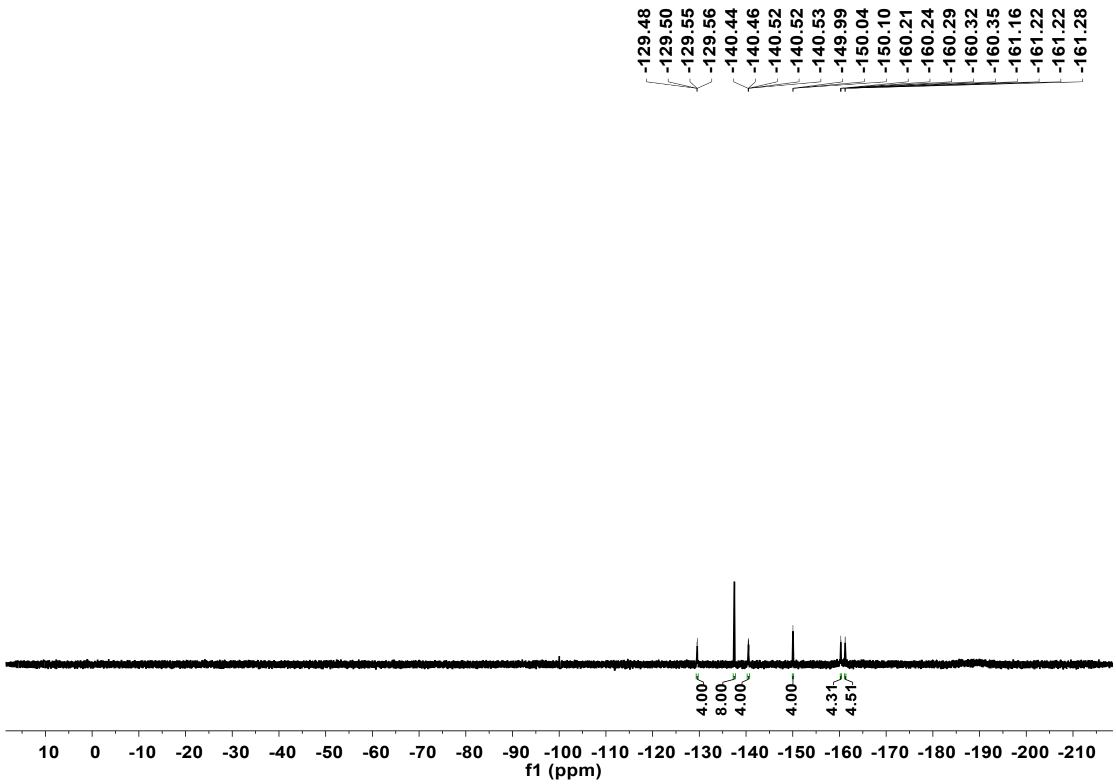

Figure S12. ^{19}F NMR ($CDCl_3$) of **[D$_{42}$]-1-Yb**

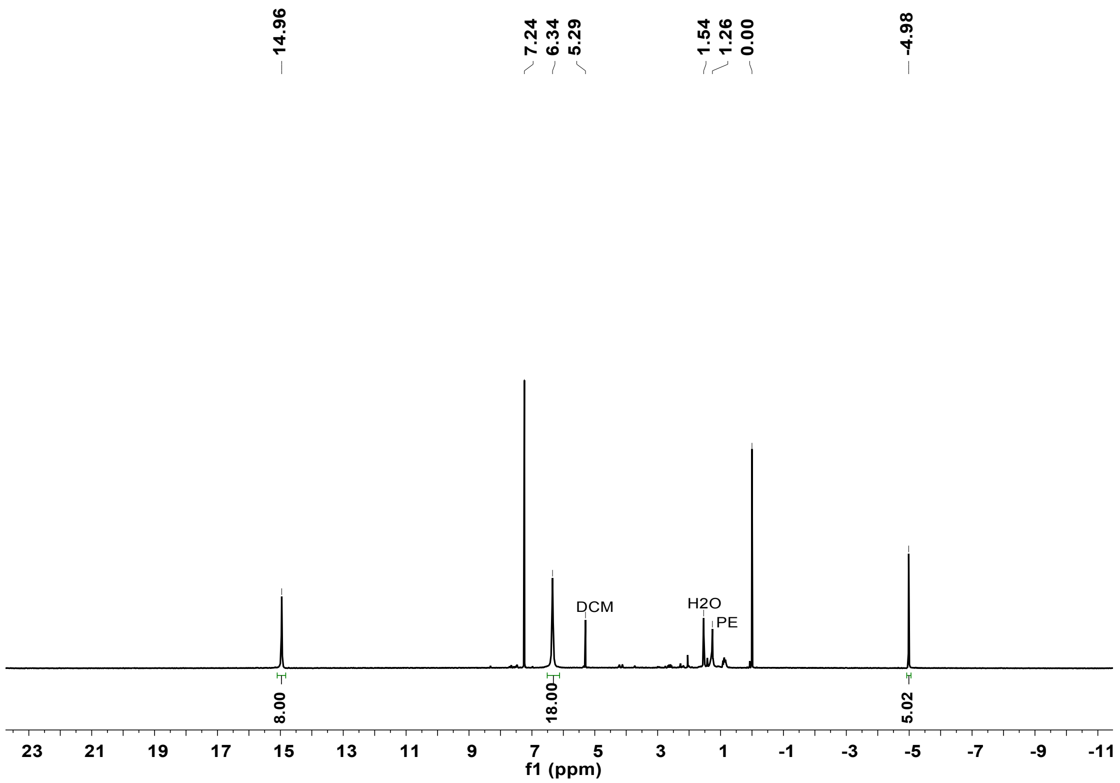

Figure S13. ^{1}H NMR ($CDCl_3$) of **2-Yb**

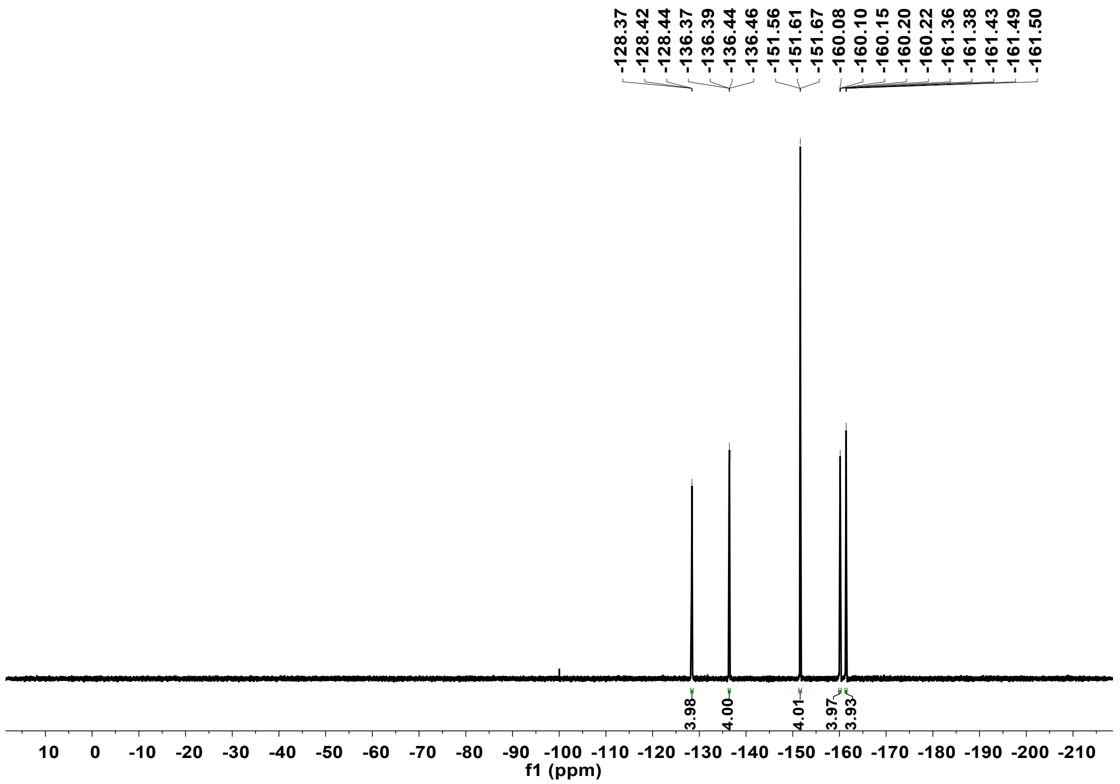

Figure S14.^{19}F NMR ($CDCl_3$) of **2-Yb**

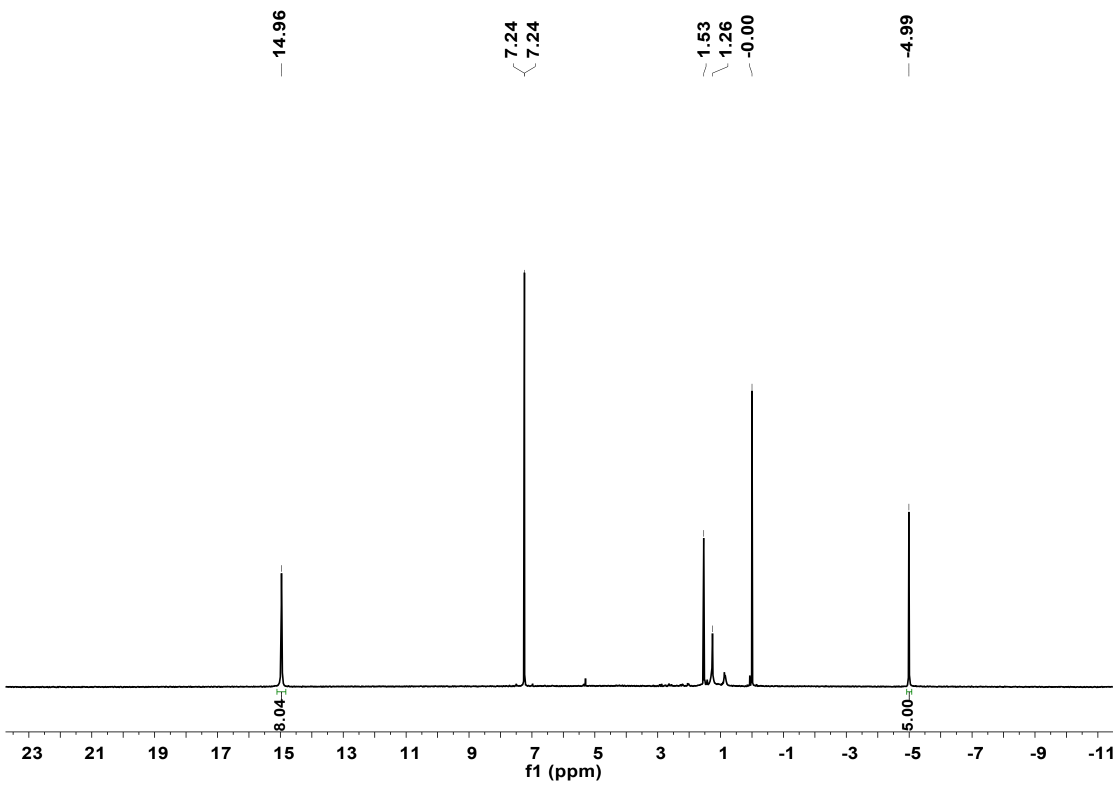

Figure S15. ^{1}H NMR ($CDCl_3$) of **[D$_{18}$]-2-Yb**

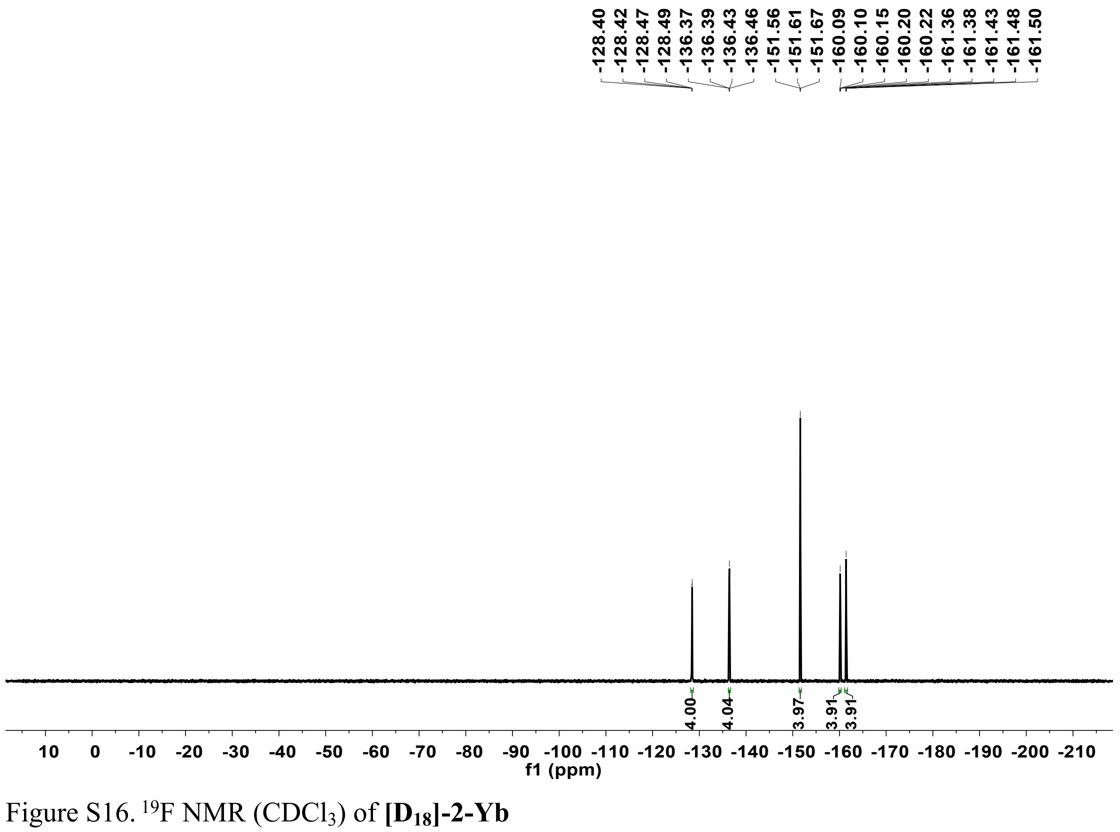

Figure S16. ^{19}F NMR ($CDCl_3$) of **[D$_{18}$]-2-Yb**

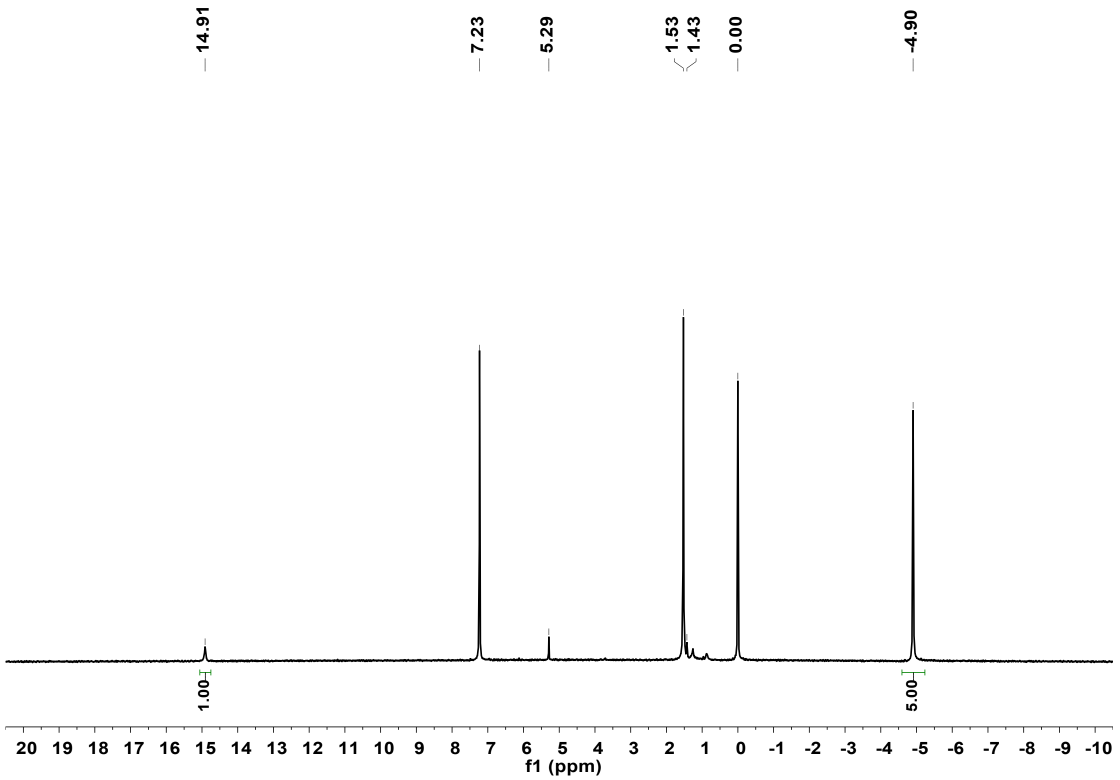

Figure S17. ^{1}H NMR ($CDCl_3$) of **[D$_{26}$]-2-Yb**

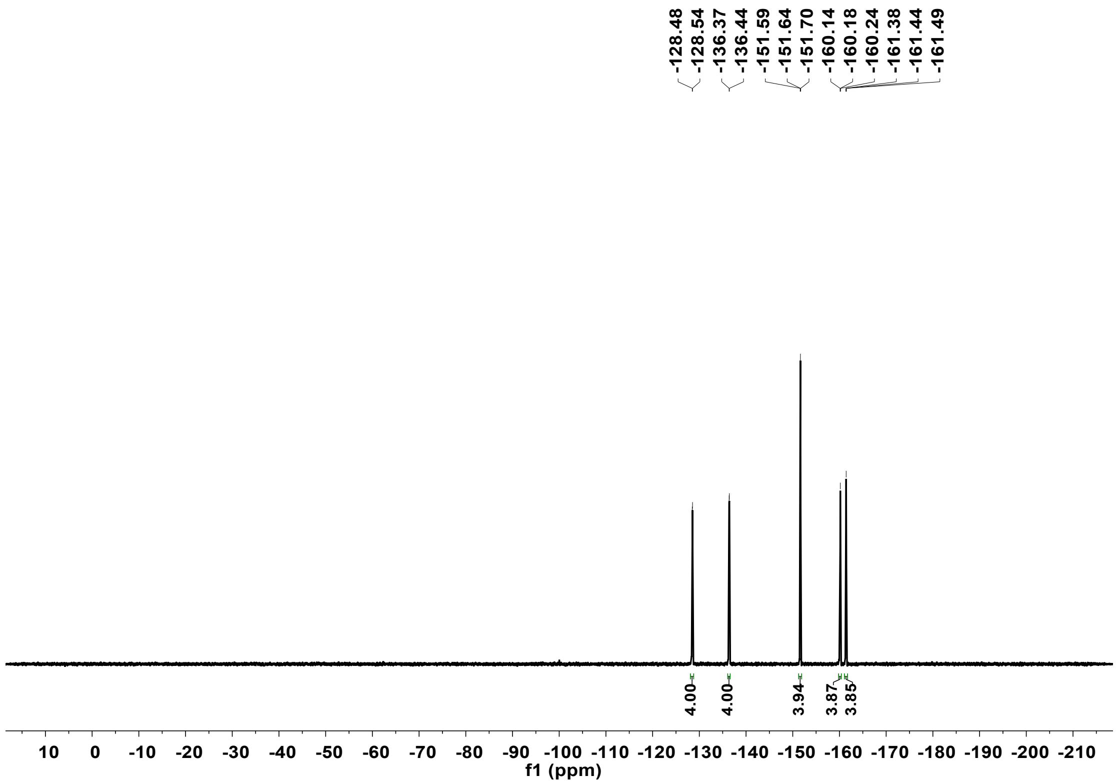

Figure S18. ^{19}F NMR ($CDCl_3$) of **[D$_{26}$]-2-Yb**

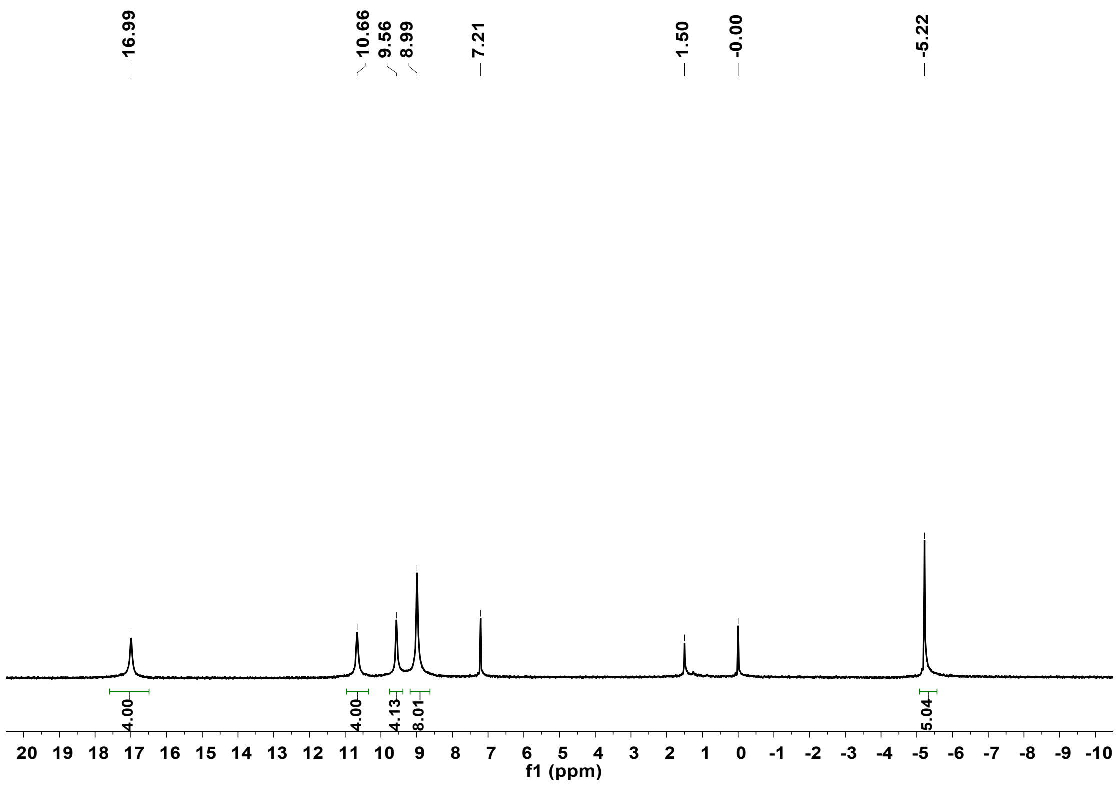

Figure S19. ^{1}H NMR ($CDCl_3$) of **[D$_{18}$]-3-Yb**

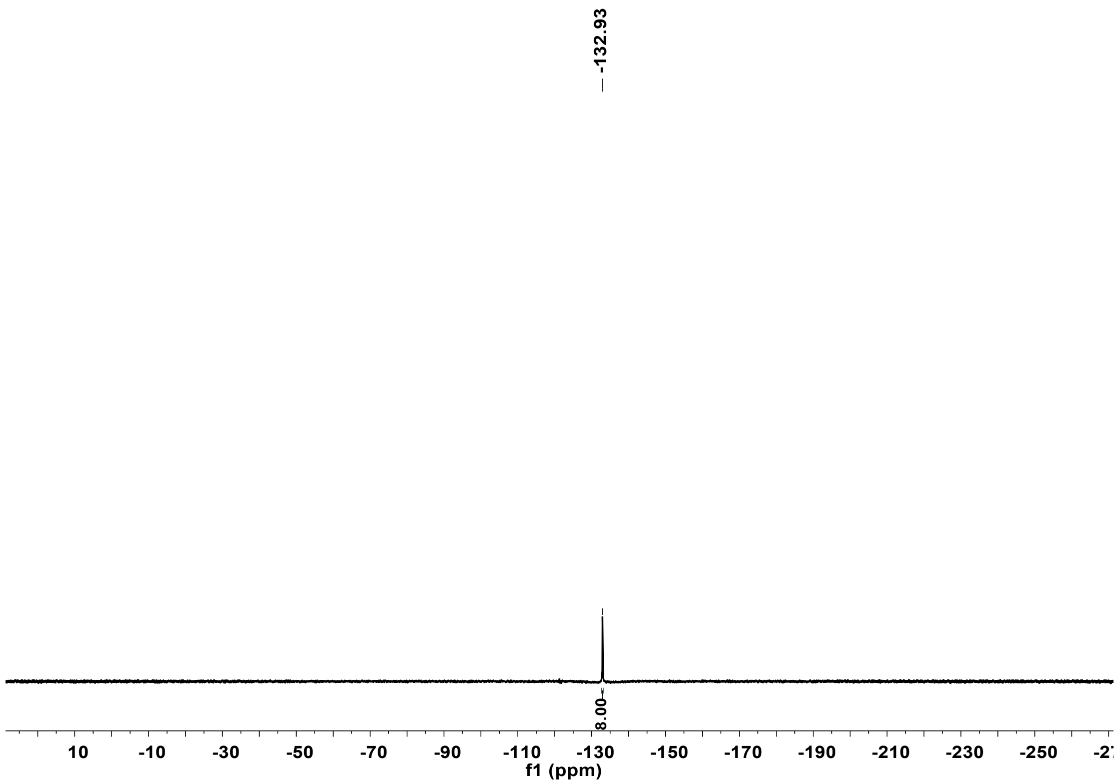

Figure S20. ^{19}F NMR ($CDCl_3$) of **[D$_{18}$]-3-Yb**

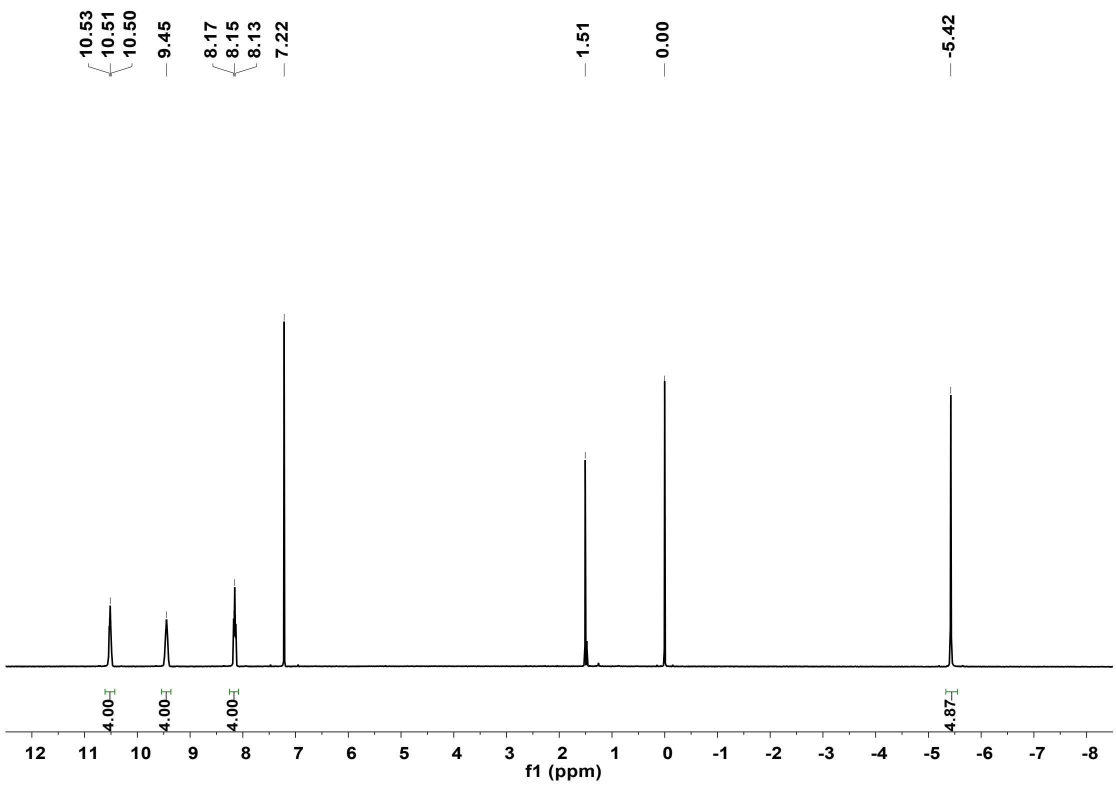

Figure S21. ^{1}H NMR ($CDCl_3$) of **[D$_{18}$]-4-Yb**

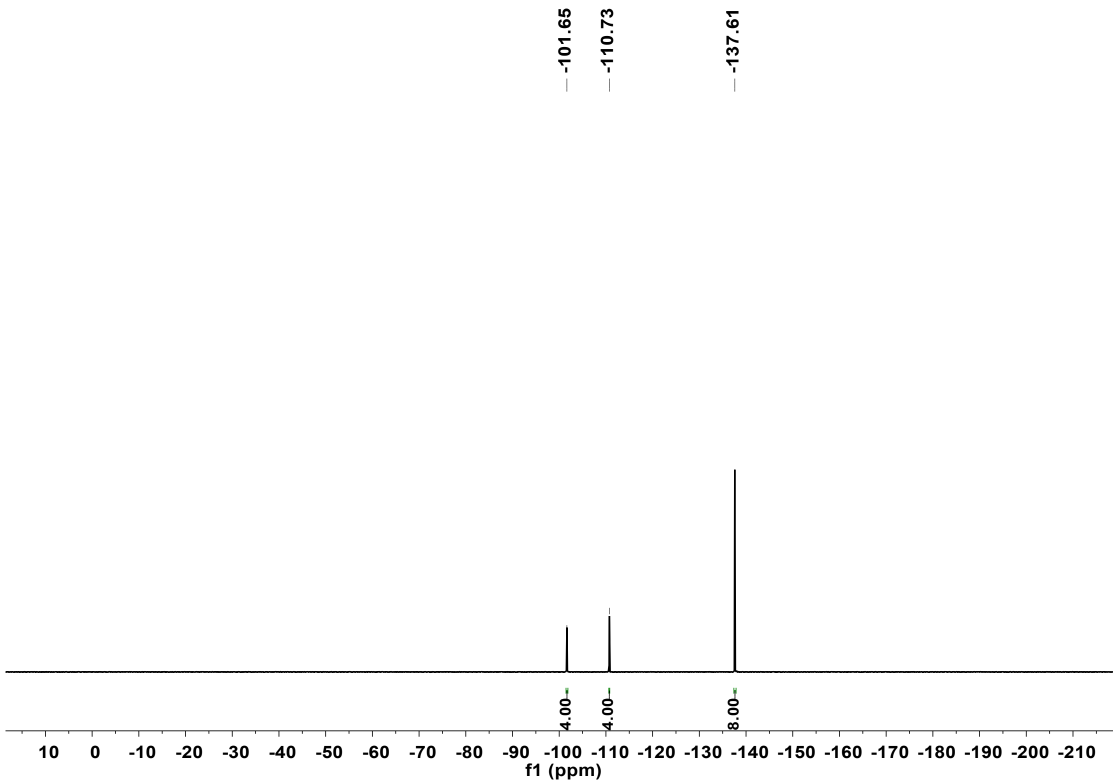

Figure S22. ^{19}F NMR ($CDCl_3$) of **[D$_{18}$]-4-Yb**

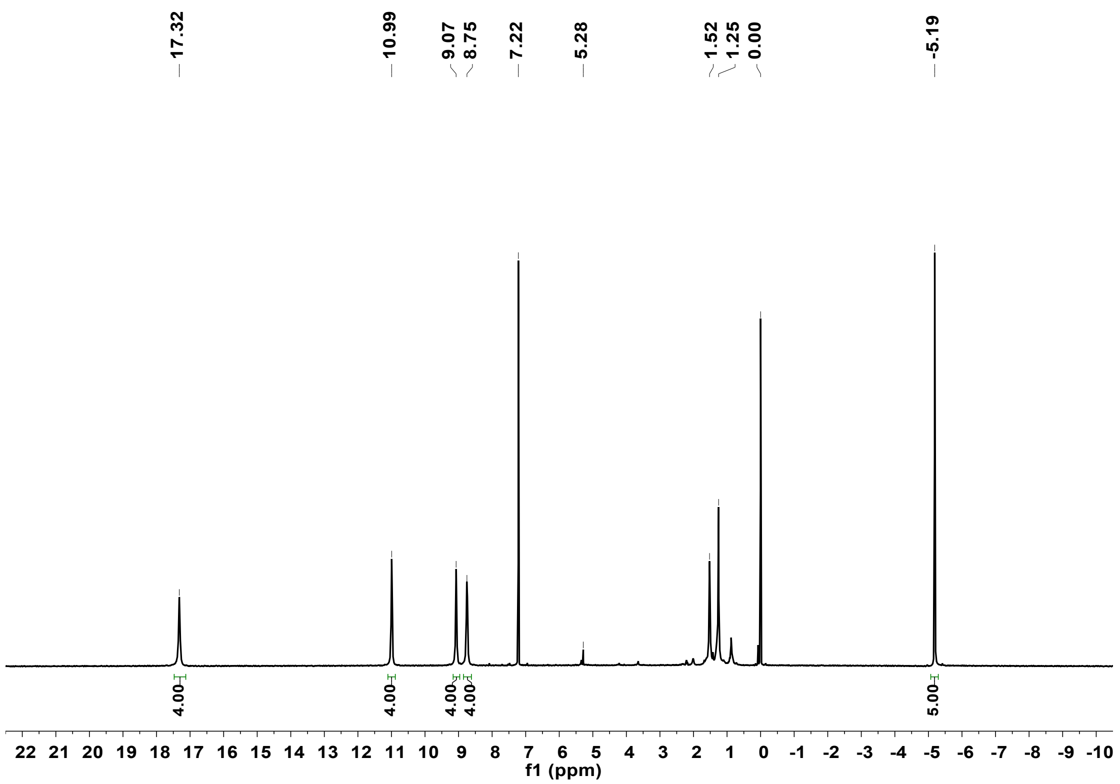

Figure S23. ^{1}H NMR ($CDCl_3$) of **[D$_{18}$]-5-Yb**

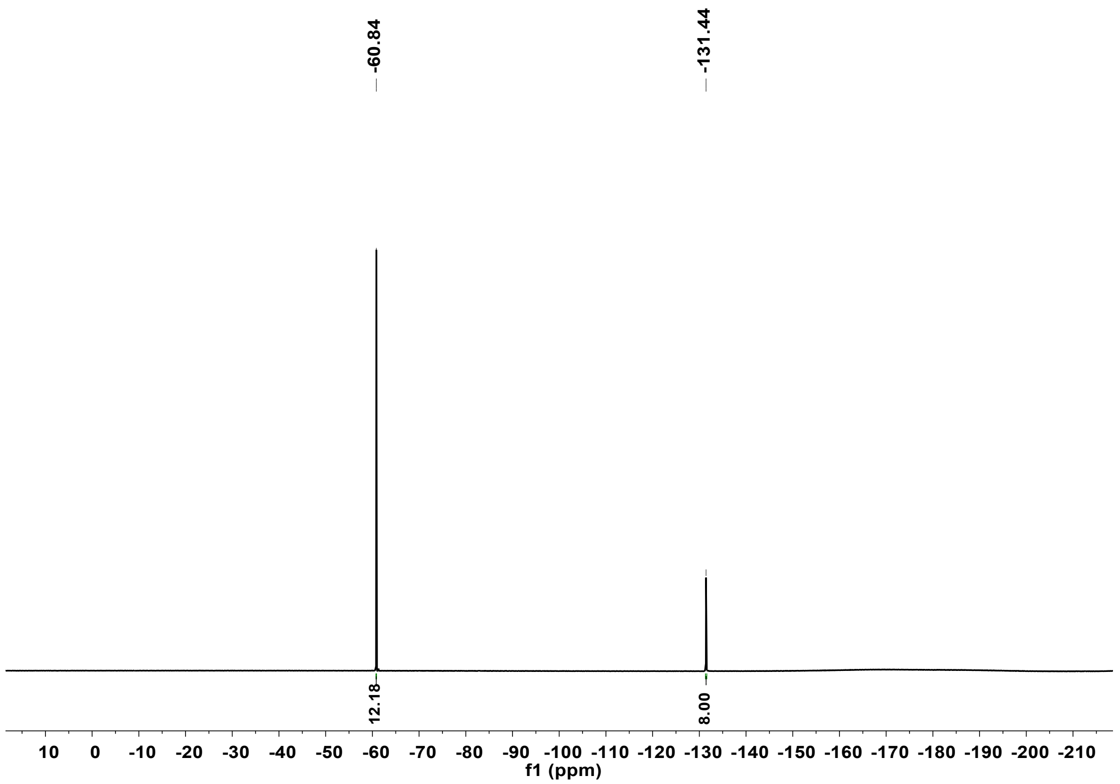

Figure S24. ^{19}F NMR ($CDCl_3$) of **[D$_{18}$]-5-Yb**

2.5 Experimental (up) and simulated (down) HR-ESI-MS spectra

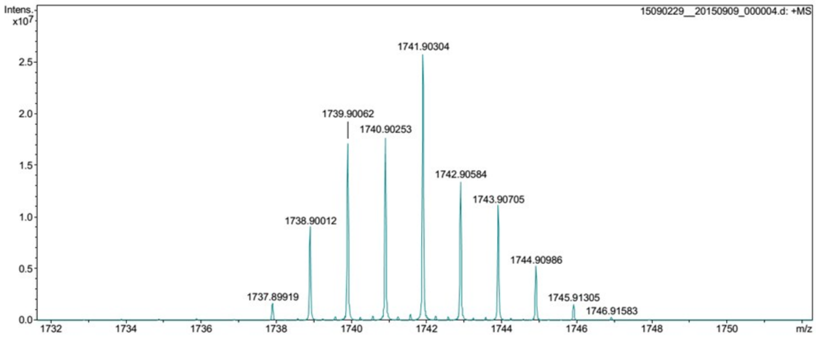

Meas. m/z	I	Formula	Score	m/z	err [mDa]	err [ppm]	mSigma	rdb	e⁻ Conf	N-Rule
1741.90304	25932226	C 55 H 24 Co F 28 N 4 O 9 P 3 Yb	100.00	1741.90339	0.4	0.2	4.8	33.5	even	-

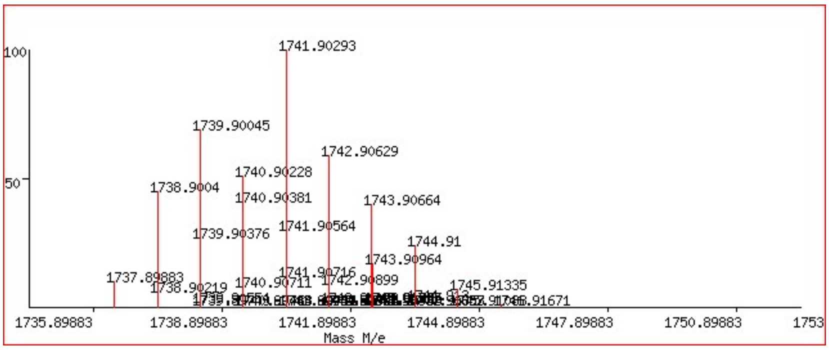

Figure S25. HR-ESI-MS spectrum of **1-Yb**

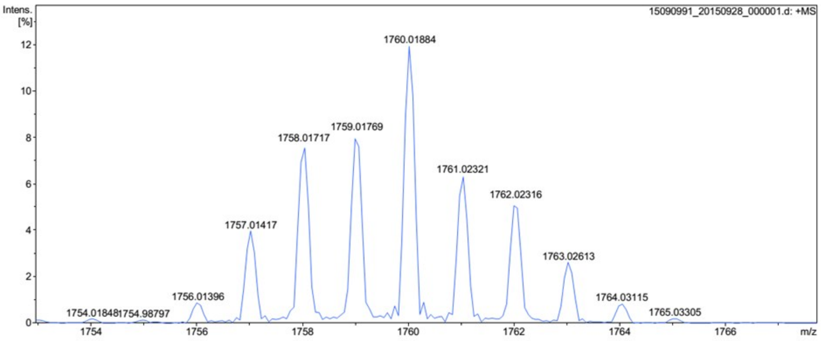

Meas. m/z	I	Formula	Score	m/z	err [mDa]	err [ppm]	mSigma	rdb	e⁻ Conf	N-Rule
1760.01884	12629804	C 55 H 6 Co D 18 F 28 N 4 O 9 P 3 Yb	100.00	1760.01637	-2.5	-1.4	13.3	33.5	even	-

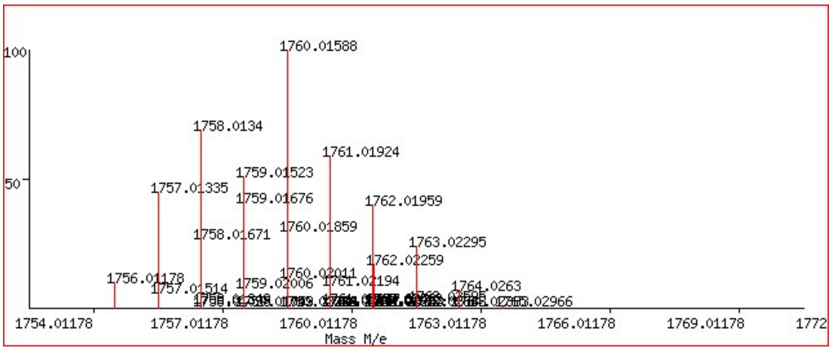

Figure S26. HR-ESI-MS spectrum of **[D$_{18}$]-1-Yb**

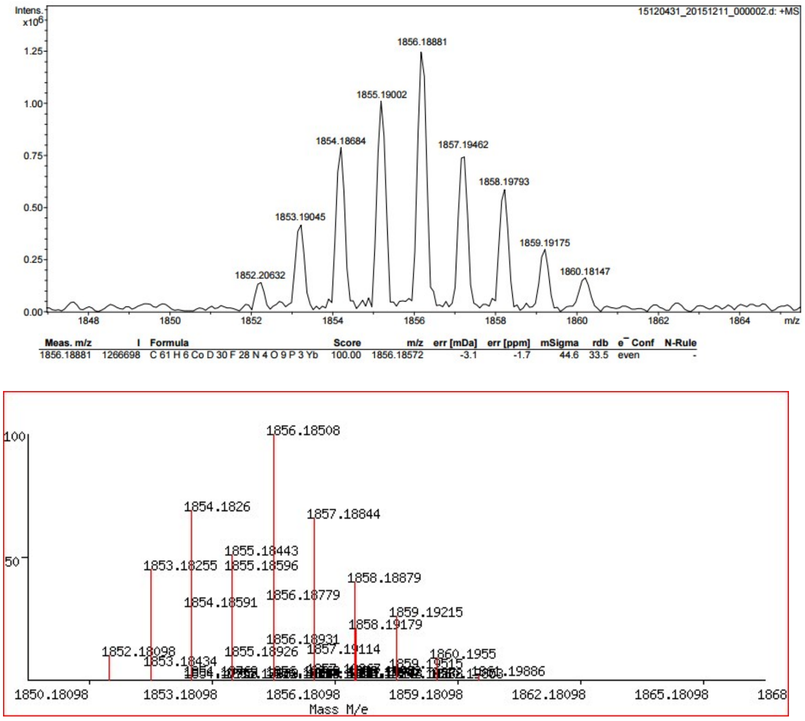

Figure S27. HR-ESI-MS spectrum of **[D$_{30}$]-1-Yb**

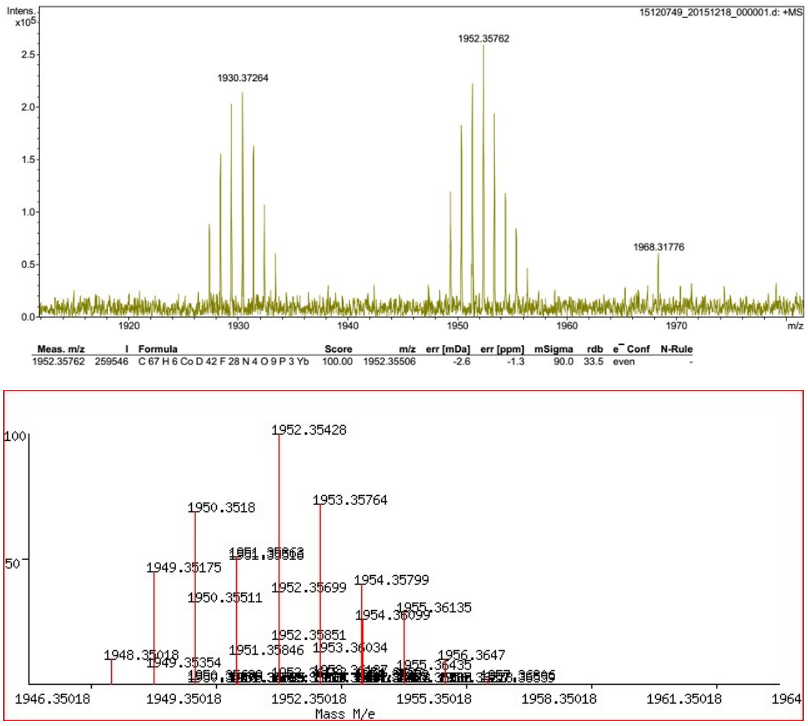

Figure S28. HR-ESI-MS spectrum of **[D$_{42}$]-1-Yb**

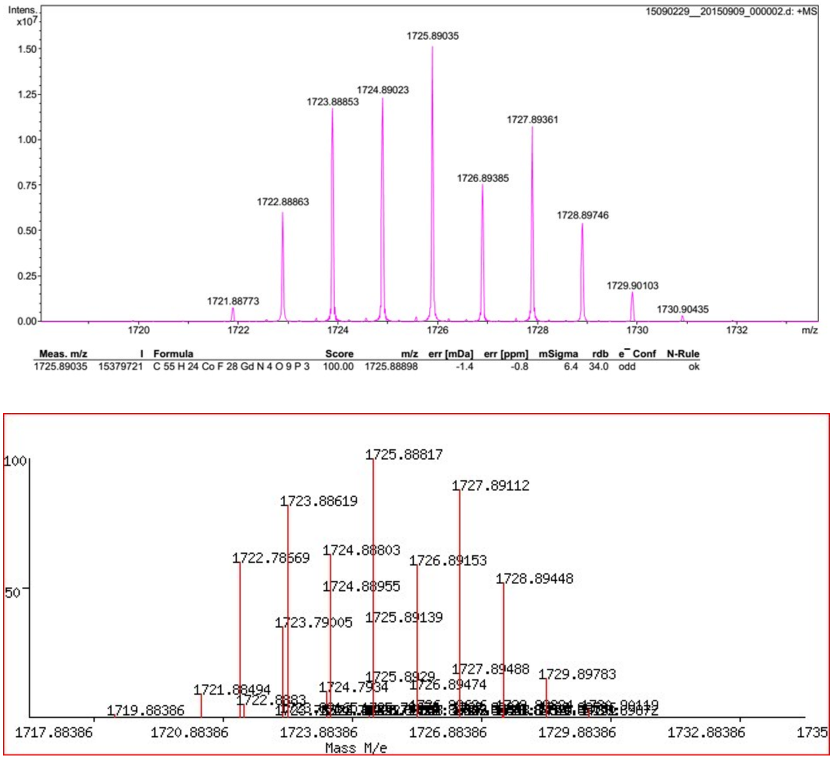

Figure S29. HR-ESI-MS spectrum **1-Gd**

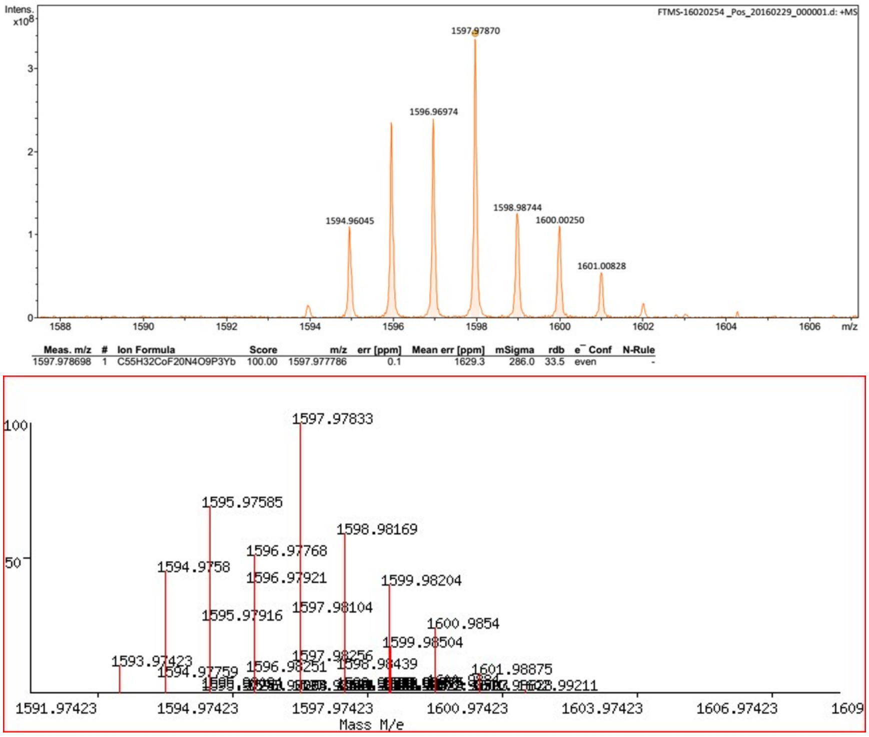

Figure S30. HR-ESI-MS spectrum of **2-Yb**

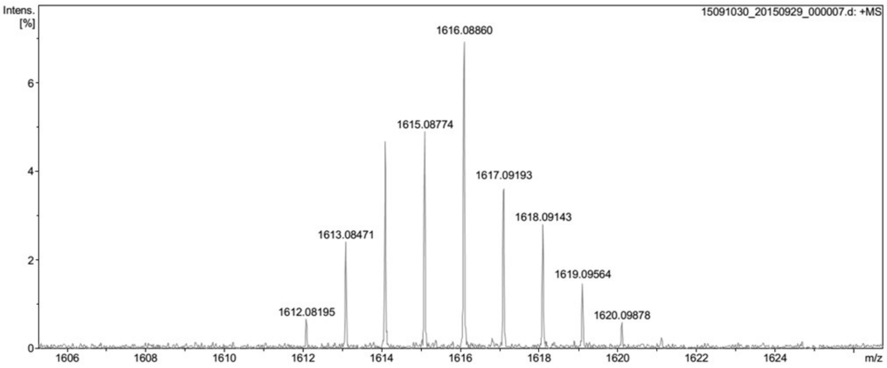

Meas. m/z	I	Formula	Score	m/z	err [mDa]	err [ppm]	mSigma	rdb	e⁻ Conf	N-Rule
1616.08860	1010011	C 55 H 14 Co D 18 F 20 N 4 O 9 P 3 Yb	100.00	1616.09175	3.2	2.0	17.9	33.5	even	-

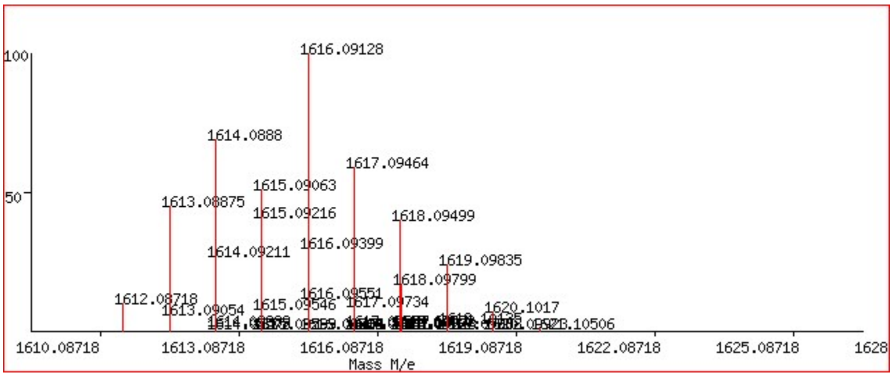

Figure S31. HR-ESI-MS spectrum of **[D$_{18}$]-2-Yb**

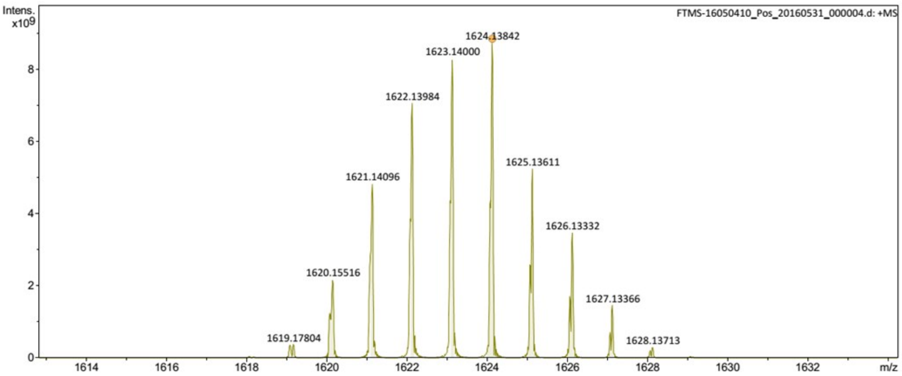

Meas. m/z	#	Ion Formula	Score	m/z	err [ppm]	Mean err [ppm]	mSigma	rdb	e⁻ Conf	N-Rule
1624.138418	1	C55H6CoD26F20N4O9P3Yb	100.00	1624.140982	2.2	1032.6	137.5	33.5	even	-

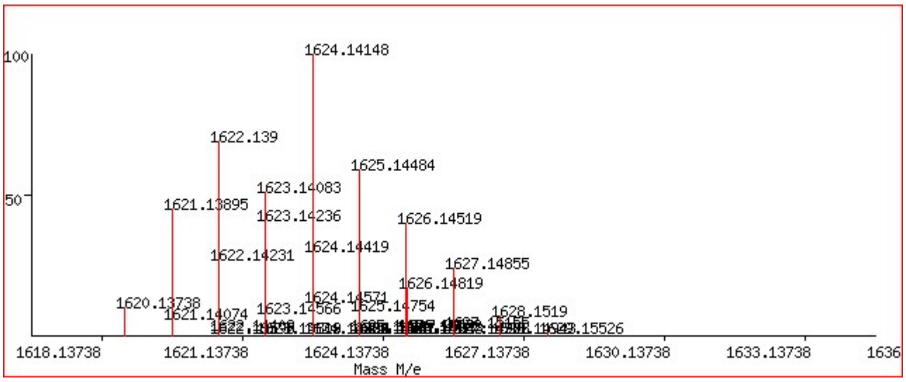

Figure S32. HR-ESI-MS spectrum of **[D$_{26}$]-2-Yb**

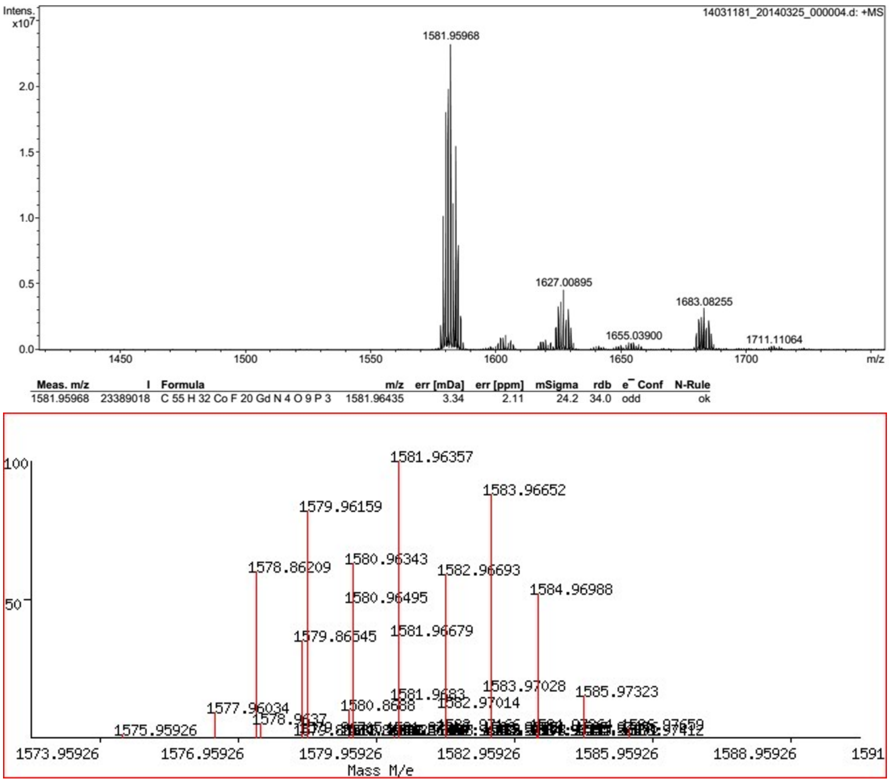

Meas. m/z	I	Formula	m/z	err [mDa]	err [ppm]	mSigma	rdb	e⁻ Conf	N-Rule
1581.95968	23389018	C 55 H 32 Co F 20 Gd N 4 O 9 P 3	1581.96435	3.34	2.11	24.2	34.0	odd	ok

Figure S33. HR-ESI-MS spectrum of **2-Gd**

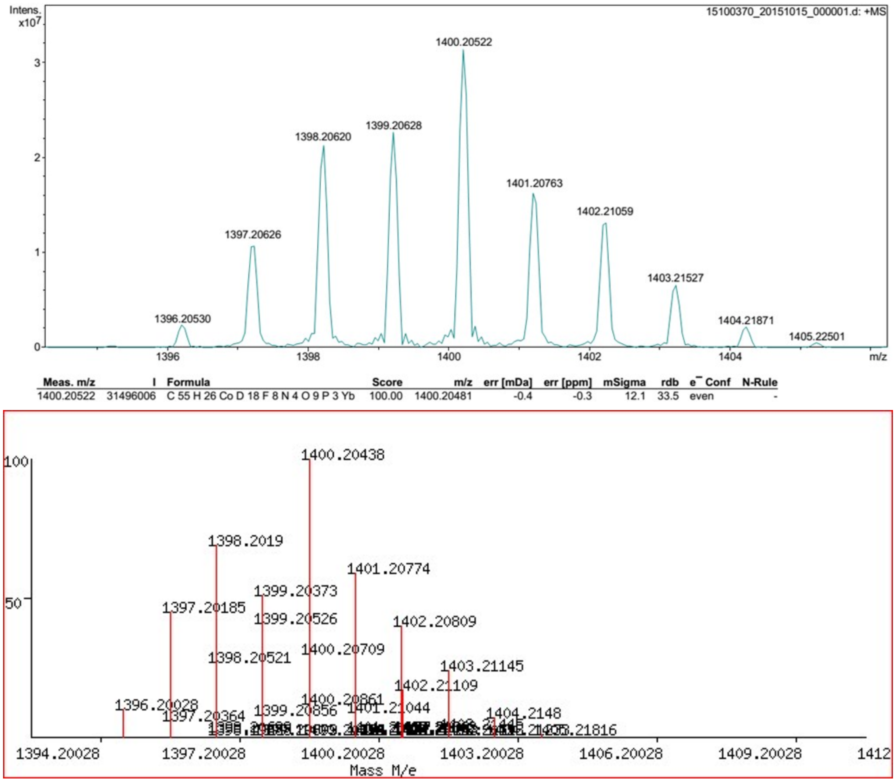

Meas. m/z	I	Formula	Score	m/z	err [mDa]	err [ppm]	mSigma	rdb	e⁻ Conf	N-Rule
1400.20522	31496006	C 55 H 26 Co D 18 F 8 N 4 O 9 P 3 Yb	100.00	1400.20481	-0.4	-0.3	12.1	33.5	even	-

Figure S34. HR-ESI-MS spectrum of **[D$_{18}$]-3-Yb**

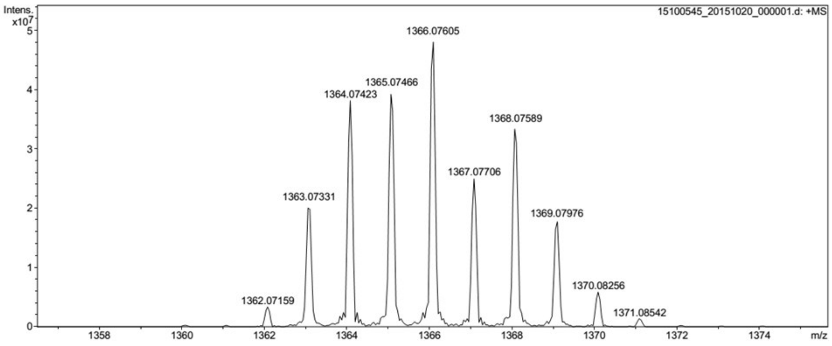

Meas. m/z	I	Formula	Score	m/z	err [mDa]	err [ppm]	mSigma	rdb	e⁻ Conf	N-Rule
1366.07605	48841052	C 55 H 44 Co F 8 Gd N 4 O 9 P 3	100.00	1366.07741	1.4	1.0	12.7	34.0	odd	ok

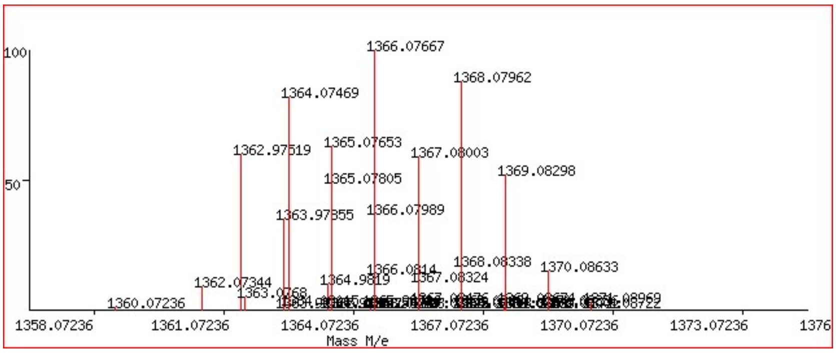

Figure S35. HR-ESI-MS spectrum of **3-Gd**

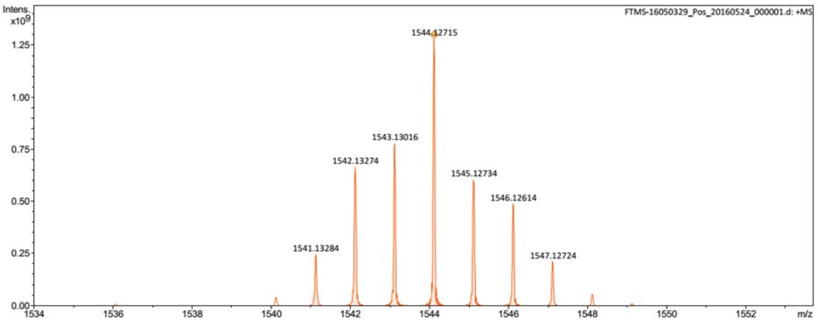

Meas. m/z	#	Ion Formula	Score	m/z	err [ppm]	Mean err [ppm]	mSigma	rdb	e⁻ Conf	N-Rule
1544.127150	1	C55H18CoD18F16N4O9P3Yb	100.00	1544.128455	1.5	730.1	80.2	33.5	even	-

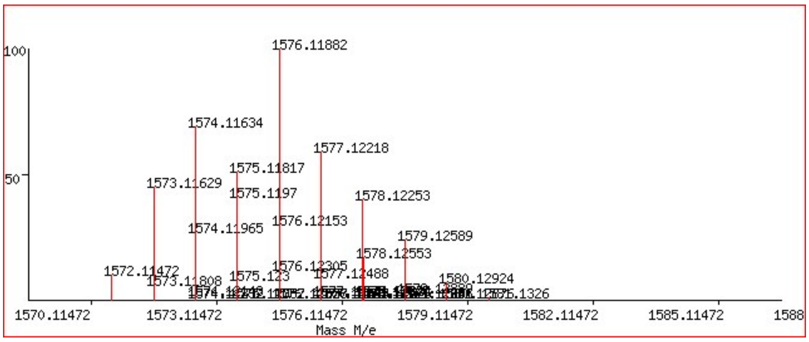

Figure S36. HR-ESI-MS spectrum of **[D$_{18}$]-4-Yb**

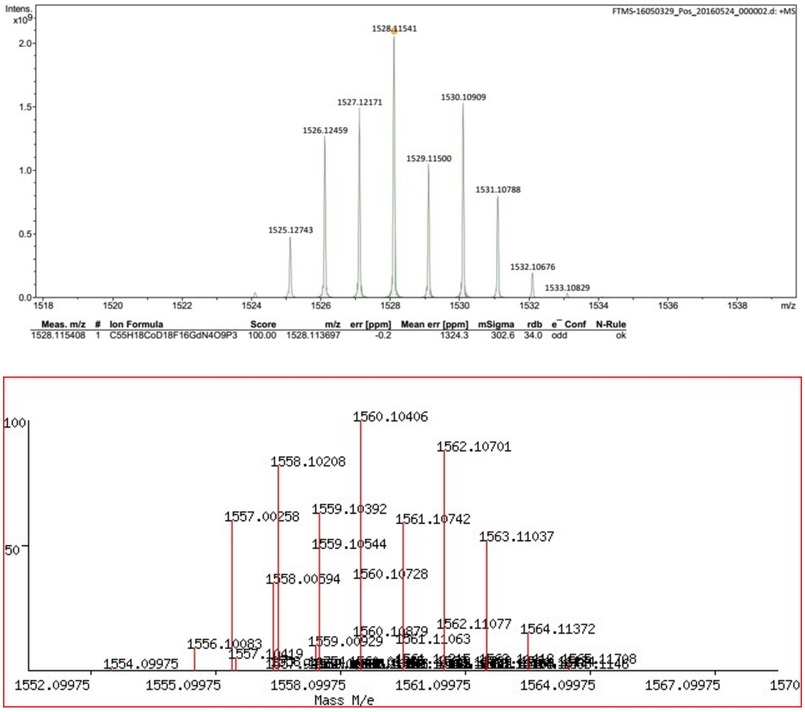

Figure S37. HR-ESI-MS spectrum of **[D$_{18}$]-4-Gd**

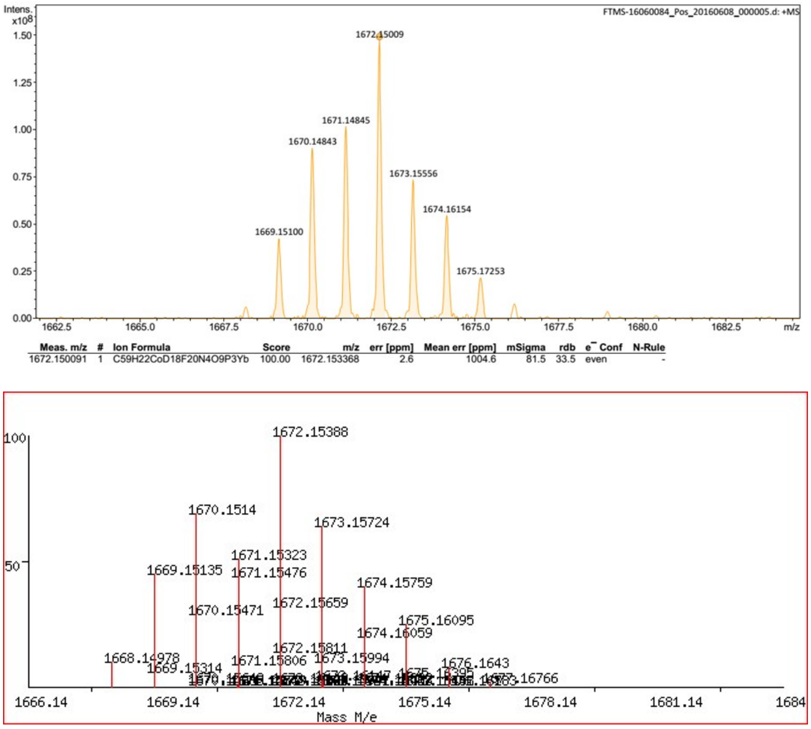

Figure S38. HR-ESI-MS spectrum of **[D$_{18}$]-5-Yb**

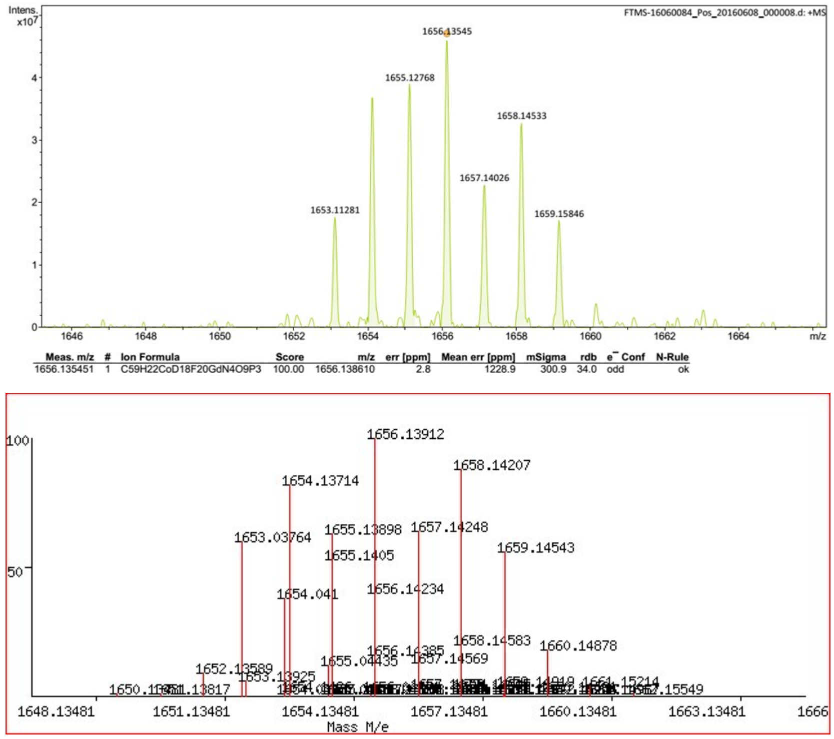

Figure S39. HR-ESI-MS spectrum of **[D$_{18}$]-5-Gd**

2.6 IR spectra.

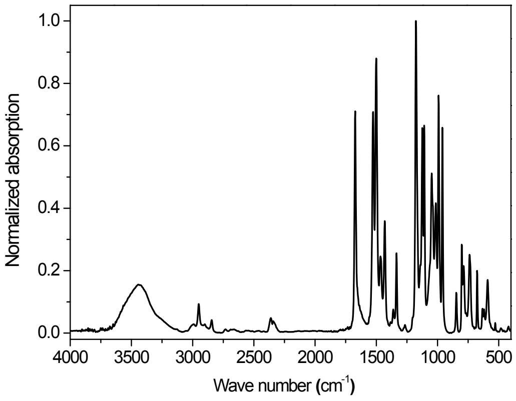

Figure S40. IR spectrum of **1-Yb**

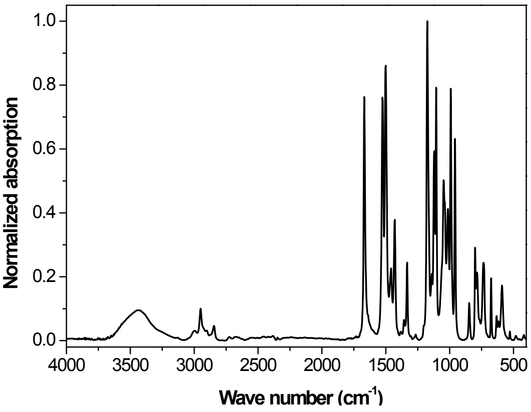

Figure S41. IR spectrum of **[D$_{18}$]-1-Yb**

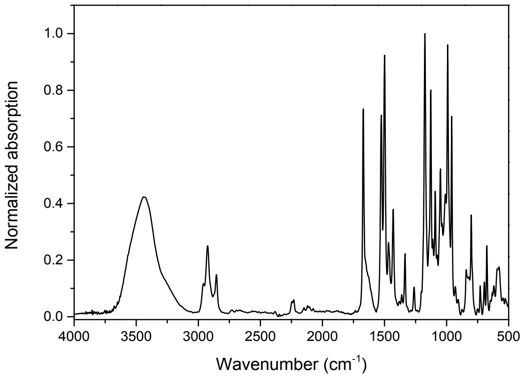

Figure S42. IR spectrum of **[D$_{30}$]-1-Yb**

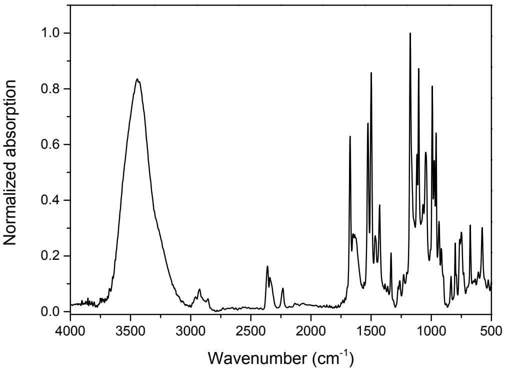

Figure S43. IR spectrum of **[D$_{42}$]-1-Yb**

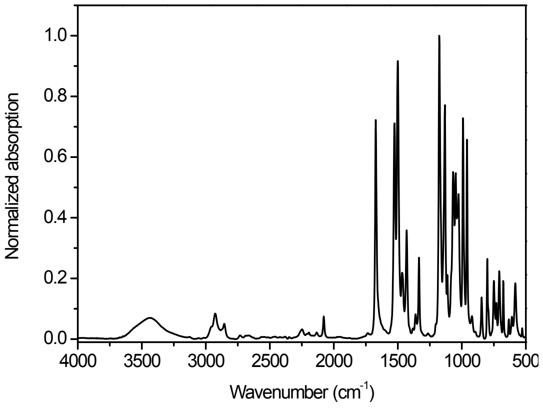

Figure S44. IR spectrum of **1-Gd**

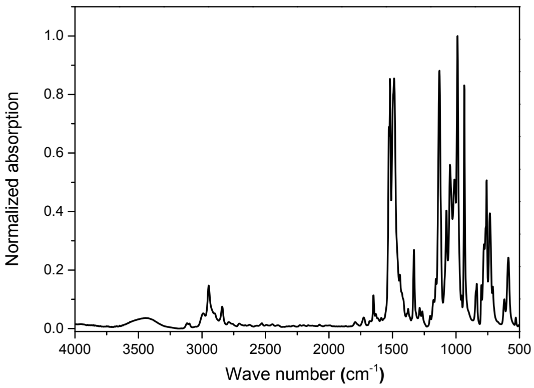

Figure S45. IR spectrum of **2-Yb**

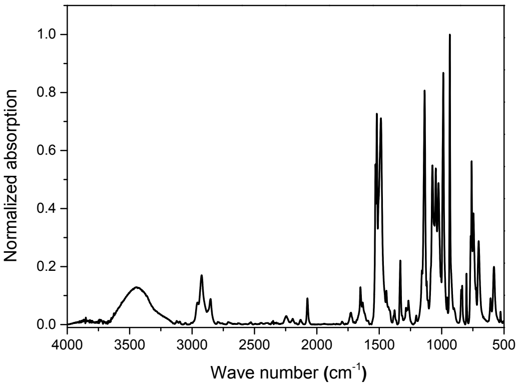

Figure S46. IR spectrum of **[D18]-2-Yb**

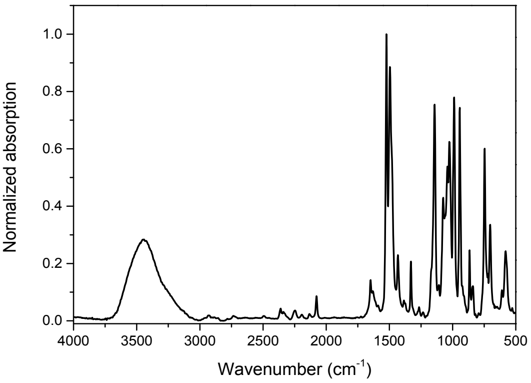

Figure S47. IR spectrum of **[D26]-2-Yb**

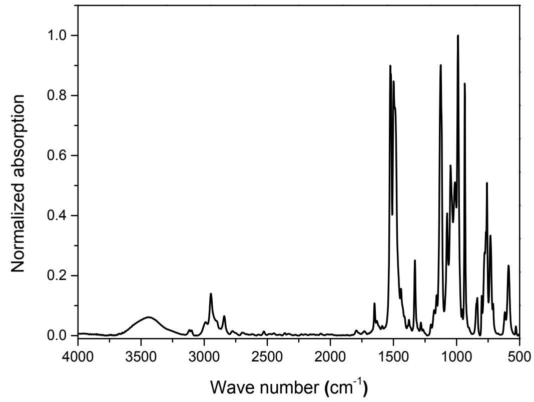

Figure S48. IR spectrum of **2-Gd**

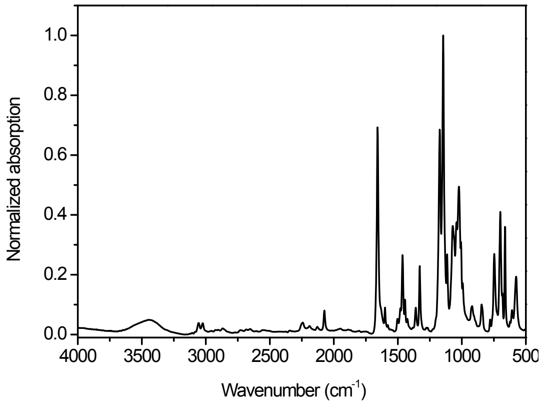

Figure S49. IR spectrum of **[D18]-3-Yb**

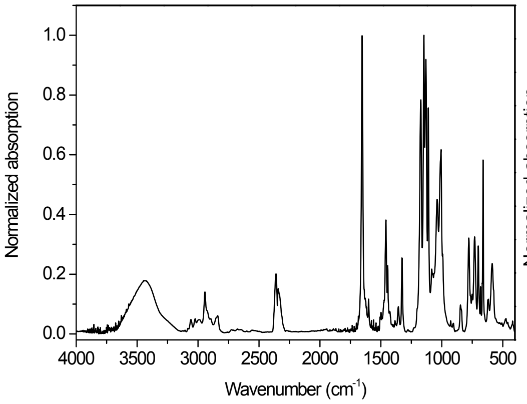

Figure S50. IR spectrum of **3-Gd**

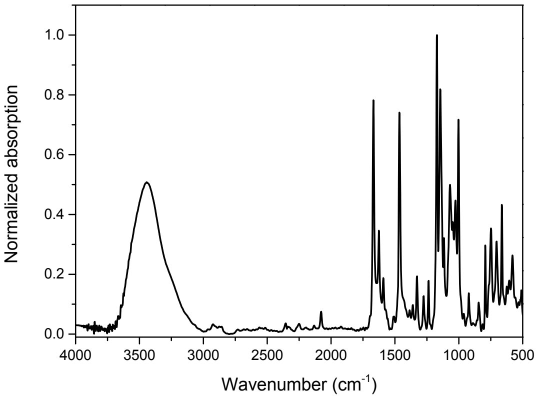

Figure S51. IR spectrum of **[D18]-4-Yb**

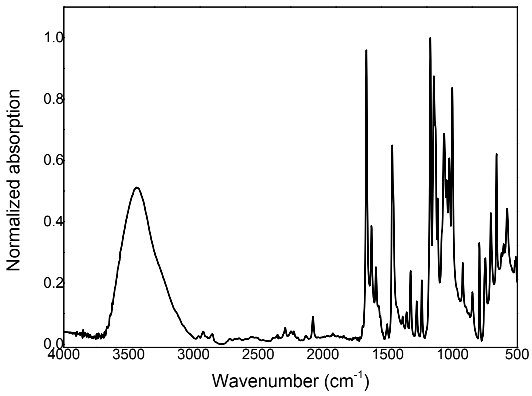

Figure S52. IR spectrum of **[D$_{18}$]-4-Gd**

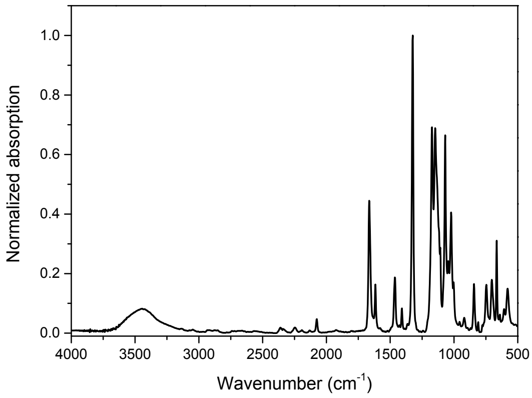

Figure S53. IR spectrum of **[D$_{18}$]-5-Yb**

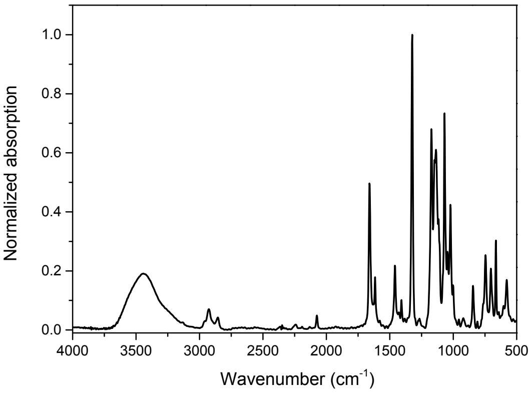

Figure S54. IR spectrum of **[D$_{18}$]-5-Gd**

3. Photophysical properties

3.1 General

Emission and excitation spectra were measured on an Edinburgh Analytical Instruments FLS920 lifetime and steady state spectrometer equipped with a 450w Xe lamp, a 5w microsecond flash lamp, a visible photomultiplier tube (PMT) detector (200−870 nm, R928P; Hamamatsu), and a NIR PMT detector (800−1700 nm, R5509–73 with C9940–02 cooler, Hamamatsu). Excitation and emission spectra were corrected for instrumental functions. Low temperature spectra were recorded on frozen glasses of solutions of Gd complexes (MeOH/EtOH 1:1, v/v) using a dewar cuvette filled with liquid N_2 (T = 77 K). NIR Quantum yields in CH_2Cl_2 and CD_2Cl_2 were measured by the optically dilute method using Yb(TPP)(LOEt) as the standard after excitation at λ_{ex} = 425 nm (2.4%, CH_2Cl_2 solution) according to the following equation:

$$\Phi_s = \frac{k_s}{k_r} \times \left(\frac{n_s}{n_r}\right)^2 \times \Phi_r$$

Where the subscripts *r* and *s* denote reference and sample respectively, Φ is the quantum yield, *k* is the slope from the plot of integrated emission intensity *vs* absorbance, and *n* is the refractive index of the solvent. The estimated error of quantum yield measurement is 15%.

The absolute quantum yield of **[D$_{18}$]-1-Yb** was determined in an integrating sphere on a FLS-920 (150 mm in diameter with the inner surface coated with PTFE to enable efficient scattering of light) according to the following quation:

$$\Phi = \frac{A_{em}}{(A_{scatter}^{ref} - A_{scatter}^{sample}) \times k_{Red/NIR}}$$

where A_{em} is the integrated area of the sample's emission (corrected); $A_{scatter}^{ref}$ and $A_{scatter}^{sample}$ are the integrated area under the Rayleigh scattering peaks of the reference sample and the sample under study; and $k_{Red/NIR}$ is the ratio between sensitivities of two detectors, which was determined right after the measurement.

The quantum yields of **[D$_{18}$]-1-Yb** were also re-determined on a Horiba-Jobin-Yvon Fluorolog-3 spectrofluorimeter equipped with a iHR320 Imaging Spectrometer, a CCD detector (1024× 256 pixel, 200-1100 nm) and a Quanta–φ integrating sphere (150 mm, PTFE inner sphere) referenced to Yb(TPP)(LOEt).

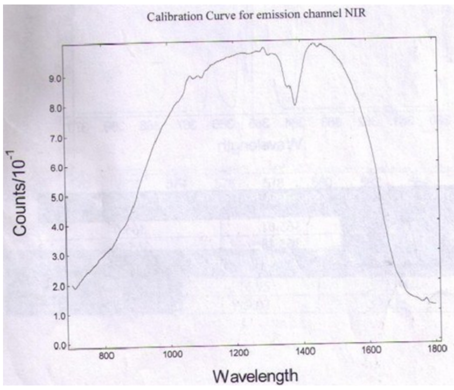

Figure S55. Calibration curve for NIR emission detector.

3.2 Absorption, excitation and steady-state emission spectra

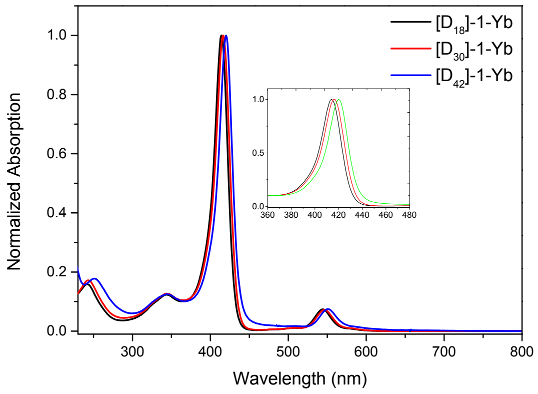

Figure S56. Normalized UV-vis absorption spectra of **[D$_{18}$]-1-Yb**, **[D$_{30}$]-1-Yb** and **[D$_{42}$]-1-Yb** in CH_2Cl_2.

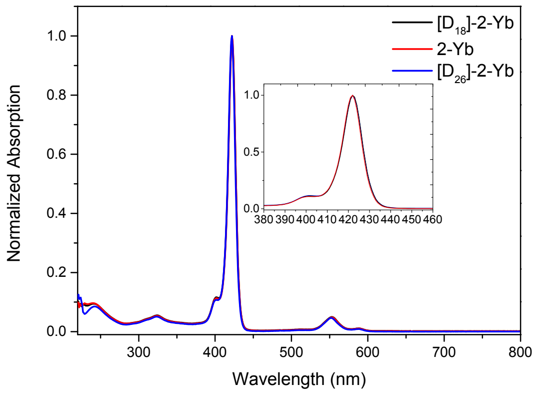

Figure S57. Normalized UV-vis absorption spectra of **2-Yb**, **[D$_{18}$]-2-Yb**, and **[D$_{26}$]-2-Yb** in CH_2Cl_2.

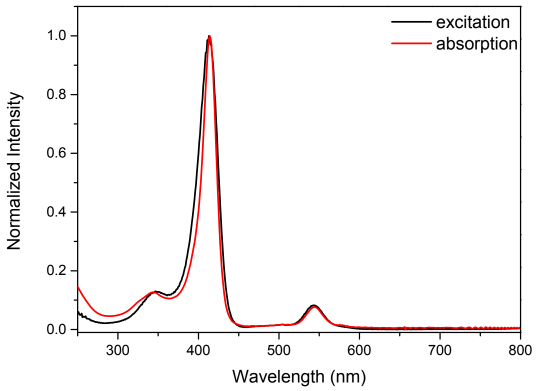

Figure S58 Normalized excitation absorption spectra of **1-Yb** in CH_2Cl_2 monitored at 975 nm.

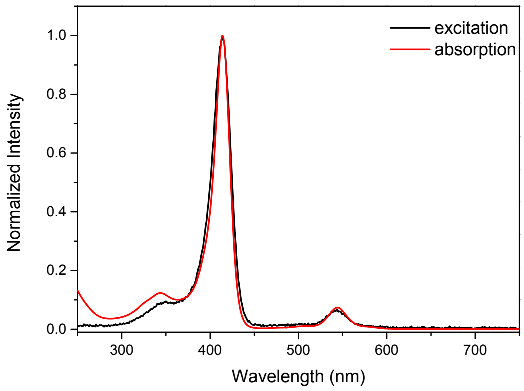

Figure S59 Normalized excitation absorption spectra of **[D$_{18}$]-1-Yb** in CH_2Cl_2 monitored at 975 nm.

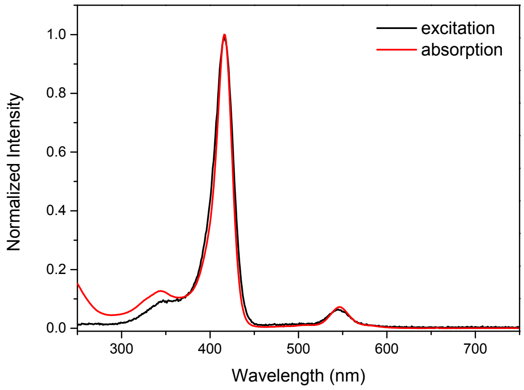

Figure S60 Normalized excitation absorption spectra of **[D$_{30}$]-1-Yb** in CH_2Cl_2 monitored at 975 nm.

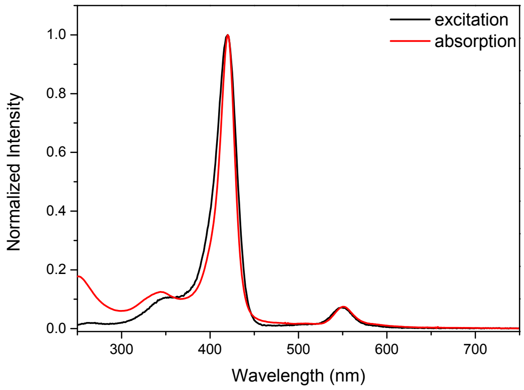

Figure S61 Normalized excitation absorption spectra of **[D$_{42}$]-1-Yb** in CH_2Cl_2 monitored at 975 nm.

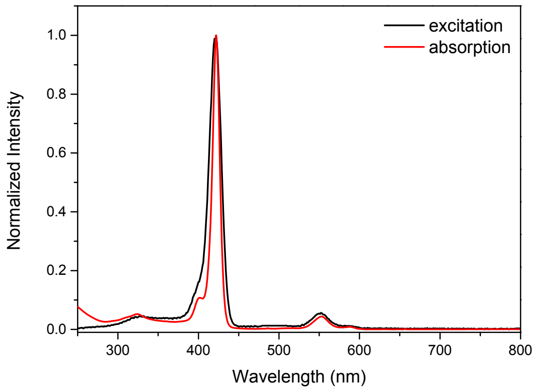

Figure S62 Normalized excitation absorption spectra of **2-Yb** in CH_2Cl_2 monitored at 975 nm.

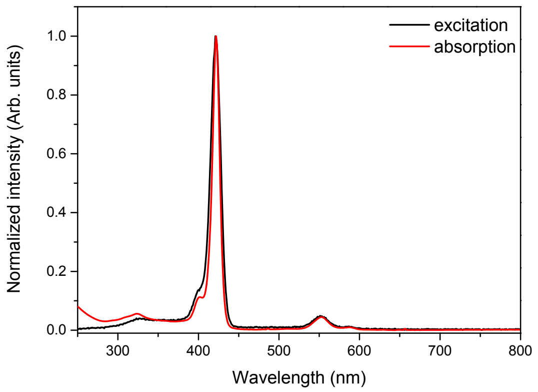

Figure S63 Normalized excitation absorption spectra of **[D$_{18}$]-2-Yb** in CH_2Cl_2 monitored at 975 nm.

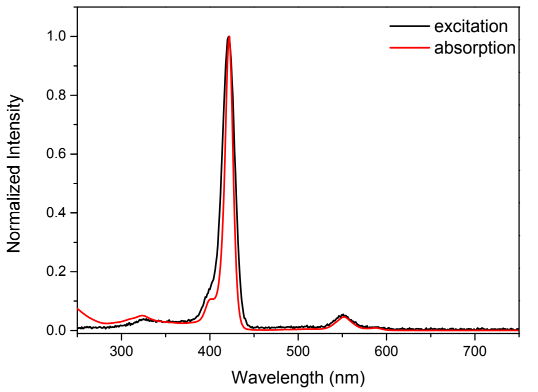

Figure S64 Normalized excitation absorption spectra of **[D$_{26}$]-2-Yb** in CH_2Cl_2 monitored at 975 nm.

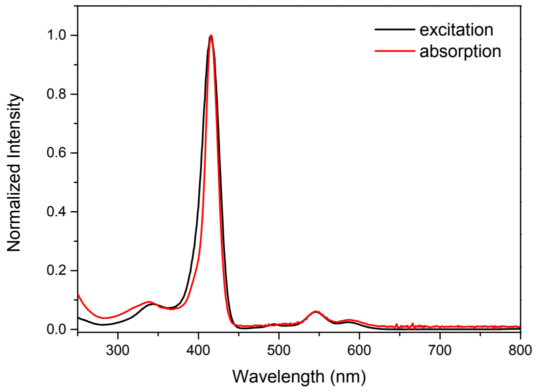

Figure S65 Normalized excitation absorption spectra of **[D$_{18}$]-3-Yb** in CH_2Cl_2 monitored at 975 nm.

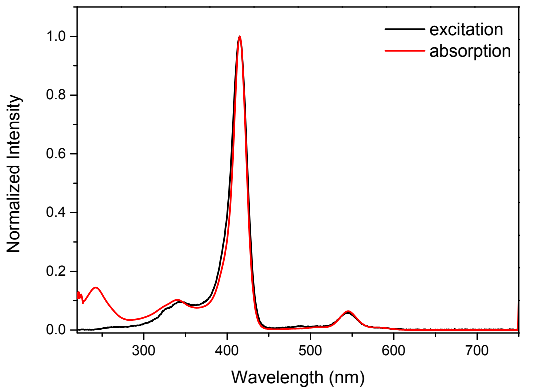

Figure S66 Normalized excitation absorption spectra of **[D$_{18}$]-4-Yb** in CH_2Cl_2 monitored at 975 nm.

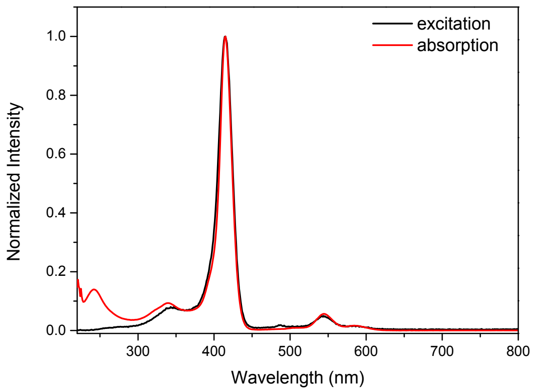

Figure S67 Normalized excitation absorption spectra of **[D$_{18}$]-5-Yb** in CH_2Cl_2 monitored at 975 nm.

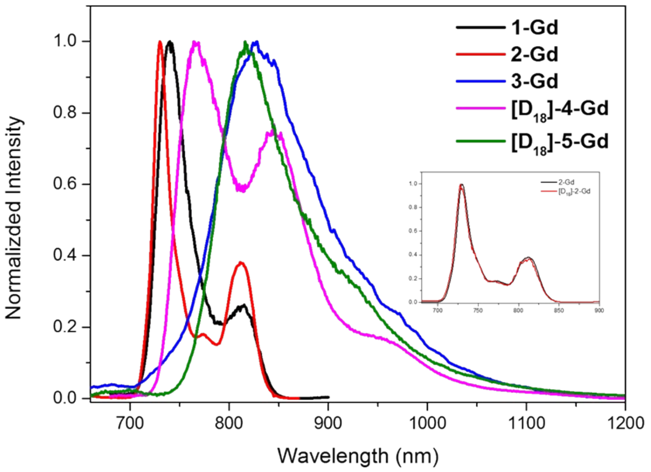

Figure S68. Low-temperature (77 K) steady state emission spectra for the transition T1→S0 of Gd(III) complexes in MeOH/EtOH (1:1, v/v). (Deuteration of Klaui ligand does not affect the emission band of the Gd complex as shown in the inset.)

3.3 Luminescence decay profiles

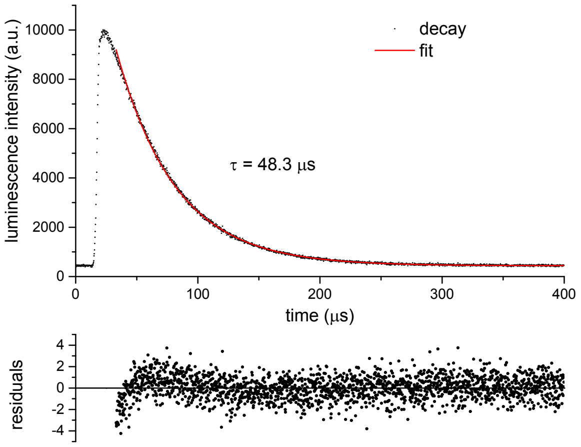

Figure S69. Luminescence decay profile of the transition $^2F_{5/2}$→$^2F_{7/2}$ in **1-Yb** in CH_2Cl_2.

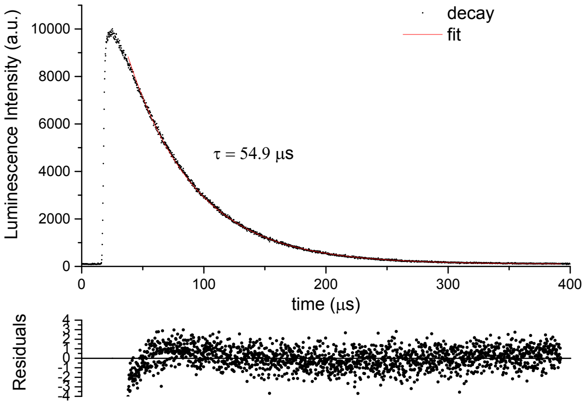

Figure S70. Luminescence decay profile of the transition $^2F_{5/2}$→$^2F_{7/2}$ in **1-Yb** in CD_2Cl_2.

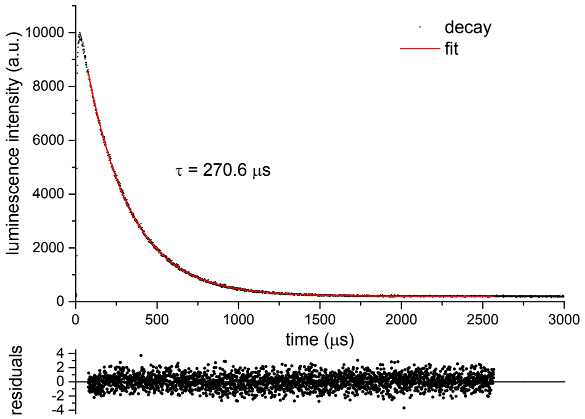

Figure S71. Luminescence decay profile of the transition $^2F_{5/2}$→$^2F_{7/2}$ in **[D$_{18}$]-1-Yb** in CH_2Cl_2.

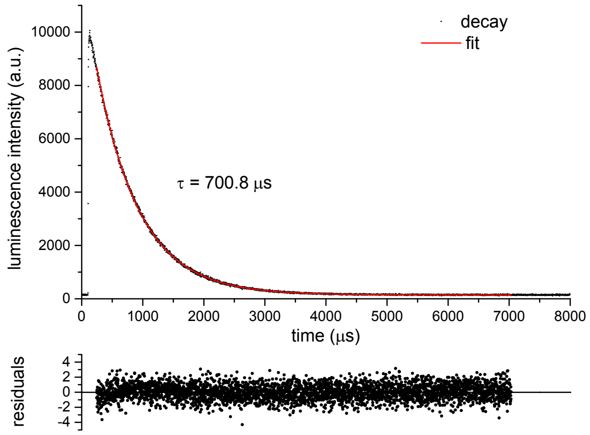

Figure S72. Luminescence decay profile of the transition $^2F_{5/2}\rightarrow{}^2F_{7/2}$ in **[D$_{18}$]-1-Yb** in CD_2Cl_2.

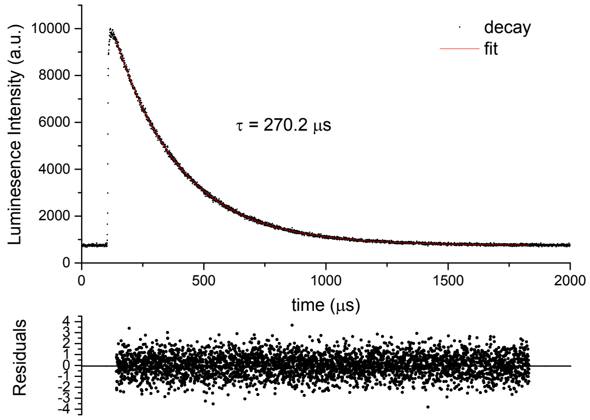

Figure S73. Luminescence decay profile of the transition $^2F_{5/2}\rightarrow{}^2F_{7/2}$ in **[D$_{30}$]-1-Yb** in CH_2Cl_2.

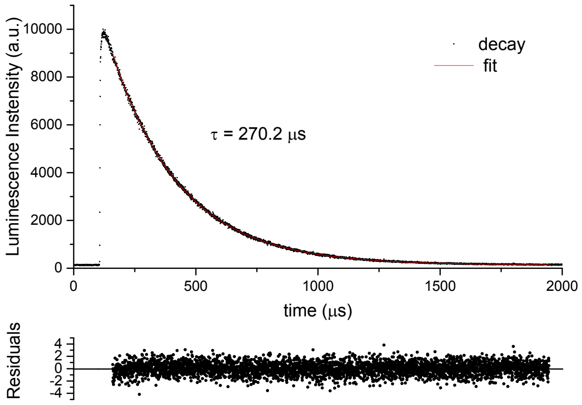

Figure S74. Luminescence decay profile of the transition $^2F_{5/2}\rightarrow{}^2F_{7/2}$ in **[D$_{42}$]-1-Yb** in CH_2Cl_2.

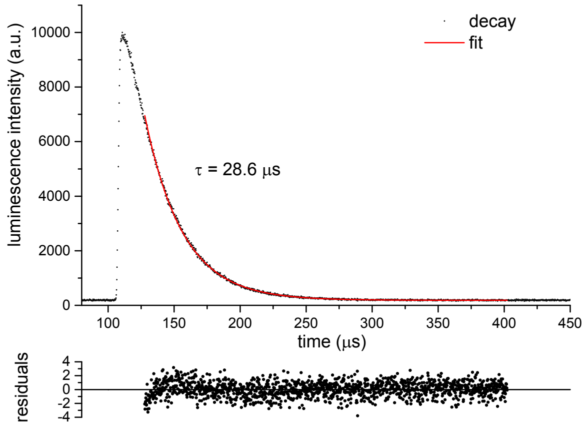

Figure S75. Luminescence decay profile of the transition $^2F_{5/2}\rightarrow{}^2F_{7/2}$ in **2-Yb** in CH_2Cl_2.

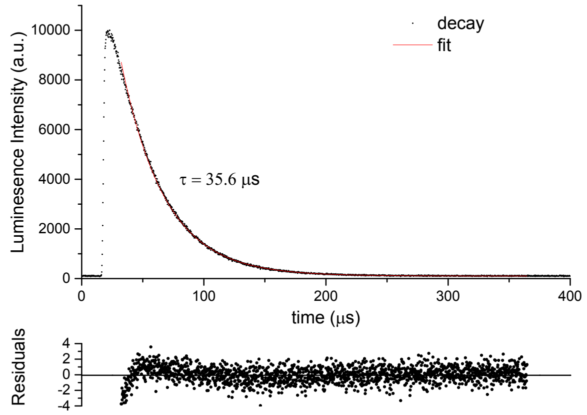

Figure S76. Luminescence decay profile of the transition $^2F_{5/2}\rightarrow{}^2F_{7/2}$ in **2-Yb** in CD_2Cl_2.

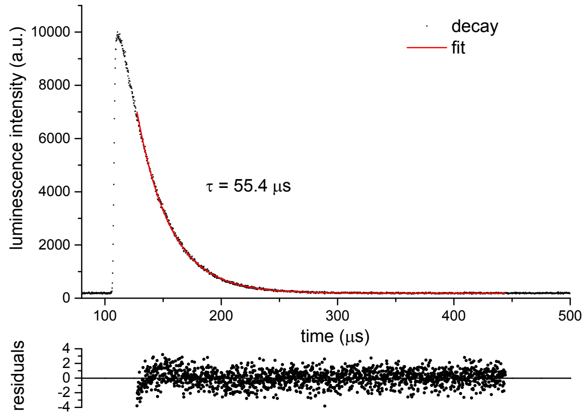

Figure S77. Luminescence decay profile of the transition $^2F_{5/2}\rightarrow{}^2F_{7/2}$ in **[D$_{18}$]-2-Yb** in CH_2Cl_2.

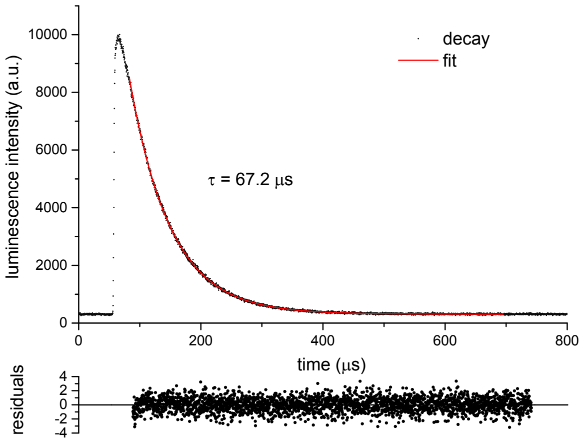

Figure S78. Luminescence decay profile of the transition $^2F_{5/2}\rightarrow^2F_{7/2}$ in **[D$_{18}$]-2-Yb** in CD_2Cl_2.

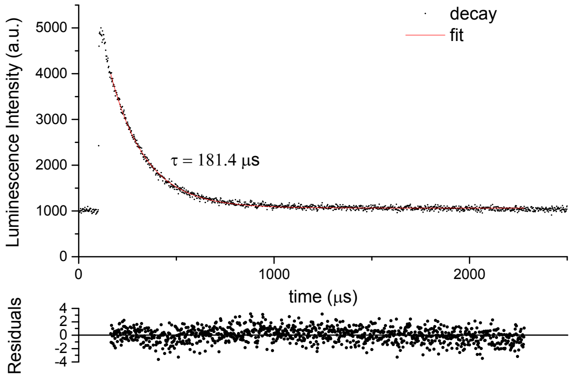

Figure S79. Luminescence decay profile of the transition $^2F_{5/2}\rightarrow^2F_{7/2}$ in **[D$_{26}$]-2-Yb** in CH_2Cl_2.

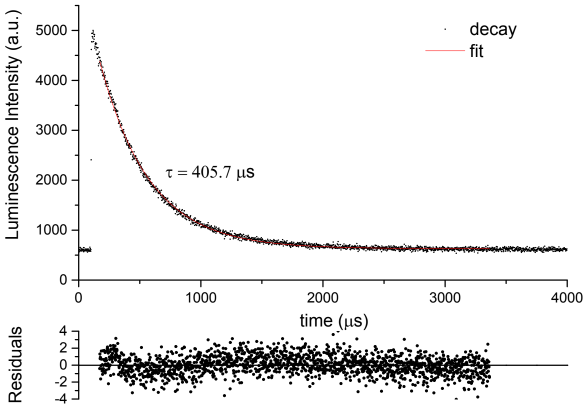

Figure S80. Luminescence decay profile of the transition $^2F_{5/2}\rightarrow^2F_{7/2}$ in **[D$_{26}$]-2-Yb** in CD_2Cl_2.

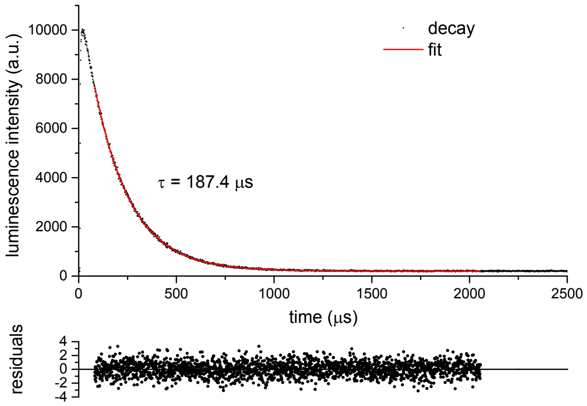

Figure S81. Luminescence decay profile of the transition $^2F_{5/2}\rightarrow{}^2F_{7/2}$ in **[D$_{18}$]-3-Yb** in CH_2Cl_2.

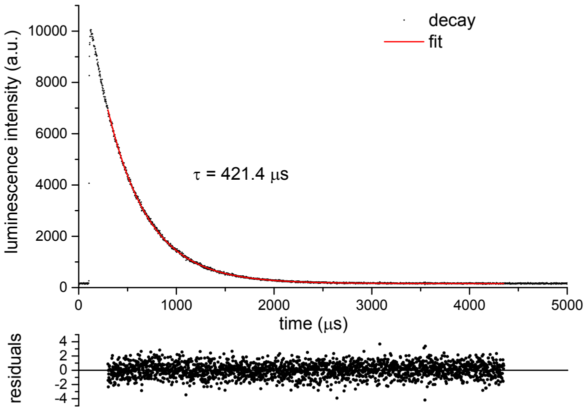

Figure S82. Luminescence decay profile of the transition $^2F_{5/2}\rightarrow{}^2F_{7/2}$ in **[D$_{18}$]-3-Yb** in CD_2Cl_2.

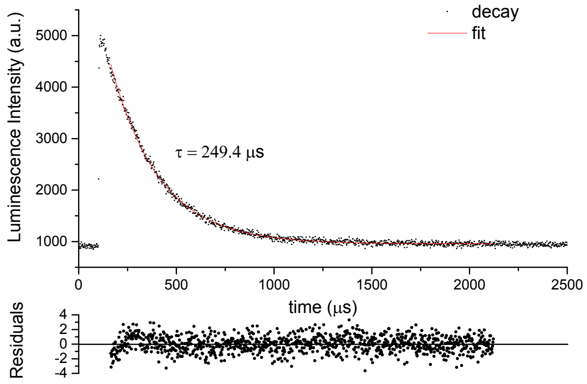

Figure S83. Luminescence decay profile of the transition $^2F_{5/2}\rightarrow{}^2F_{7/2}$ in **[D$_{18}$]-4-Yb** in CH_2Cl_2.

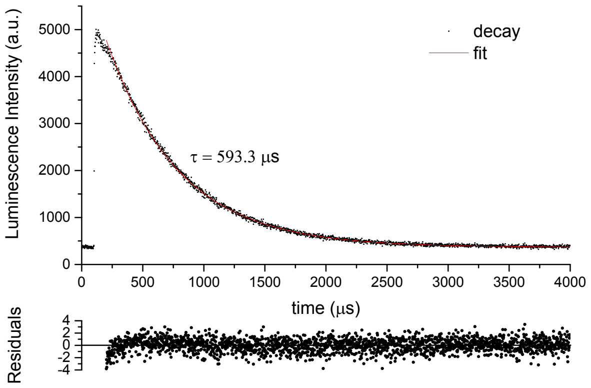

Figure S84. Luminescence decay profile of the transition $^2F_{5/2}\rightarrow^2F_{7/2}$ in **[D$_{18}$]-4-Yb** in CD_2Cl_2.

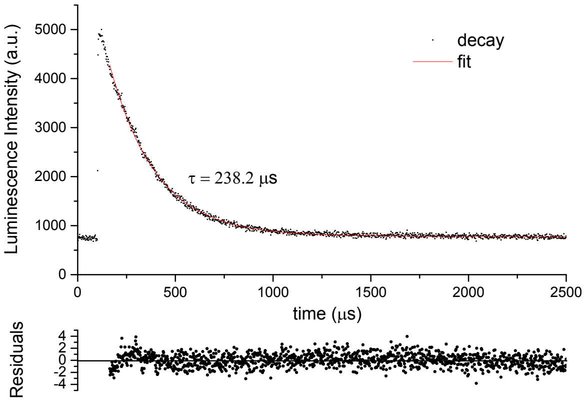

Figure S85. Luminescence decay profile of the transition $^2F_{5/2}\rightarrow^2F_{7/2}$ in **[D$_{18}$]-5-Yb** in CH_2Cl_2.

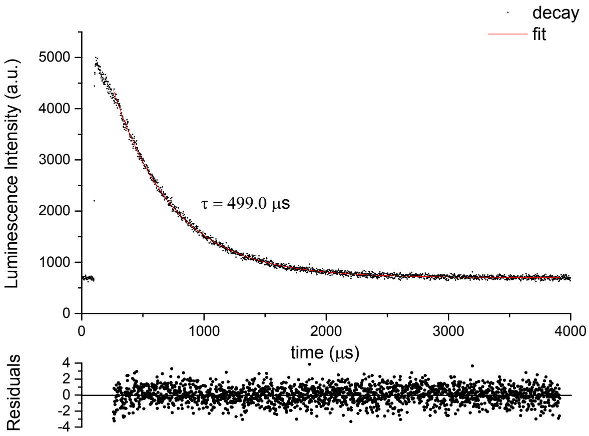

Figure S86. Luminescence decay profile of the transition $^2F_{5/2}\rightarrow^2F_{7/2}$ in **[D$_{18}$]-5-Yb** in CD_2Cl_2.

3.4 NIR quantum yield measurement

3.4.1 Results *via* FLS 920 - Comparative Method

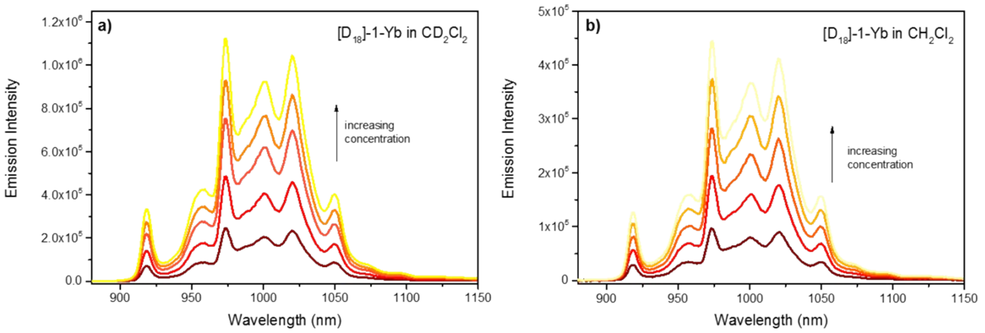

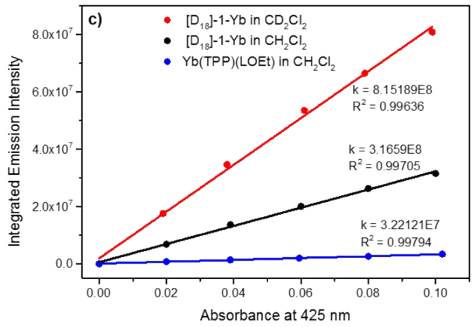

$$\Phi_{CD2Cl2} = \frac{8.15189E8}{3.22121E7} \times \left(\frac{1.4420}{1.4244}\right)^2 \times 2.4\% = 62.2\%$$

$$\Phi_{CH2Cl2} = \frac{3.1659E8}{3.22121E7} \times 2.4\% = 23.6\%$$

Figure S87. The emission spectra of complex $[D_{18}]$-1-Yb in CD_2Cl_2 (a) and CH_2Cl_2 (b) and plot (emission vs absorbance, c) for the emission quantum yield determination.

3.4.2 Results *via* FLS 920 – Integrating Sphere

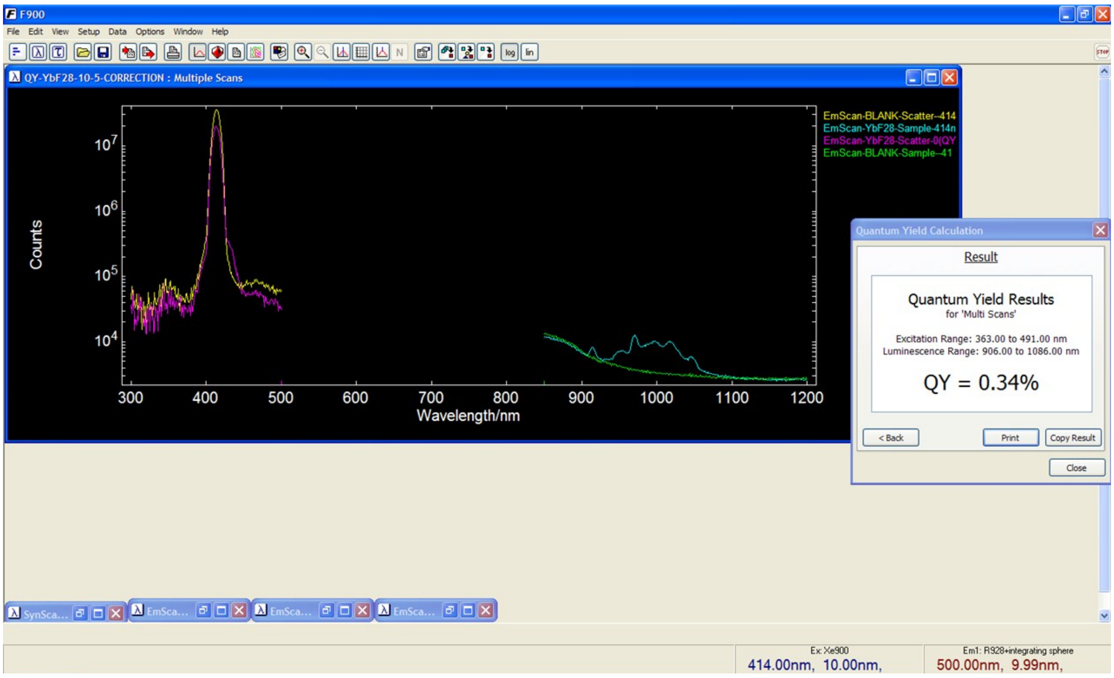

Figure S88. The absolute QY measurement of **[D$_{18}$]-1-Yb** in CH_2Cl_2 (λ_{ex} = 414 nm).

$$\Phi = \frac{0.34\%}{0.0127} = 26\% \ (k_{Red/NIR} = 0.0127)$$

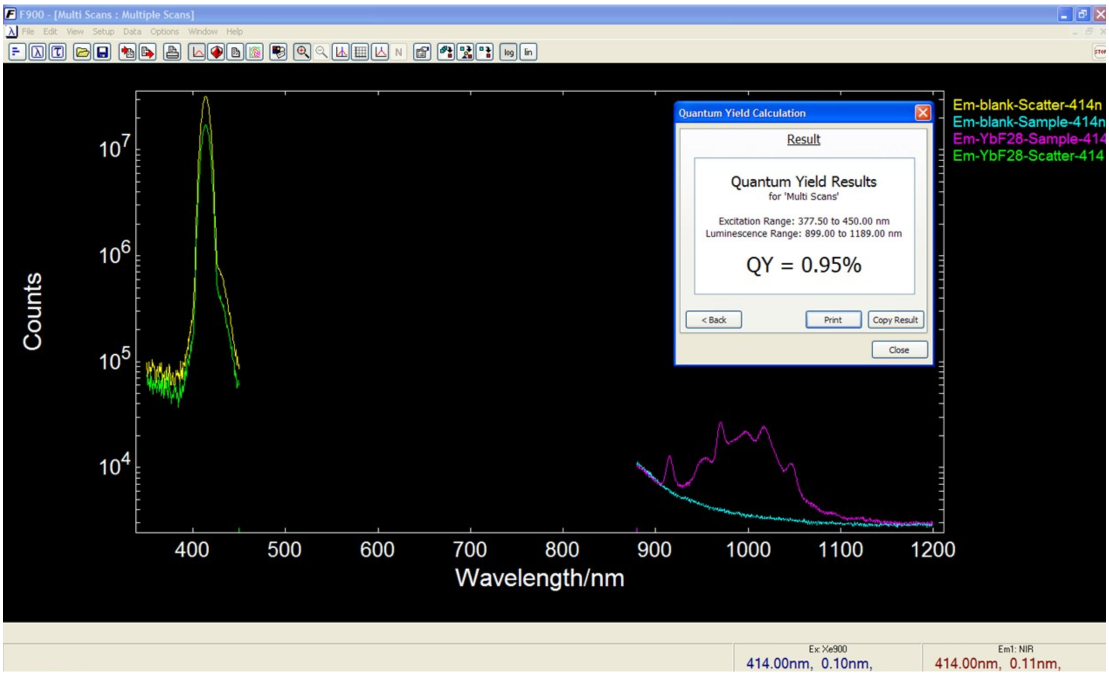

Figure S89. The absolute QY measurement of **[D$_{18}$]-1-Yb** in CD_2Cl_2 (λ_{ex} = 414 nm).

$$\Phi = \frac{0.95\%}{0.0136} = 69\% \; (k_{Red/NIR} = \; 0.0136\;)$$

3.4.3 Results *via* Fluorolog-3 spectrofluorimeter

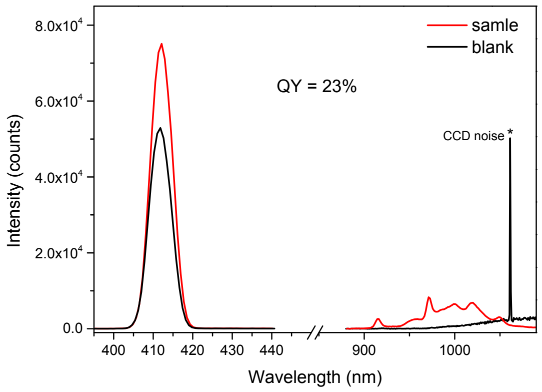

Figure S90. Quantum yield measurement of **[D$_{18}$]-1-Yb** in CH_2Cl_2 (λ_{ex} = 414 nm)

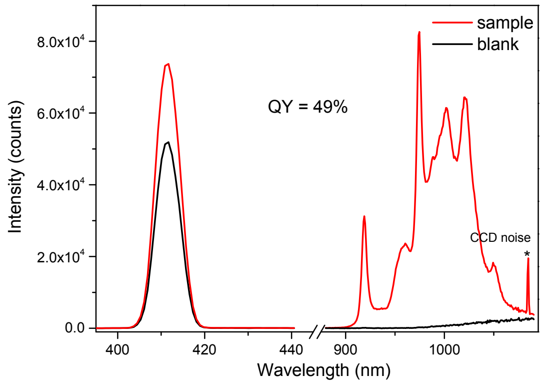

Figure S91. Quantum yield measurement of **[D$_{18}$]-1-Yb** in CD_2Cl_2 (λ_{ex} = 414 nm)

3.5 Calculation of the quenching rate difference Δk

The quenching rate difference Δk between C-(H/D) or C-(H/F) were calculated according to the following equation. The average value of Δk was given assuming that Δk of a specific site is not influenced by one another.

$$\Delta k = k_{D/F} - k_H = (\frac{1}{\tau_{D/F}} - \frac{1}{\tau_H})$$

Table S3. Quenching rate differences Δk (in ms^{-1}) for individual C-(H/D) or C-(H/F) on different sites

	Compounds used to calculate	Average Δk / ms^{-1}
Kläui ligand	**1-Yb** vs. **[D$_{18}$]-1-Yb**<br>**2-Yb** vs. **[D$_{18}$]-2-Yb**	16.1
β pyrrolic	$\Delta k_{F/H}$:<br>**1-Yb** vs. **2-Yb**<br>**[D$_{18}$]-1-Yb** vs. **[D$_{18}$]-2-Yb**	13.5
	$\Delta k_{D/H}$:<br>**[D$_{26}$]-2-Yb** vs. **[D$_{18}$]-2-Yb**	12.8
	$\Delta k_{F/D}$:<br>**[D$_{26}$]-2-Yb** vs. **[D$_{18}$]-1-Yb**	1.6
o-Ph	**[D$_{18}$]-4-Yb** vs. **[D$_{18}$]-3-Yb**	0.8
m- and *p*-Ph	**[D$_{18}$]-1-Yb** vs. **[D$_{18}$]-4-Yb**	0.4
solvent	CD_2Cl_2 vs. CH_2Cl_2 for all the complexes	2.6

3.6 Radiative luminescence lifetime τ_{rad} measurement

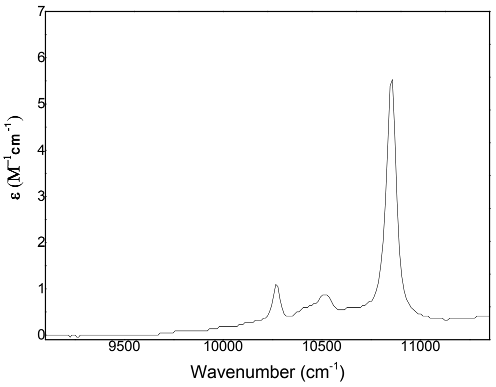

Figure S92. Quantitative absorption spectra of the f–f transitions in **[D$_{18}$]-3-Yb** (c = 22.4 mM) in CD_2Cl_2.

4. References

1 Woller, E. K. & DiMagno, S. G. 2,3,7,8,12,13,17,18-Octafluoro-5,10,15,20-tetraarylporphyrins and Their Zinc Complexes: First Spectroscopic, Electrochemical, and Structural Characterization of a Perfluorinated Tetraarylmetalloporphyrin. *J. Org. Chem.* **62**, 1588-1593 (1997).

2 Allen, K. J. H. *et al.* Lanthanide Complexes of the Kläui Metalloligand, CpCo(P═O(OR)2)3: An Examination of Ligand Exchange Kinetics between Isotopomers by Electrospray Mass Spectrometry. *Inorg. Chem.* **51**, 12436-12443 (2012).

3 Wong, C.-P., Venteicher, R. F. & Horrocks Jr, W. D. Lanthanide porphyrin complexes. Potential new class of nuclear magnetic resonance dipolar probe. *J. Am. Chem. Soc.* **96**, 7149-7150 (1974).
